# Supplementary material for: “When we have served meat, my husband comes first”: A qualitative analysis of child nutrition among urban and rural communities of Rwanda
Source: PLoS One. 2024 Jul 16;19(7):e0306444. doi: 10.1371/journal.pone.0306444 (PMC11251605; doi:10.1371/journal.pone.0306444)
Supplement: S1 File — (DOCX) [file pone.0306444.s001.docx]

**Focus Group Discussion and In-depth Interview Guide**

**Guiding questions:**

**1.** What foods (herbs/plants/animals) should a pregnant mother eat or avoid and why? What beliefs determine the types of food eaten or prohibited for women during their pregnancy?

**2.** For how long are infants breastfed exclusively and what determines the introduction of complementary feeding for an infant? (**Probe**: what types of complementary feed are given to children and which ones are prohibited and why?).

**3.** What food is given to the child in their first year and how frequently do they eat in a day? What foods are socially or culturally prohibited to be given to children in their 1 year? Do your children drink milk?

**4.** In the household, how is food distributed during meals? who is served first and when are the children and pregnant women served? (Probes: what quality of foods is saved to children? What varieties of foods are available for children? When serving for example chicken/meat, who gets the big part of the meat? What is the GBV or other forms of conflicts situation within your community? How do these conflicts impact on feeding of children and pregnant mothers?

**5.** What practices do you implement to ensure childcare? How do you care for a child who is ill? What special foods do you give to a sick child? When and where do you take your sick child for treatment? What treatments do you give to your child before seeing a doctor? How did you know about these home treatments for your child?

**6**. What needs to be done to promote the health of pregnant women and children in your community?

Conclusion: Thank you for participating in today’s discussion. Your opinions are valuable to us, and we have learned a lot from you today. If you have any questions or concerns about the discussion, we are available now to answer any individual questions or hear your concerns.

Thank you and have a great day!
