# Supplementary material for: “When we have served meat, my husband comes first”: A qualitative analysis of child nutrition among urban and rural communities of Rwanda
Source: PLoS One. 2024 Jul 16;19(7):e0306444. doi: 10.1371/journal.pone.0306444 (PMC11251605; doi:10.1371/journal.pone.0306444)
Supplement: S1 Dataset — (DOCX) [file pone.0306444.s002.docx]

*“****When we have served meat, my husband comes first, must be given respect so he takes the bigger part”: A qualitative analysis of maternal and child nutrition among urban and rural households in Rwanda.***

**Data description**

The study was conducted in five districts of Rwanda. Data were collected through focus group discussion and in-depth-interviews. In-depth interviews were conducted with key stakeholders (nurses and CHWs) while focus group discussions (FGD) were done with mothers and fathers of children under the age of five. Forty (40) in-depth interviews (IDI) were conducted. In each district, 4 community health workers drawn from different sectors within the district and 4 nurses from different healthcare facilities participated in the IDI. A total of ten (10) FGDs were held – one for mothers and one for fathers in each of the five districts. Study participants included in the FGD were purposely selected with the help of community health workers.

Below are the excerpts that were obtained from the IDI and focus group discussions. The excerpts have been anonymized by removing the site from which the participated was drawn and the gender of the participants.

**Excerpts.**

*‘A pregnant woman is recommended to take green vegetables, meat, rice, fruits mostly those that increase blood in the body so that she can keep being better’.*

*‘A pregnant woman is restricted from eating sweet potatoes, corns and taking alcohols’.*

*‘the reason why a pregnant woman is not recommended to take corns, smoke alcohol and those potatoes, there are no enough nutrients in them’.*

*‘For me I think that since the woman is pregnant, she is like a sick person so she is supposed to take those corns because they also contain some nutrients but at the controlled level for if they become too much, they will affect her body’.*

*‘Too much corns, not only to a pregnant woman, even in normal life there is where one gets stomachache in case he or she consumes a lot due to their outer courts(taste) and may cause different problems. Therefore, this shows that even it can be worse to a pregnant woman’.*

*‘I would like to complement on number seven’s idea. So I think you can see that if a pregnant woman consumes a lot of corns, they will cause alcers to her, therefore, it it can be important if she consumes less’.*

*Let us start with the first question: “What foods (herbs, plants, animals) should a pregnant mother eat or avoid and why?*

*‘He who has an idea will speak and none is allowed to interfere if another is still speaking. You let the previous one ends with his idea and then go on with yours. There are no right or wrong answers to the questions we ask, we are only concerned with your ideas. So, you can start brainstorming these foods that the pregnant women are allowed and now allowed to eat’.*

*‘ what a pregnant woman is allowed to eat are: vegetables; he can eat tubers including irish potatoes, meat; energy-giving foods, and body-building foods’*

*‘the pregnant woman has to be out heavy works; he has to be working on feasible work to her.*

*‘A pregnant woman has to eat vegetable, green vegetable, including amaranth vegetable, spinach, animal derivative including eggs, milk. She can take also the body-building foods including beans; energy giving foods like sweet potatoes and irish potatoes’*

*‘ A pregnant woman has to avoid smoking tobacco’she has to avoid drinking alcohol and drug’*

*‘ A Pregnant woman has to avoid getting treatment from the non-certified physicians’*

*‘ A pregnant woman has to drink porridge because it is very important for her. In addition, she has to avoid heavy works for her safety’.*

*‘ A pregnant woman has to eat vegetable. She also has to eat fruits like passion fruit; avoid heavy works and drug’.*

*‘ We have to look after a pregnant woman. She has to get fruits, body-building food, and she has to be free from anger’*

*‘Since there are health advisors in villages, they go on training people where they show them how they can maintain vegetable farming and show those with poor diet how they can do it’.*

*‘We would like you to recoup on the question because it is holding a powerful idea’.*

*‘So according o my opinion or generally, what I can say is that for example there are some people that are not allowed to eat meat, that may be due do different beliefs but for me I emphasise that everything that has an advantage to a pregannt woman and her baby should be taken regardless to beliefs because for example when someone is sick, there are no medicines that will not be prescribed due to his or her beliefs, she is supposed to be treated accordingly’.*

*‘Thank you, according to me, to those restricted things to apregnant woman like alcohol or cigarette, I am very sure that I can not alow my wife to take them in case she is pregnant. To the bliefs therefore, we all know that eggs, fruits and vegetables could be better to apregnat woman since they contain all nutrients needed by the pregnant woman and the baby in the womb’.*

*‘I think that in case my wife is pregnant, she has to take green vegetables since they have no harm to her and prevent consuming a lot of corns, alcohol and smoke pregnant in order to make her life better and safe’*

*‘I got confused on the thing of consuming a lot of corns, we all know that our ancestors used to stock corns and beans as the main food for their daily life and they peacefully. Now may you please explain for us how those corns affect women?*

*‘even corns are among the carbohydrates, which means they can be also important to apregnant woman. We should consider that when everything is consumed in alarge amounts, it becomes worse. For me I think that she is supposed to get every thing becausese sometimes she may get addicted to these corns. Everything should be consumed in a controlled manner’.*

*‘Alcohol and tobacco can have bad consequences on the child in the womb. However, vegetable can build up the body and potatoes can give us energy’*

*‘ The reason of that belief, when a woman bears the heavy works, she can give birth to a premature born or abortion. That is why a pregnant woman has to free from the heavy work’.*

*‘ A pregnant woman has to avoid the heavy works. Again, he has to avoid much anger because this can affect the fetus’.*

*‘ We have to make sure the pregnant woman takes safe food and beverages on the cleaned materials’.*

*‘ we know that alcohol leads to bad effect in our body. That is what we really believe. That is why we say that the pregnant woman does not have to take alcohol, does not have to smoke. Instead, she has to eat this and that so that she becomes healthy’.*

*‘We believe that a woman can certainly be health only if she avoids taking drugs and tobacco. With this, the fetus will be healthy too and can grow well. If this woman takes such vegetables and fruits, the fetus will not stunt. Sometimes, women give birth to the unhealthy baby born due to the types of food they took when they were pregnant. So, we have belief that whenever a woman takes healthy food, she becomes healthy too’.*

*‘ A woman with the right belief has to eat vegetable, passion fruit, fruits, and get joy for her to give birth to the healthy baby born. This time, she can give birth to the health baby’.*

*‘usually we are being trained and councelled, there are advices given to maternal mothers either in the village meetings where the health concellors help these mothers from the time of birth to atlest two years with the effective brest feeding. Therefore, when a baby is quitted from breast feeding before two years, you are mistreating that baby’.This doesnt mean that in the whole of this time of breastfeeding, the mother should stop taking balanced diet’.*

*‘it sometimes happens when ababy is breastfeeding and before getting full, the mother removes the baby from the breast. In the few knowledge we were given, they told us that the first breast milk doesn’t contain enough nutrients as the one coming after sometime. So this can retard the growth of the baby’.*

*‘A maternal mother should breast feed the baby in first thirty minutes after birth so thet the baby can get used to it and increase relationship with the mother,then from the the time of birth to six months the baby has to be breastfed without any other thing included in the diet because the baby’s stomach is not yet able to digest the food.from the sixth month to two years the baby is given other few complements to the breastmilk like poriedge, with the maintainance of hand hygiene.that is all I can say’.*

*‘a baby has to be breastfed at anytime in need of it’.*

*‘ two years the baby can be breast fed at anytime in need of it’.*

*‘ In six months, a baby has to breastfeed exclusively’.*

*‘ And then, after six months, you have to feed the baby with fruits, porridge, and breast milk’.*

*‘ A baby has to breastfeed exclusively eight times in case he is not yet given complementary feed’.*

*‘ A baby has to breastfeed from the time he is born until six months. After reaching to six months, you start giving him complementary feed’.*

*‘ Here in village, we start giving porridge to our baby as complementary food. After the baby becomes familiar with porridge, we give him vegetable which is not the same as we adults people eat. It is the grinded and soft vegetable’.*

*‘ From the time a baby is born, he has to be feed by only breast milk. After six months, you have to feed him with complementary feed, such as fruits, porridge. Then, you make a particular cook for the baby, in which you will prepare the soft food for him. Actually, at home, a woman can prepare every type of food like irish potato, beans; but remember, a baby cannot eat such feed. This is why a baby deserves a particular cook, special to him. For this special cook, a mother can prepare soft food like vegetable and other soft feed that aligns with the baby’s age.’*

*‘ From the time a baby is born up to six months, he has to be breastfed exclusively, not even drinking water. Then after six months, he can start taking complementary feed like fruits, crushed food, but remember to breastfeed him too. Do not consider providing him complementary food without breastfeeding him! After he gets familiar with eating these soft food, you keep gradually including the semi-solid crushed food in his meal’.*

*‘yeah I would like to complement on what my colluege has said that a baby can breastfeed any time it needs to but remember you should first check whether you also have the balanced diet so that the baby can also get some nutrients from the mother’s milk. The mother can’t just breastfeed the baby without taking some porridge, balanced diet like green vegetables, proteins, and others so that the baby can grow well. So when the mother takes balanced diet, even the baby will be satisfied easily’.*

*‘ at the age if six months is when abay can start being given complements and these include fruits, yellow banans, vocado, passion fruits. There are also other sources of nutrients that the government has prepared for maternal women like flour for porridge’*

*‘at the time that a baby should quit breast, I don’t know what they can do when it happens that a woman gets pregnant just after ababy of one-year-old, so by then I don’t know which time is predicted for that young kid’.*

*‘after the six months that a baby breast feed, considering my kids that ate at five months because they used to eat infront of them and cause them greed, and you find that sometimes after breast feeding, they could cry for more food because of others’.*

*‘ when a baby is six months, it’s the time to complement for her or him to increase strength because by then breakmilk is no longer enough for him or her.so that is why they find some complements to complement to the breast milk like porridge, passion fruits and yellow bananas’.*

*‘Corns as it has been said earlier are restricted to be given to ababy as a ccomplementfor the breast’because his or her stomach is not able to digest them. yeah, they contain nutrients but they are too hard for the baby’s stomach to digest that is why she or he should be given porridge flour” shisha kibondo”, green vegetables ans sweet potatoes are also restricted to the baby becaause even though they are important, they can be hard for the baby to swallow. The baby needs soft things that can be easily swallowed and they should be given while measured.*

*“there is no predicted time for the baby to be started being given breast complements exept at sixth month because the research shows that at the sixth month, breast milk is no longer enough for the baby. So the complement is usually made of balanced diet that is mixed with different things and make them easy for the baby to consume.the porridge given to the baby should be of mixed flour. They also say that ababy below one year of age is not recommended to take milk because the stomach is not yet able to digest that milk’. Thank you.*

*‘usually in the first yea, this is when the baby is getting used to food which means she or he need that balanced diet, which includes carbohydrates, proteins and vitamins. There we will come back at the green vegetables that helps in prevention of diseases and keep in mind that even those carbohydrates should be given in a controlled way.the baby is supposed to get the balanced diet in sufficient way’*

*‘according to my beliefs, that diet given to achild in the first year should include mostly porridge with mixed flour, then to the carbohydrates, since they are too hard for the baby to chew and swallow, they can churn them and give them to the baby easily and also fruits. that was my opinion’.Then coming back to what you asked that how often can the baby be given food, usually anytime the baby needs food, she or he should have it, God forbid for the baby not to get food in case of its need.*

*‘complementing on what my college said, any time the baby needs to breast feed should have it, but a one-year baby or below should not be given food anyhow because the stomach is not yet used to digestion as an older person. We even kow that even older people cannot sustain the consumption of much food or eating every timetherefore, a one-year kid should be given food thrice a day after brest feeding for few minutes. The hygiene should be maintained to prevent some diseases’.*

*“breast feeding should have time, one should determine the time to feed the baby be because you are the one in charge since the baby is un conscious of what is allowed or not. So for me I think a mother should feed her baby atleast four times a day”.*

*‘ I cannot provide sweet potato to my baby as complementary food because it cannot serve anything to him.*

*‘ I am number five. You have to prevent a child from taking drugs for his safety and prevent him from embracing bad habits. Instead, you have to show him to right way to pass though for him to be useful to his parents.*

*‘ A baby who is taking complementary food has to take food that is clean for him to be free from dirty hand diseases.*

*‘ It is not good to feed maize to a six months’ baby. He cannot eat it, and it can serve nothing to him again. Also, you cannot give him sweet potato because he cannot chew it. Instead, you give him vegetable, sauce, and porridge. Thank you.*

*‘ A baby who is taking complementary food, as my neighbor said, he cannot eat maize because he cannot chew it. Instead, you look for a way of transforming that maize into flour for the baby to drink porridge because he can easily swallow porridge. Moreover, a baby of these months starts taking many dirty things; so, it is advisable to assure his cleanliness. With this, food for this baby has be safely prepared in order to avoid dirt hand diseases.*

*‘ We really know that a child of six to eight months has to take complementary food three times a day. And then, five times for a child of eight to one year. That complementary food has to include vegetable, fruits. In his first year, he has to eat the energy giving food, such as irish potatoes, beans, and body building food.*

*‘ This child, who takes complementary food, likes to take many dirt things from here and there and put them in his month. This is why he has to be cautiously protected. In case, he needs to eat, give him food that cannot negatively affect his life.*

*‘ A six months’ baby has to eat three times a day. If he has six months, he has to be given soft food that he can easily chew. A child of one year eats twelve spoons. If he has nine months, he eats nine spoons. It depends on the months he has from the time he was born.*

*‘ A child of a year has to take complementary food that include fruits and vegetable. Remember to prevent him the non-sense food, like drug. Prevent him from injuries for him to get good life.*

*‘. A child of one cannot eat the sweetest food. Instead, you can give him vegetable, a lot of porridge and milk, but remember to prevent him from taking drugs. Don’t give him the energy-giving food.*

*‘ It is advisable to give enough care to those children under one year. Give him soft food because his stomach is unable to digest the solid food. Avoid him from consuming alcohol. Remember to track his weight time to time in order to check how the child is growing up. If you find that he is not growing well, you try to improve on the diet you give him. If you find that the child is in good track, you keep feeding him accordingly.*

*‘ Something to add on is add cleanliness in that child’s food.*

*‘we can say corns, sorghum that is not flour, all those are not allowed to be given to a one-year baby’,the reason is that the baby at that age is too young to chew, swallow and digest them’.*

*‘ in our culture we know that such a young kid is not supposed to take those foods’.*

*‘may be complementing on what you said, I think this is realted to our culture, not only culture, even as mature people, you can tell, usually it is a taboo to give a young kid alcohol by then, you will be killing that kid. But it sometimes happens where some kids are raised by alcohol which is a problem’.*

*‘for me I will need your support because maybe I am not experienced in this. Since I just have onlybone kid, you can please share with me your experience and I can be yiur good ambassador’.*

*‘ I am a man I will say about my own experience because you can’t know someone’s experience. In the morng my wife wakes up warms the food and give it to the kidsthen after thirty or fourty minutes, she gives them porridge and I also have some so that my kids can have appetite, at lunch and supper we get food, but for the kids it is special because they normally say that the stomach of the babies under two years are not like those of adults, there is their diet. All of that is accompanied by hygiene and we always emphasize o the balanced diet. we were blessed to have to afford anomal products like milk, eggs, and silver fish. I give them milk because they are already two years’.*

*‘ In the morng we get porridge, and get food at lunch and supper’.*

*‘we usually know that pregnant mothers and their babies have a very strog relationship. Pregnant mother is said to get hungry so much, it is clear since they are now two, even the one in the womb. So before giving me food they have to first feed my kids and even the pregnant woman has to be taken care of as they keep givimg her the balanced diet’.*

*‘ In my household, the child is served first. But before preparing that food, it is okay to wash hands and wash food too. Then, the child is served first when food is ready. After he got fed up, you serve other as well.*

*‘In my household, I serve the children first; others are served lastly.*

*‘Only a child is the one who has to be served first, and others have to be served next.*

*‘We told you that we are cultivators; we are saying what we know is right for us. We also told you that a child of one year has to eat five times a day. You have to feed three times a child who is six to eight months. However, in reality, when we are on field, cultivating, a child can only eat once a day. Why? Because of working for so long. Yet we saw that when a child started to take complementary food and is six to eight months, he has to eat three times a day. So, if we start to feed him once, it is the time he starts to stunt. Therefore, we ignore it, yet we know it. Though we said that a child who has one-year-old does not have to eat maize, sweet potatoes; sometimes, I feed the field workers (cultivators) with those food, and I give the child the same food too. I do this because I feel laziness of cooking special food of the child. With this, the child start stunting, yet we know the root cause of this stunting.*

*‘to be containing a balanced diet’.*

*‘the available diet for the kids are the vegetables’.*

*‘ it is possible that we are all at a different level to afford the whole diet. They usually say that ababy should eat and be satisfied but consume food containing balanced diet, but if you just give only potatoes so that she or he can be satisfied, by then you are just giving that baby only one thing out of many you should give. Maybe if I llok at our district, the mostly grown food is potatoes, but not only all of the people have them, but they can be easily afforded. So, we can get those green vegetables and potatoes, find some eggs and sometimes milk and small fish then try to give them to our kid so that they can get a balanced diet’.*

*‘The major source of balanced diet is usually vegetables, for those who are able can afford fruits in markets. To the issue of porridge, the government has made it easy for us’.*

*‘ It is beans, potatoes and green vegetables only’.*

*‘I kept giving examples on my family. the most available diet is patatoes with green vegetables and beans but not everyday. I sometimes find some silver fish and mix them with the soup for the kids and make them easy to consume, it has been easy today the government has brought for us shisha kibondo, so porridge is available. There is also some money that is there to complement and make it easy to afford fruits and other needs’*

*‘So, there are things that have changed now, right? I used to hear that women could not eat meat back in the days, especially those of a goat. But the research has shown that it was because of greed. But it is also understandable that in the household according to how the chicken has been gotten, there is always somebody who provided, isn’t it true? And in order for that person to be able to afford the chicken, of course he must have worked for it. After all he didn’t eat out but choose to share it with his family. It should be understood that the man should get the biggest piece as he is the who worked hard to get the chicken, but also children should get their share. In addition to that, people are different, not every man does that, because according to different cultures and people’s behaviors, I know one man that used to bring meat at home and could prepare it by himself which so that he could serve each and everyone according to what he wants. There are people who say “no”, I am working for my children so that they can grow well, before you serve me first serve my children, I’ll each after I see that they are satisfied, right? So, we should consider both situation because it is the reality in the society, and it’s there, it is how it is.*

*‘ I cannot bring my chicken and let it to my child as he/she has no idea where I got it. If I bring a chicken, it’s not only the chicken but I also bring with it a kilogram of rice, two kilograms of potatoes, and some bunches of bananas. You get that all of those things are complementary. If I bring the chicken, I have to get some piece of it, and I think that if I eat like two legs, it is not much. Then the child can get his/her share to the remaining parts of the chicken. If I have for example three children, I think each can get two pieces and their mother also could get two pieces if she wants, but the two legs should be my share as I am the one that who has provided the chicken for them all. But in brief, it’s not only the chicken I bring, but also with it I bring something else, but in my understanding the two legs should be mine’.*

*‘ For me I think that my opnion some people have mentioned it, concerning the chicken, children and their mother. You could bring it and go do something like shepherding while she is preparing it, then maybe they should not restrain themselves from eating it because you’re not around. For me, I think that the woman and the children should be the ones to eat more than me as the man of the household’.*

*‘ Only the man takes a big part.*

*‘ As my neighbor comes to say, mainly, the man takes the big part*

*‘As a woman, you serve the big part to a man. Because sometimes, a man tells you:” I bought this meat for my children to eat,” do you listen? You want to allocate a big to a man, but instead, he needs his children to eat that meat. Yes*

*‘ When the chicken is there the one who gets the biggest piece is the man because he is the one who has bought it’.*

*‘ We are in discussions here, and it is true that most of the time the man cannot buy meat and the child gets to eat first. Instead, his wife might eat first because she is the one that get to know if the food is ready by tasting it, but the biggest part should go to the husband. But it is possible also that your wife cannot leave some meat for you, that’s my idea. So, for her to solve the problem she might keep some because if you provided for your family, you should get the biggest share’ .*

*‘ At my home when I bought meat, it’s not a big deal. My wife prepares it, and when it’s ready she puts it on the table and we all eat without saying that she is not the one who provided.*

*‘ For me when I got the chance to afford the meat, my wife prepares it and when it’s ready, it’s understandable if it’s the culture or respect, she always gives me a big portion of it and I take it as it is just respecting me. And then depending on the amount of meat I brought home, after I finish eating, I give the rest to my children and their mother. Of course, I don’t eat alone I eat with my wife, but we give the leftovers to the kids’.*

*‘ Telling the truth, however things may go, when you bought the meat, present or not present you cannot get the same portion of it as the children. My wife must keep a big one for me present or not present. If it’s five pieces, each child should eat one and I get the remaining three’.*

*‘Still on that, I cannot go against the opinion of others, but the way I see it when you provided the meat you cannot sit beside your wife and monitor the preparation process in order to be sure that she doesn’t eat some while preparing, nor can you weigh it when it’s ready to eat to make sure that the preapared amount equates to the amount you brought home, you cannot do that. If you’re a reasonable man, you cannot make an investigation trying to find out if your wife has kept three pieces out of five. It should not cause any problem. Instead the man should eat fewer meat than those who have prepared it. Everything must happen in the kitchen and you stay put waiting for whatever they’ll bring to you, you don’t have to eat a bigger portion than them’.*

*‘Of course there the biggest part goes to the man but sometimes there might be a child that eat too much. A child that is strong that he/she can eat his/her share and then yours’.*

*‘ When it’s a child who is ready to start school, the first thing you do is take him/her to school. Then you teach them hygiene and good manners. That is what shows that you care for the child’.*

*‘ Concerning child care in our households, for example I have a three-year old child. The first thing I make sure he gets is food. Food is essential in a child’s daily life. If a child did not eat, I don’t know if you can wash him, even when you dree him it can be visible that he has a problem. First of all, child care starts by giving them enough food. The second is making sure that they are not lazy by giving them tasks at home like doing chores, but those that fit them which may not be considered as child abuse because even the Rwanda Law forbids it, for example taking a seven-year-old child to carry bricks, even that’s the cause for childhood stunting, it does not only come from malnutrition but also from heavy works given to children. So, we have to prevent that’.*

*‘ Of course, it happens! If you are doing some kind of research, you once come on the site and we will help you. You might find children that are being abused and you get confused. Child care should include avoiding giving them heavy works that may cause childhood stunting both physically and mentally. In addition to that, when you have a child at home as a parent you should reasonably make sure that he ate because hygiene is a must, and as it was mentioned if the child is at the age of going to school, you must provide him with everthing necessary for him to go to school’.*

*‘First of all, you must pay health care for him, and then take him to a health center for tests to know what he is suffering from and get medications’.*

*‘Still on that, in our local communities, we have community health works, when your child gets sick, you don’t immediately take him to the health center but to the community health work so that he can give him first aid’.*

*‘ When a child becomes sick you should give him a sweet potato and roast a corn for him but because he is sick, he also needs soft meals like legumes, bananas and oranges so that he can get appetite. You should try to give him food that protect the body by boosting the immune system, and then you take him to get medical care’.*

*‘ For me when my kids get sick because they lose appetite but like porridge, I try to get them porridge of different flours, then I give them soft meals with dried tiny fishes. ‘*

*‘ It is understandable when you are rich, I’ll come back to what my colleague said, you should have health care. That’s essential because when you have it the child cannot get too much sick at home which should be understood that you should take him to the hospital because they ae able to help him. Every time a child or anyone is sick, he/she hould be taken to the hospital. You should not wait for him to get too much sick in order to take him to the hospital’.*

*‘ When a child under five years old gets sick you have to take him to the community health work immediately, there are pills that they give him to help him get to the hospital without any problem’.*

*‘ It is taking him to the community health work, that’s the first aid we are trained about.*

*‘ When a child is sick you immetiately notice the change compared to how he normally was when he was okey. And when you notice that you should be quick providing him with the aid of taking him to the community health work and gives him pills that would help him get to the hospital before getting too much sick’.*

*‘ Those pills that the community health work give are the ones that help the child before taking him to the hospital’.*

*‘ Even though you cannot get potable water everywhere but it is available. Where I live it doesn’t take you a long journey, it’s just three meters or four to get the water. I grew up drinking source water but maybe our mothers or our old brothers can tell us about it, we are not all at the same age. They might have lived a primitive era where they could not get potable water near where they lived, but for me according to what I know from the time I started noticing everything, we were able to get potable water without any difficulties’.*

*“ Now my family has access to potable water because there is a tap and community health works that always encourage us to drink water especially boiled one because it’s when it becomes safe for drinking. But it’s only for four years we are able to have access to potable water in our livelihood’.*

*‘ My family had been given water filter even though it’s getting old, we could put tap water in the filter and it becomes potable drinking water safe for drinking’.*

*‘ For me where I live, we can now have access to clean water. We used to fetch water from Kagoyi river, some was flowing and other from taps. Sometimes later they brought water pump, they installed some taps as my colleague mentioned about it. Before they could let us use the tap water, they provided us with water filters to make sure we are able to get drinking water. That from the taps they encouraged us to boil it before drinking it’.*

*‘We don’t even go to the river to fetch water because water tanks are full or we could trap water for ourselves’.*

*‘ Yes I do have a toilet’.*

*‘Thank you! For me the latrine I use at home has a door and it is contructed in bricks and cement. We put water in a jerrycan so that when you finish to defecate you pour the water inside to flush down the feces’.*

*‘ All of us in the society we are not on the same level financially. For me I cannot say that my latrine is good, it is hundred percent clean but it’s a normal latrine. There is nothing special about it’.*

*‘ For me my latrine has a door and it is plastered and it has a roof. There is always water inside though not all of the people here use water in their latrines. There are people that say that they cannot share food with muslims because they use water to clean themselves, but we found out that it is better to use water than using papers because when you use water even your latrine takes long to get full’.*

*‘Thank you! For me my latrine is plastered with cement and I use water’.*

*‘We use basins or cups to wash our hands. There are not many homes in which you can find the step and wash, I only see them at the church, at restaurants but in my livelihood, I’ve never seen a step and wash’.*

*‘ At home we use the step and wash’.*

*‘ If you don’t have anything to add now tell us, is there always a soap?*

*‘ The main challenge we meet so many times is that even though we have taps near us, we could spend a long period of time without water in the taps. Then you hear people saying that the pumbs are not working and so. Then when that happens, we go back to using the water from the tanks’.*

*‘ Because we are used to those challenges, where I live many homes have water tanks. When you can afford one you buy it because you cannot always rely on tap water. You can even use a sheeting and fill it with water’.*

*‘ You have to sensitize people so that bad mindsets are eradicated. There are people that don’t care about anything. People that seem not to think properly. Let’s say in order to help a pregnant woman and her child to get proper meals, you should provide them with foods that contain vitamins, but some people seem not to give value to that. It should be understandable that sensitization is crucial to make sure that people understand that it’s necessary to take care of pregnant women and their children as well as themselves. If they get good mindsets even their children will be healthy.*

*‘We have to start by hygiene because the food and hygiene go along. When you gave both, then the mother and her child become healthy.*

*‘The government has done a good thing by bringing about the policy that encourages us to take care of our pregnant wives and the children, even though it’s not hundred percent. They trained community health works and then they trained people obout nutrition but because they have a lot on their plates, they don’t reach to everyone or do their job as it is supposed to be done. My opinion is that they should give them some kind of a salary so that they can focus onn their work. Another thing is that not every family has access to clean water in my neighborhood. There are only two water taps in our village and most of the time there is no water, it sometimes comes and then goes. For example, for me to get water I have to walk one kilometer to get clean. It means that if my wife took such a journey to fetch water or I take a 20 litre jerrycan that should be used to wash clothes, dishes and for bathing, it immediately becomes less. If we had water near us it would save us the journey and the pregnant woman would get enough time to rest.*

*‘ For the woman and the child to be taken care of properly the government should distribute porridge flours and some stipends to the pregnant women without considering the financial statuses.*

*‘ My colleague there reminded me of some money that is given to pregnant and breastfeeding women that they promised us that it should be given after every one month and half or in two months, but the challenge is that we never get the money so that those it helps those mothers. Now I hear that the money has been given after eight months, which means that it will not help the mothers and their children as it was supposed to help them because it’s late, that’s a challenge. Another thing is that the pregnant mothers that are almost due are not always available. You cannot get information a regarding the distribution of the money. It would be better if that policy is professionally monitored so that each pregnant woman benefits from it.*

*‘ About what my colleague just said, we know that a child is a child and a mother id is mother. So, sometimes you find yourself in a social status that does not match your financial status and I don’t know if it’s by mistake or something else and that has an impact on your concerning the stipends you should have been given. Finally, it causes problems to the mother and childhood stunting to the children. In the research you do you make sure that tha is considered.*

*‘ Adding to what my colleagues just said, there are things that are done with too errors and if you try to ask for help about it to those in charge of it you don’t get the help you need. That’s one and it was talked about enough I won’t say much about it again. I would like t emphasize on something that should be understood in this way; it is not always necessary that we base our survival on the money that the government gives us, we should be able to manage some problems on our own. We don’t need to wait for the support but try to provide for ourselves. We only need to change and improve our mindsets. Let’s say for example as it is said that legumes are necessary on every meal for pregnant women and the child. Do we really have to wait for the government to give us those legumes? We should find solutions before involving the government. Is it really necessary to wait for eight months? You cannot wait for that long cause you normally have the means to live your own life. This implies that sensitization is so very necessary in order to keep reminding the people that they should learn to take care of themselves. If the legumes are necessary, why don’t we culivate them in order to use them on our meals? It does not require too much land, even on a small scale you can cultivate. The sensitization should be about reminding people that they are able to do something for themselves on their own. Some people have the legumes but tthey don’t even know how to prepare them or don’t put them on every meal. So, we should change our mindsets and I believe that it will even help the government to help us’*

*‘Since you prepare when the children are by your side staring on you, they are sometimes crying, and you serve them with food which are not well cooked. Ahaha [… laughter], yes, they are served first.*

*‘ It is always better when you eat after a child is served, that is when you eat comfortably. In reality, you have to feed up the child first. After he is feed up, the child goes to play and seems happy and comfortable.*

*‘You start by cleaning food to cook to ensure its good quality. Then, you cook it until it becomes ready or adequately boiled if it is porridge.*

*‘The way we can prepare food of good quality to our children is to prepare the cleaned food. And then, we prepare it until it becomes ready. After, you put food on the cleaned dish. Then, you take your child and feeds him until he is feed up.*

*‘Once preparing food for child, when we are at home, we clean it sufficiently and cook it well. However, when we are in the field cultivating, we do not find water to clean our hands. You give your child a sweet potato quickly because there is no water, and you continue cultivating. In the time of breastfeeding, sometimes, you take a child and breastfeed him without washing your hand. You simply rub hands one another to reduce dusts. For real, we do not respect hygiene well.*

*‘ Since this region is village, we like job. When you are on the field, sometimes, you do not have water. You rub your hands and feed a child, or you give him the sweet potato for him to eat it.*

*‘. As my neighbors said, in this region, we really like working! Your hands become dirty because of cultivating for so long. However, if you reminded to bring to bring food and vegetable, you can bring a spoon too. This spoon helps you to feed a child in case you are dirty and there is no water to wash hands. When a child wants to eat, you bring that food with cleaned spoon and feed him. You can even serve the child with porridge using that spoon. You can buy those spoons to the market because there are. Arriving to the field, you open up the cup and pull porridge to that spoon and feed the child.*

*‘I can the good quality of food provided to our children, we are in the village. We are cultivators. We carry porridge and food in the small jerry can. If he cries, you put a dish full of food in his front and feed himself. After a certain, if you come back to him, you realize that he puts soil in his food and eat mixture of food and soil. No quality of food indeed!*

*‘ Among cultivators, there are the ones who cultivate for themselves and others who cultivate for others. There is no way you can go to cultivate in the field with spoon to feed your child, yet it is not your own field. You cannot do this because your boss can fire you and lose a job.*

*‘ In the way of caring to your child, you do not have to forget that spoon so that you feed your child with the cleaned vegetable. So, do you think it is normal to consider a job over your child? That is not right*

*‘When a child is sick, I first of all take him to hospital. Since he is not able to take any food, I look for the delicious and soft food and porridge.*

*‘. When a child is sick, I take him to the hospital for him to get treatment. Then, you look for vegetable, porridge, and other food he likes so that he gets better.*

*‘ When a child is sick, first of all, you take him to the hospital for him to get treatment. You try to follow the treatment as prescribed by the physician so that the child gets better. For the special treat to give him, you put in mind that he cannot eat any food. And then, you try to prepare delicious food like small fishes, fruits to see whether he can like them, which is rare to other children who are not sick. You try to look for those fruits to see whether he can like them. Then, I serve him with the porridge at the right time to see whether he will like it. That is our practice.*

*‘After realizing that a child is sincerely sick, first of all, you take him to the hospital. Arriving there, they carry out medical test or other medical related services. After figuring out what the disease is, they prescribe him treatment. Likewise, if the figure out that he is sick of the dirty hand disease, they prescribe him treatment, and recommend you domestic hygiene. You put in practice whatever hygiene practice they order you for the sake of your children to be free from the dirty hand diseases. After, you look for the soft and delicious food, such as, vegetable, small fishes, passion fruits, tamarillo, and banana’.*

*‘ You have to take the patient to the hospital’*

*‘You have to take the patient to the hospital for the physician to prescribe him treatment. You give the patients the treatment as prescribed by the physician, and remember to take care of him accordingly. But now, let me emphasize on the children who cannot speak on themselves. As a mother, you have to take care of him because there are some foods that he cannot eat, yet, you tried at your highest extent. Sometimes, we force this patient to eat certain foods because they are aligned with his treatment’*

*‘ You take first of all the patient to the hospital. If possible, you prepare porridge for him so he can eat it. You give him passion fruits; if he cannot, you try him with tamarillo. You keep changing foods until you get what he likes. This can help him to get better early.*

*‘. There are treatments that we give to a child before we take him to a doctor. Let us take example of the time he suffers from fever. You wet the cloth in cold water and put it on the child body to reduce fever. Then after, you take him to a doctor.*

*‘ When a child suffers from fever, I treat him with capsine plant before taking him to a doctor. This plant is strong at curing fever. I anoint this plant to my patient before taking him to a doctor.*

*‘ When my child suffers from fever or pneumonia, I first of all take him to the community health workers because they have some treatment. I take my child there and tell them the signs and symptoms of the disease, and they give me treatment. When this treatment does not work, I immediately go to hospital. Sometimes, I give that treatment to my child, and he ends up getting better without even reaching the hospital’*

*‘ This child I holds is mine. If he gets sick, I immediately go to the hospital’*

*‘ We all know that community health workers are nurses at the village basis. They are the right one who give us basic treatment. They give us basic treatment. This is because they have treatment for fever and pneumonia. If the child gets sick, you hurry up to the community health worker and ask him whether he has treatment for your child; he tells you. They sometimes have TDR for malaria test. He sometimes tells me that my child suffers from malaria and give me treatment. After a given time, my child gets better. I first of all reach out to him because we were mobilized that community health workers are nurses the on village basis. Sometimes, when he does not have the necessary medicine to treat the child, he gives me referral to the hospital. He does send me to the hospital without even giving me any treatment’*

*‘ If a child presents fever, I wet a cloth garment in water and put it on his body. I take the child to the hospital. If the doctor finds that the child has high fever, he tells me to put off pullovers on my child to reduce the fever. Again, he orders me to put a wet garment in water and put it on the patient’s body. I found this practice so effective because it really reduces fever. It is why I do the same practice when my child suffers from fever at home. After, I take him to the hospital’*

*‘If your child presents fever, yet you see that the hospital is far, as my colleague said, you wash a garment. Then, you wet this garment in cleaned water and put it on the child’s body parts which present high fever that other parts. If you realize that this practice is not helping the patient, you hurry up to the hospital’*

*‘. It is me who mentioned capsine plant. I am number one. Recently, my child became sick of fever, and one person came to tell me about this plant. After anointing him this plant, fever went off. That is why I use it’*

*‘ We try our best to get the safe drinking water here in the village. The way I do this, I am to say what I do. I first of all cook water until it is boiled. In this water, I put rosemary and boils together. I pull this mixture in the cleaned can. I close this can to keep water clean and safe. Whenever I feel thirsty, I go ahead and drink this water’*

*‘. We like to fetch water from the ground. However, this water is not safe ever through one’s naked eye. Once you fetch water from this place, you cook it sufficiently and keep it in the cleaned can and reserve it for drinking purpose only’*

*‘. There is no safe drinking water available here. Many persons do not have tap water, they fetch the flowing water on the ground. Some of us, who has tap water, think that it is safe, but it is not. I fetch water in can and deposit it in my house. Some use the small cans, but other use big cans. For us, we like to use the small cans. You cook water until it gets boiled, you deposit it somewhere and wait until it becomes cold. At last, you pull that water in the special can reserved for drinking water. So, everyone in the household or anyone else who needs to drink water comes and drink safe drinking water. That is the safe drinking water that I drink here in the village’.*

*‘ We have built toilet cover on roof. However, many of our toilets have no doors’.*

*‘ My toilet’s roof is covered, but the latrine’s opening is not covered’.*

*‘. I built my toilet. However, the latrine’s opening is not covered’.*

*‘ Our toilets are that ones that is build up after digging a hole’.*

*‘ I have a toilet too for my household. It is built, and its roof is covered, but it does not have a door’*

*‘ My toilet is built, and its roof is covered, but it does not have a door’.*

*‘ We use this toilet that requires to dig a hole while building it. We build it using trees. We build it using soil brick, it has a door’.*

*‘ My toilet is totally built up by trees, but its opening is not covered’.*

*‘We do not have step and wash. We wash the hands by using a bath, with water and soap’.*

*‘ You have to wash hands before cooking, serving food, even after coming from toilet’.*

*‘Since there is no step and wash in our household, we do simply wash with soap. However, sometimes you might not wash with soap, especially when you do not have it or if you are in hurry’.*

*‘ Several times, we do not wash with soap. We do not have even those step and wash in our household. No special place for a soap too’.*

*‘ I mainly wash hands when I am going to serve food to my child, breastfeed him’.*

*‘ We do not use soap several times. We use soap while washing in the evening before sleeping’.*

*‘You asked us how often we wash. I often wash as soon as I wake up’*

*‘ Here in the village, you sometimes wake up with many things to work on, and say “Oh, I will do this after coming from the field, cultivating. In reality, we wash two times day, here in the village. Most of us, we do not wash more than twice’.*

*‘You sometimes wash once a day because of the job, and you do it quickly. Again, in the morning, you go to work without washing’*

*‘ There is a difference between washing hands and whole body. A person can wash several times in a single day, but you can simply wash your body few times a day because it takes a long time, yet you hurry for a job. You simply wash your hands, face; then, you take your hoe and other facilities and go to field. After you come back home, you wash hands and body as whole. After washing, I prepare children and food too’*

*‘ Severally, we do not wash the body in the morning. As a woman, you wash the most important part of the body, like face, brush teeth, and other special parts, and go to job’.*

*‘We do not have water. There is a very long line of people at the water source. After you come from the job being tired, you face other tasks at home, like cooking, fetching water. Since there is a long line at water source, you prefer to fetch the flowing water and use it to cook. With this challenge, our children become sick of the dirty hand diseases’*

*‘We wake up early in the morning, and we go to cultivate. We come back at 5:00 pm. So, it seems difficult to carry out this hygiene in this time. It comes also another challenge of water because there are many persons on the water source. This is the reason why we fetch flowing water. This water has no quality; it is not cleaned water. You use it, yet you know that it is dirty water. It is a challenge because you use this water to clean dishes that will be used while serving food. We do it willingly because we do not have other choice’*

*‘ We have a challenge of not having cleaned water to use. We go to fetch watch water that is not clean, for real, because the compound of the water source is not clean too. There is also a very long line of people who come to fetch water there. You can see that we only drink the so called dirtiness of water. We do not really have cleaned water’*

*‘ We actually fetch water too far. We do not find water when we need it. There are many person waiting for water’.*

*‘ If we get support of getting cleaned water, we can have good lives. We can be free from drinking dirty water that can lead to dirty hand diseases’.*

*‘ Getting cleaned water can help us to fight against dirty hand diseases because when we come from job, we do not have enough time to cook and boil water. But if you fetch safe water, you can drink it without even boiling it because you are sure it is safe, according to the water source you fetched it from. If we get this water again, we can make sure we make hygiene to the materials we us, and we can feel comfortable’.*

*‘. If we get clean water, we can reduce the dirty hand diseases. Because drinking unsafe water is the origin of such diseases. The same on adult, if you drink such water, you become sick. It implies that if we get clean water, we can be able to reduce dirty hand diseases’.*

*‘. As we know, we prepare food using water; so, if we get clean water, we can prepare food well. For example, it is not better to prepare porridge for a child with dirty water. In the time we get clean water, we can boil it for our children. We can cook food with the cleaned water. With this, we can serve children food which is safe’*

*‘. It can be better if we get clean water because some of us already have it. The main challenge we are having is unclean and insufficient water. After we arrive at the water source, we wait for a long time because there are many people waiting for water. Since we live near the swamps, you go and fetch water there; and sometimes, we fetch rain water. With naked eye, water is dirty, but we fetch it because we do not have another choice. We use that dirty water to wash, for example sweet potatoes. In the same water I used to wash potatoes, I wash other foods again. This is how water become a challenge for us and prevent hygiene and sanitation’.*

*‘ If we get clean water, we eat on the right time. Sometimes, we do not eat at the right time because we wait for so long to get water at the water source; the line is very long, yet you and children are so hungry. Due to waiting a long time, a child wanders around the compound until 12:00. This time a child will not eat at the right time. Sometimes, a child falls asleep without eating’*

*‘Plants that a pregnant mother is allowed to eat, such as herbs and vegetables. animal products including meat, fish. As for crops such as potatoes, green bananas, sweet potatoes, and cassava. This is all that a pregnant woman is allowed to eat. What she is not allowed to eat I don’t know. when I was pregnant, someone told me that when you catch fish from the sea, a pregnant woman is not allowed to eat it because there is a disease, so I don't know if that is true. The reason why she is allowed to eat all that is because the baby in the womb grows well and receives the required nutrients and the baby in the womb also has a good life.*

*‘ A pregnant mother should eat vegetables, eggs, sweet potatoes, cassava and other energetic food, especially focusing on those vegetables, fish, and meat, something she is not allowed to eat especially like meat, that's how I understand it.*

*‘there are times when she eats what they call unhealthy. the meat may not be of the highest quality and the pregnant mother herself is looking for things that gives the body energy. that's how I understand it.*

*‘In a full diet, as I understand it, I think there are things that can’t be forgotten such as fruit, fish and milk, because it is also part of what gives the child a good life*

*‘ I also think that in a healthy diet that a pregnant mother should eat there must be some beans in it, because they are also good. Also fruits, especially vegetables and milk.*

*‘ beans also have important nutrients*

*‘ I also think that there are certain foods that a pregnant mother should eat for the health of the baby and herself, including those vegetables and fruits, including energetic foods such as sweet potatoes, Irish potatoes, including beans, peas, meat, eggs and fish, yes.*

*‘ I think that a pregnant woman should be taken care of by making sure that she eats well, she drinks well, she gets the porridge on time and she gets the milk. And she must also get a full meal which contains what they said, she also need to eat vegetables, sweet potatoes, cassava, she must eat potatoes and green bananas as well, it is how I understand it.*

*‘ a pregnant woman, as I understand it, they can take care of her by preparing her a complete diet such as those vegetables, because they are available to many people, and eggs if they are available, cassava, potatoes and beans and she also has to rest enough. .*

*‘, the way she should be taken care of, a pregnant mother should eat a healthy diet, energetic, constructive foods and preventing diseases foods including vegetables, including fruits, including milk and fish as well, she also has to drink porridge when necessary and eat at the right time, and after eating she need to sleep so that the baby in the womb is well nourished, you can’t eat a full diet and always sitting up, the baby in the womb will not be well nourished, and then she should also eat avocado, because avocado is a fat but very useful for a pregnant woman. good life and the child she will give birth to will be beautiful and strong, thank you*

*‘ in taking care of a pregnant woman, you must stop her from doing heavy work and stop her from tiring the body because it affects the unborn child, that's how I understand it*

*‘ when there is a conflict between the couples, the children are affected because there is no child who sees father and mother fighting then turn out to be a happy child, and then when you are pregnant and then the husband hurts you, the child in the womb is also going to be born with it, that child will not have freedom, that child will not be open to you, because he/she was disturbed while he/she was still in the womb, but when you are pregnant, and a man takes care of you and he makes sure you sleep on time, you eat on time, when there is no stress in general, the child you give birth to will born happily, it has not happened to us and no we haven’t seen it, but it happens to some. Everyone would say according to what they think, but I don't know, I can't tell you what happened, but like me, I can have a conflict with my husband. And maybe I’m pregnant and during the child’s growth there are some things he/she do and as a mother when you see those thing you can see that it is because of what happened to you when you were pregnant, and that is to say that as a pregnant mother you must be taken care of so that the child you’ll give birth to will not be traumatized.*

*“If I eat a piece of meat that was preferred by my husband and he does not find it, I am not lying, he will make me pack my things and go back to my family with no discussion, conflicts are there.”*

*“We are poor, nothing goes well, we are not happy with each other in the house, nobody wants the children to grow unhealthy but the means to afford the foods is the problem.”*

*“If my husband goes to work, instead of bringing money at home, he goes straight to the bar and uses all the money and nothing to eat in the house there will be a fight in the house. Do you think a child who does not have food can be happy? That child can also end up not growing”*

*“So, a family with conflicts has no peace, and their children get stunted easily because the child will not eat, that child will not be happy, and it will be difficult to finish food and then not grow”*

*‘I think that in the family when a husband and wife are married and they do not have a good relationship, it affects the children, it affects the pregnant woman because there are times when they fight with each other, making the unborn child not happy, and when you give birth, you see that your child likes to be alone and you see that your child is not well, because you always had conflicts at home.*

*‘a pregnant woman should lie down and rest and avoid working hard, as for the conflict when you have it and you are a man or a woman you must make sure that the child in the womb does not have any problems, and the pregnant woman also if the man leaves without giving her the money to buy food, the pregnant woman also is hurt. and secondly, when the child is in the womb, he immediately feels when you are in pain, and you as a pregnant woman, you must stay calm, that’s how I understand it.*

*‘ I also think that conflicts has the impact of the child in the womb due to the injuries that a person goes through with the child in the womb the consequences are like growing up depressed because of the pain he/she felt before he/she was not yet born, because no matter what if anything happens to us it also affects the child in the womb, and the child might be unhappy because the conflicts you had with your partner when you were pregnant, so I think that when as a mother you don’t get happiness you don’t have time to give your child happiness too, you give your child what you have, if you are happy you also give your child happiness, maybe when you are feeding your child you do it with happiness by singing to him/her I think it is in those things that makes your child happy, So, if I don't show that love to him in what I do, the consequences will come back as in that the child has not grown up well, he is crying, he is suffering and he is not happy because he did not see me that way.*

*‘ Conflict in the family can make our children not grow up well, because maybe you might be living with a bad man, who don't leave money to provide for your children which can make the children not eating on time, they don’t get that porridge too because of the conflicts you have with your husband every day. That was my idea.*

*‘ a pregnant woman is not allowed to eat such things as pigs, rabbits, and ducks those are the things she is not allowed to eat. Tthe pig is forbidden, the pig, no..there is a religion that says you shouldn’t eat pork, for example: The Adventist, they don't accept pork, also a duck because it has legs that aren't widened and it's not allowed, and a rabbit is also not allowed. I am an Adventist’*

*‘ I think that as a pregnant mother, we often meet with communityhealth workers and I understand that it may not be forbidden because of a person's belief, but we crave something to eat because of the child in our womb is the one who tells us what to eat, and what not to eat’.*

*‘ I also understand that a pregnant mother is not allowed to eat medicine, herbal medicine, like when you feel unwell in your body, you run away to get this herbal medicine and you take all the medicine you see. I think it is not allowed for a pregnant woman, because it would cause stress to the baby and cause it to be prematurely born and have a bad impact on the pregnant mother’*

*‘ Islam has its own beliefs, because in Islam it is not allowed to eat pork, Islam does not allow a pregnant mother to eat pork, because their religion forbids it, and they say it is a sin’.*

*‘. Something we rely on when we are going to give our children breast milk, there are times when a child who is breastfed and It is not enough. You notice the child being greedy and the breastmilk only is not enough to satisfy the child, that makes us immediately look for help to satisfy them, to ease their hunger so that they continue to prosper’.*

*‘regarding the time to give the baby breast milk, when the baby reaches about six months, the breastmilk is not enough for him, so that's why we find him things like fruits, milk, we train him so that the body can accept food until he has grown and can stop to breastfed when he's two years old and he's grown up’.*

*‘ you asked us how often a baby should be breastfed, we can not count how often the baby is hungry and the mother gives him the breastmilk, the baby cries and the mother gives him the breast. If the child feels hungry, you give him the breast, then the other thing is that when the child is six months old, you give him the breastmilk and you see the child crying or you are eating something and you see the child eyeing it, salivating over it, at that time the child wants something else. give him the required complementary feeding like porridge maybe little soft foods so that the child can grow up more thank you’.*

*‘ As we see it at six months the baby starts to grow more, even if the breastmilk is not satisfying and we are looking for a way to support it, so we immediately seek the required complementary feeding and give him the porridge or the milk for those who can afford it, the lightest food because the baby has not yet started to have a strong stomach. we grind everything and give him light food’*

*‘six months is the right time for a baby to be breastfed without anything else’.*

*‘ the kind of complementary food we give to our children, we give them milk, porridge and fruit. The complementary food we are not allowed to give them are solid food since the children are still small at six months and their stomachs cannot grind like ours that's why we give them these porridges and give them these biscuits or cookies mixed into the porridge we have made to make it soft or light so that their stomachs can digest these foods without problem*

*‘ Thank you, why do we have to choose what is acceptable for our children, for example, a six-month-old child has a soft stomach, and you give the child that juice maybe you filtered it first, that porridge even small fish you grind them before adding them in the soft meal you are preparing, even those vegetables make them soft when you’re cooking them because the baby has a weak stomach which cannot digest strong food. if you give them hard stuff like cassava their stomach won't be able to digest it. So things are not allowed to take are big things like when a child is small, like cucumbers you can't give to a child, you can't give sweet potatoes to a child, but first you do simple things for him like make soft food as the stomach expands and expands as the child also grows step by step.*

*‘ the food that the baby should eat after he is six months old, that is to say, the baby should take a potato, green vegetables and put in a fish that you have previously removed the thorns for those who have blender can grind them but if you don't have it, you can prepare a good pot and cook the baby food and put beans in it, and then put it in tomato then you mash them with a fork when he eats it will be easy for the child's stomach to be able to digest it. Then for those who can afford it give the child milk and even an egg, often the child can eat five times a day due to the availability of food , if iit is available the child must be fed in the morning and drink the porridge, when you come to feed him again for lunch, the same meal full of all nutrients that you made for him. The child sleeps again and eats again when he wakes up because his body has rested. The porridge can not be forgotten even we can not forget to prepare the food with cleanliness with clean materials. Also give the child some fruits’*

*“ when feeding the baby after 6 months, as I do it, I cook it with potatoes, bananas, sweet potatoes, carrots, small fishes and cassava, and I add vegetables and rice because before I gave birth they taught us, we went to the village kitchen in a program by Gikuriro so when I cook for the child, I do it according to what I saw in the village kitchen, that it is the complete food that we should give the child, so the number of times I want my child to be fed is four time a day’*

*‘ his first year, as I did, I started to help him, I first gave him fruit and then gave him milk but when I saw he was having a big appetite, I started to cook food like banana, spinach and rice. This is what I could afford and I gave it to him, so I went on doing it’*

*‘ I understand the question but I don’t know anything about that’*

*‘ You asked us about food prohibited to give to a child in his first year, according to me they stopped me to give him internal organs meats because since the child is learning to speak if he or she consume them, they won’t speak. So it is prohibited, and the other mothers would advise me and they said ” you didn't give it to him meat from internal organs?" but I gave it to him indeed he did not speak he is slow to speak That therefore I learned it is prohibited in the first year of the child.*

*‘ for me when the food is ready I feed children first I prepare for them , then their father too I prepare for him then will go table however I like to first feed the children because children when they see that food is ready you see as they salivate over it, they want to eat there is no other choice than to feed them first. Thank you*

*‘ Me how I do it, first I put other side the meal of the chief since he went to provide for us so that the children do not play with it or spoil it then I feed the children. That is to avoid any misunderstanding about spoilt food of the chief.*

*‘ Thank you at my home how I do it, when the food is ready I put aside the portion of my husband so that I serve the kids and feed them without fear of spoiling the husband’s food that is my way of serving food*

*‘ Thank you, my husband he doesn't need food to grow up however my children do, so it’s on that belief that i serve them first and feed them on their plates then after i prepare the food for my husband that i put aside. You can tell him to be patient while you feed the little ones, he is a grown man he can’t fall asleep before eating but you can not tell child to be patient they are usually hungry by the time the food is ready they are also sleepy which is why i feed them first then after we eat me and my husband*

*‘ when I have prepared the meals, i serve the children on the plate and feed them after I immediately prepare another pot I put it in food of my husband and put it on the side so first fend for the children because someone older can be patient while the children ca not. Yeah first I care for children they eat until they are satisfied then i join my husband and we eat this also avoid a situation where we can eat and leave little to satisfy the kids. So i put my kids first then we eat after. Thank you*

*‘ when I was pregnant I needed those cares of eating on time even the children need that in that case a full meal I prepare us for me and the children, we eat togethwhile I prepare for my husband his own food.*

*‘ when I was pregnant, I got hungry very frequently and most of the time children were at school so I cooked for myself what I ate then I would cook what the children will eat even my husband, i would put aside their food in two different required boxes however at that time I was putting myself first since I was always hungry.*

*‘ For people who are pregnant for example before we had children you asked us when we were pregnant before having kids, what were the hours to eat? How were you eating? Me while pregnant there was no hour for eating , if I woke up I eat anytime I felt like eating I did that. however if my husband is home maybe he did not work that day I would cook and serve on the hours we usually eat at, then we share the meal so after we had a family I would cook and put aside his part then join him on the table to eat.*

*“ what I like to give my children among animal products is the driers all fishes sometimes eggs but I don’t cook meat that often since my husband does eat them.*

*‘ I love to give my child milk , eggs if possible I give him fresh fish or small dried fishes’*

*‘ food from animals products I like to give to my children are small dried fishes since they are mostly available, also I boil eggs for them and when I can I cook meat.*

*‘ among animal products I get for my kids there are eggs but I don’t surpass one egg per day, there are milk, fresh fish and small dried fish’.*

*‘ from animal products what I give my children I have money I buy meat, I boil them I don’t like to give them fried meat. I boil them and give them that soup and that soft meat, also sometimes I buy fresh fish but most of the time I get them small dried fish since it’s all I can afford regularly’.*

*‘, animal products I usually get for my kids are milk, eggs and sometimes fish or meat but I mostly get small dried fish for them’*

*‘ I do not forget when i’m cooking it’s the small dried fishes since they are good to the child, everytime I cook for him I put them into the meal also because we can easily afford them, at each little money you can get some at 100 RWF or 200 RWF so there is no way anyone can’t afford them, also I give my child milk and sometimes when God has been good which is rare I afford the meat and i give them the soup’.*

*‘ the food we like feeding our children like rice which have a quality because it was cultivated in the ground having not passed through the industry that is reason we love cook while we mix it up with many things and feed it to children’.*

*‘ quality of food intended for children includes vegetables , of course vegetables too have quality, there is banana in short plants cultivated in ground almost all of them have high quality there is potatoes , of course Cassava , of course sweet potatoes that that's the food with quality we give to children’*

*‘ The quality in preparation of food we feed our children includes washing up thouroughly vegetables even the small dried fishes. Quality includes to do it with cleanliness and clean them very well, cook them in clean utensils even serve the food on clean materials, that's it’*

*‘ I can prepare those vegetables and prepare the sweet potatoes cassava or rice if I have it then cook it with cleanliness. Just putting together a full meal which is clean is what I do for my children. I meant is to clean the vegetables with water, cook them with clean materials’*

*‘I take that vegetables and take maybe spaghetti then I put into that green vegetables and small dried fishes (fishlets) and if i happen to find some eggs that’s great , the food is of full quality’*

*‘ Macaroni yes i can afford it even green vegetables maybe the egg it’s not very often but i try once in a while’*

*‘ The quality in the food that i have to feed my child, I have to avoid putting oil while I’m cooking, if I have those vegetables I will wash them well, cut them then put them in that rice or in sweet potatoes or cassava also add beans and tomatoes, many spices are not good but the onions can’t be forgotten and I boil that. I just put a little oil but I can’t fry anything to protect my child then I let the food boil so that the food is well cooked, I learned that fried food to a little child is not good, the required nutrients are lost in that frying part which is why I boil the food’*

*‘ depending on who you married and the circumstances you married into you must respect your partner so in my home I give the thigh to my husband and the remaining to the children and me’*

*‘ When we have served meat, my husband comes first he must be dhiwn the redpect so he rake the bigger part then my kids can get some akso feeding a little child meat is not good what is important is the soup which contain the main nutrients’*

*‘ The soup is good it containsall nutrients, when you have cooked meat even eating them by those soft teeth is bad for the kid, when tye meat us cooked right, the nutrients stay in the soup’*

*‘ when I have cooked meat, I remove the soup for the kids first, you asked who eat the bigger slice or the thigh me I remove the portion of my husband then I give the kids the soft meat. The husbandcomes before the children. yes and I come lastly’*

*‘ when I am pregnant I love to eat meat so I eat first after feeling satisfied I then serve others’*

*‘ I first of all serve my husband since he is the one who went working so he must regain his energy, I mostly give my child the soup, I definitely don’t have to to give him meat because the nutrients are found in the soup’*

*‘ in our home we are equal, we don’t go into bigger slice goes to who or what, if I have cooked meat I serve we eat together whoever pick the thigh eat it but the husband can not say that he only get the bigger portion or me serving him that. You sat what you pick when serving yourself’*

*‘ In general I must feed him or her in the morning I give the child porridge then I cook what they will eat, I bathe them after when the food is ready I feed them then they go rest, when the child is sick I take a good care of him or her, for example if I have money I buy fresh fish so that there is soup and the child eat well there are times when the child can’t eat even can’t swallow the vegetables so when you have money you buy something else like the fish and you try to help him or her to heal’*

*‘ when the child is sick younmust take him or her to the community health workers because they have been trained in that, they test the child if it’s necessary they transfer me to the health center, if it’s a disease the CHW can cure, they give me medication, I make sure my child is taking the medication on time, I feed him, i observe him in order to help him heal quickly’*

*‘ I make sure they bathe, I feed them on time, but when the child is sick, when there are signs of a disease I live near the community health workers that’s where I take them immediately if it’s a disease the CHW can cure I get medication from there. If not I get transferred to the health center’*

*‘ on my part when I woke up I clean the house, I clean the basics of kids like brushing teeth, I give them porridge if some will go to school I take them for those who stay home I keep an eye on them while I cook so that for the lunch meal the child from school will find it ready. After taking lunch I put them to sleep after that rest when they woke up I give them porridge while I’m preparing dinner. So when the child is sick I do everyone time if it’s eating or bathing I even cook soft food because I can not feed the chicken child solid food like cassava, if I can afford it I get some fish and prepare a soup and some rice, I get the fruits. I get the juice some are affordable but in all I keep taking care of the child until she or he is healed’*

*‘ at my house when one child is sick it’s important that I keep an eye on him or her because when what I cooked the child can’t eat that, I look for what the child can eat, when the child can speak, he or she voices what she or he wants if it’s rice or whatever she or he wants, I make sure I feed that child so that in what he is suffering hunger can not be included’.*

*‘ there is the time I was pregnant with this kid I was sick, my husband is not always around but he used to come home bringing some meat for making a soup despite the fact that he doesn’t eat meat he cooked them for me but when I’m not sick, he wake up and leave no concern, he only help when he notice that I can’t anymore’*

*‘ Me when I was pregnant, I was so energetic that my husband could leave to work for days and I stay alone and I took care of myself since he was out there working to provide for us, so it was all me’*

*‘ So sometimes my child come up with a fever but there is no parent who does have paracetamol at home, when the child has a fever I bathe him or her in cold water then give him or her a pill before I take the child to the community health workers for them to take some tests, because the fever is bad you can get a little bit late and you could lose your child but when you have a paracetamol you give it to the child and attempt to stop the fever from rising’.*

*‘ my child can have a fever while I don’t have that paracetamol pill and the fever is rising up, there is a way I use, inlook for a clean cloth like a towel I put it in cold water then I put the soaked towel in the child so that the fever goes down the in the morning I take my child to see the doctor’*

*‘ in our village there are community health workers if it’s my belief I don’t know, I think that when you give the kid a pill and take that child to the health center they do not see the disease so when my kid is sick I take him or her immediately to the community health workers, they test my child to see if it’s not malaria if it’s not diarrhoea or runny noise because that’s what get the fever rising, the community health workers do the basic aid’.*

*‘ I lived in the countryside most of the times, the health facilities were far from home it’s not the same as in township, so the nurse told me that if my child has a fever take a clean cloth soak it into cold water put it on the head the fever goes down’.*

*‘ the fact of giving my child a paracetamol pill when he or she has a high fever, it’s a nurse who told me that. For us with little children they get sick frequently many different diseases so it’s good if you get paracetamol it costs 100 and you have it at home if I don’t have it me too I use the tactic of a soaked cloth to minimise the fever’*

*‘ Water to drink and water to use at home we do not have sufficient access because most of us we don’t have water facilities in our home, we travel to fetch water and when it’s not possible we buy water so if the water is not enough you get indecisive on how to use it if you’re going to boil water for drinking if you’re going to clean the house do dishes so we see what we can what is important and it’s what I use water on. So this make it that most of time we drink tap water without boiling it first’*

*‘ Like us we live in Murambi , here in Murambi there is a problem of water going missing, water comes once a week so the remaining days we fetch water from the watertank and it’s expensive since it’s 200 frw per jerrycan when water has been gone for long or 100 frw so buying sufficient water is not an option since we can’t afford it everytime so most off the time we go without clean water to drink*

*‘ where I live there are watertanks of the village even if it’s not near the house we try to go fetch water there because there is nowhere else we can find water unless we go to the valley which way too far’*

*‘ at home it’s my duty to fetch water unless my husband is in a lovely mood and he does it for me. When the water is gone in the area where do almost 2 hours travel just to fetch water while climbing the hills and it’s so down in the valley that we are required to jump. So I go fetch water with the child on my back and a jerrycan it’s hard, it weakens us, upon arriving home I’m often breathing badly and chest hurting’*

*‘ you just asked us who fetch water at home, at my house it5my responsibility maybe when I have a maid ither responsibility or we go together but my husband can’t go fetch water even when I’m pregnant he pay vor someone to bring water at home so that we don’t drink water from the tank because they don’t get cleaned regularly not even once a week... but only once a year. Water from those tanks are a problem since they mostly have little worms you can not use it at home to cook’*

*‘ the consequences we meet while we go fetch water or when the water is missing in the area, at my house no one is charge of fetching the water, whoever is available does that because we all use that water or we go together when it’s possible but when we have little water we choose what ro do with it.. Some things remain a mess and they wait until the water comes back especially when in the village tank the water has finished too, the price of buying water goes up to 500 frw per jerrycan so that is one of the challenge we meet’*

*‘when my husband is around he helps but when he is not, I do that and everything else since wherever he is he is working too’.*

*‘ sometimes I tell him I will take care of the kids, hehas to go fetch water we will be using. He does that without issue but I can’t get used to that, I don’t that a lot we just compromise sometimes’.*

*‘ we have toilets but one of the issue we meetbis that like me we live in a house we rent, also other houses we have only one toilet we all use, we have kids are in primary who are starting fo use the toilet so I always clean that toilet for the sake of my kids and the neighbours dirty it or we get infected by someone else. So only people who are rich have their own toilet as for us we use one toilet for 6 households almost 20 people per toilet’*

*‘ About the toilet we share with people who live close to us since we live in a house we rent, my children are under three years old they don’t use that toilet I have the designated toilet for them that they use and after I clean it. I can’t allow my kids to use that toilet at such a young age my children can get infections from there. After my kids use their plastic toilet I wash there hands and clean them, this goes to show that the art of washing hand is a thing we do at my house even guests practice that since there is a designated can of water to e used after coming from the toilet’.*

*‘ me my kid has a designated material(potty) used as a toilet, after finishing to poo I clean that material then clean the kid too, it’s hard to protect girls from some microbes they might get from toilets when it’s not properly cleaned so to avoid that since we share the toilet with others we each bring water when we are going to use the toilet so that we clean after using it’*

*‘ where I live it’s a rented house I use a regular toilet I have other ,kids who use it, the challenge we meet is that some people we share it with have dirtied it, if you don’t clean it, you can get sick so we put in a jerrycan to be used while cleaning when youngind it dirty. After using the toilet yes we wash our hands we don’t have the step and wash but we wash hands with water and soap normally’*

*‘ you just said about the step and wash and it’s not possible with where we live, I think where it can last it’s at schools and at health centers but as in our home if you leave it there at the toilet it won’t last, kids destroy that quickly we are overpopulated here, even soap can be stolen in no time, like how my house is located everyone pass by, if I was living by myself in a closed home I could take care of that I really tried where I live putting the step and wash then a bucket and a soap but it got stolen. So now we use water and a soap normally’*

*‘ challenges we face because of water in cleaning, when there is no water, you can not clean well in your home it’s in that case you will find a dirty place, dirty people or you find children having skin diseases because they are not being washed thouroughly’*

*‘ challenges we face mostly when there is no water in the area I can’t do laundry or later in the day I haven’t cleaned the house or dishes, but if the water is available I do everything on time and I clean well, it is always dirty in my house when there is no water ‘*

*‘ As I understood you asked about the challenges we meet about water, cleaning , so when there is little amount of water nothing get done well if I was supposed to clean the vegetables thrice I will clean them once everything is being done quicly5with little amount of water which can guarantee the cleanliness until water is available again in an abundance’*

*‘ challenges we face because of water is our children having diarrhoea because of dirty water we use’*

*‘ What can be done is that they should give us access to water so that a mother can clean well since water play an essential part in our daily life so if we could get quick access to water things will be well’*

*‘ me I think if you could ask for us that each household has watertap or maybe watertap nearby in between houses so that we have sufficient water easily since where we rent houses we don’t have tap water’*

*‘ What I think in order to improve the life of a pregnwoman and a child there should be a time where there are discussions because everytime we need advises there are things we can not fix by ourselves so if we are to discuss even community health workers to speak with mothers that would be good’*

*‘ there should be a sensibilization for pregnant women how to treat their kids or stimulate them to take care of themselves, even discussions among lactating mother on how to properly feed their children’*

*‘ Like for ladies that are pregnant should be encouraged to eat a full meal, to stay clean for those with kids to keep an eye on their kids in order to help them when needed so that they grow well’*

*‘ a mother can say that she will take care of herself but she has no means to do so you find the kid doesn’t well, he doesn’t more than once sometimes it’s not because the mother doesn’t care but she has no means, I think if there was a financial help it can help some parents. If there is what to eat regularly a kid can’t get bad.. he will grow up well’*

*‘,when at home you are poor, nothing goes well, nobody wants the children to grow bad but the means to afford that is the problem. If there could be any financial help it could help in improving the life of our children’*

*‘ when at home I can afford what to feed my kids, and what to drink there could be no conflict, it happens when there is nothing and your kid is about to go a day without eating, the mood become tense, parents argue but when there is food there is no conflicts’*

*‘ Everyone has a way she manage the household I can’t say for others but what I can say is that a housewives doesn’t have what to sufficiently feed their children, there is always a fight among partner’s*

*‘ in my household it’s not how it is but somewhere it happens spouses arguing, threatening each other depending on who provide for the house, the consequences comes to the child, those sort of conflict exist in our village’*

*‘ conflicts because of financial situation exists, somewhere i know they failed to pay the house rent for 5 months they got evicted, they started threatening each other, all becu5of poverty so we all want the best like I want a kitchen garden but I can’t have that because it closed off where I live so any help would be good’*

*‘, between a husband and a wife when there is food all is well. When there is nothing and the husband leave without leaving enough money to buy essentials, she get sad even though she tries to manage but the child ends having low weight, yellow in MUAC. So sometimes we can’t afford all the things needed to keep a kid healthy so financial help would be great*

*‘ I think those conflicts come because you haven5talked well, if that little moneybis what the husband has, if there are no secrets there can not be any conflicts but if the husband is hiding something of course there are conflicts’*

*‘ Among the foods that are grasses and animal products, what the mother is not allowed to eat are leaves that are inedible but she is allowed to take edible leaves as they are also among vegetables, she is allowed animal products because there is milk, and meat that the pregnant mother must eat, and then there are plants that build up the body, there are things that stimulate and there are things that prevent diseases. It's all plants that a pregnant mother is allowed to eat, ‘*

*‘, I think the foods that a pregnant mother should not eat are the foods that could kill her, and everything else that does not affect the body she should eat it, whether it comes from animals she can eat it because it is useful, whether it comes from things greens she should eat as it good for both her body and the baby, thank you very much’.*

*‘ for example, maybe like bitter cassava that can cause unplanned abortion, because it can cause a problem inside the womb and at the end the mother can she might lose the baby that she was preparing to have, the child she wanted. And for what is allowed she should them because every food have it role in the body’.*

*‘ the plants that the mother is allowed to eat are the plants that build the body, actually everything that builds the body like: beans, gran nuts, sunflower seeds, and seed in general. Either that or animal products, it's great that she's going to eat animal products. Foods that she should not eat I don’t know much about that for sure, except for foods that are plant-based, which are animal-based. It is good for a pregnant or breastfeeding mother to take it because it prevents the baby from getting stunted while still in the womb or after birth. Therefore, it is better to take animal products because they protect the baby from growth delay during pregnancy. Regarding greens except for herbs that are not prescribe by a doctor and act like drugs but she is allowed to eat greens as lot of vegetables are greens and it helps for the child growth’*

*‘ green vegetables a pregnant mother is allowed to eat are amaranth’.*

*‘ it’s not only a pregnant mother who should eat amaranths but also us mother with children, vegetables are among the things that make us have a better sight (eye vision), otherwise it works well feeding a child amaranths, if you are able to get dried fish let and add their flour to the amaranths it becomes useful food for the child’.*

*‘ there is something else I would like to add, among the things we know that the mother is not allowed to eat such as smoking tobacco or cigar as we know they come from plans pregnant or lactating mother is not allowed to smoke, and drinking strong alcoholic beverages. A pregnant mother is not allowed to smoke or drink alcohol’.*

*‘ the reason why the mother is not allowed to smoke, is because the smoke goes into the lungs and then reaches the child as we know the food and drink the mother eats and drinks during pregnancy feeds her and the child, and that can cause the child growth delay. Tobacco and cigar can cause child stunting during pregnancy. Also as the mother feeds her and the baby drinking alcohol means the child’s blood will be full of alcohol and at the end it affects the well development of the child’s brain during’.*

*‘ she is allowed to eat vegetables, cassava, fishlet or small fish, energetic food the things she is not allowed to eat are exactly what they said, alcohol and drugs’*

*‘ depending on the religion , regarding food belief, for example if I say I’m Muslim and I'm not allowed to eat pork I will just don’t eat it, thank you’*

*‘ according to religious beliefs, things that one is not allowed to eat, for example: for Adventists of the seventh day, anything to do with meat is not allowed, and that's what I'm talking about’*

*‘ often things that are not allowed are alcohol and tobacco’*

*‘ pregnant mothers should be taken care of by eating a balanced diet, which includes three types of food: body-building, energy-boosting and disease-preventing foods. And eat three times a day, with two small meals in between. A small meal is porridge and fruit, and then so that she can be healthy because when a mother is pregnant, she is also nurturing the one in the womb and that will make the child healthy and turn will be born with the right weight, it will protect the child from growth delay while he/she is still in the womb, because growth delay starts from the time a child is still the womb they say. So if a mother eats a healthy diet and eats three times a day, eats two small meals, and avoids the distractions of tobacco and alcohol, the mother will give birth to a healthy child with a healthy body’*

*‘ A pregnant mother needs to be taken care of she has to take care of two people, she has to take care of the mother and the child, and the child should be taken care of since fertilisation . The reason why the child should be taken care of since fertilisation is that he will be born healthy, when the mother is not taken care of while pregnant the child does not. Well. he is not happy and the pregnant woman should not overwork herself, she should not be told wrongly, she should be taken care of so that the child will be born happily and will be born with a healthy weight, protecting the child from growth delay from the time of fertilisation until the child is born in one thousand (1000) days they teach us’*

*‘ the effect on a mother who is not taken care of when she is pregnant, the child is not born happy, it makes the child feel that if his parents used to fight, the child is also born feeling unhappy, you see there is a mother who gives birth to a child and told me: "I have a child who is three (3) months he isn’t laughing”. It often depends on the behaviour of the parents who are pregnant with the child, so the consequences if the pregnant mother is not taken care of affect the mother and affect the child, because there are many symptoms you can see due to the fact that the mother is still pregnant’*

*‘ the other thing I can add is that when a pregnant woman lacks the security what she feeds the child is what she missed so she is not safe and gives the child less security, like when she is isolated, the child in the womb may refuse to play, it will require more effort for the child to wake up and play again, when he would start to play and the mother lacks security again the child is going to be alone again, and when comes the time to speak you will see that the child likes to cry he does not smile or with other children even if he grows he is unhappy, the reasons for all of that are what her mother fed her when she was pregnant and what grew in her and what she gave him because that is what she saw so in few words the pregnant mother should be given complete security’.*

*‘ safety is important because in stimulating the child's brain the first thing is safety, the mother should always be happy. Even if the mother feeds him well but lacks security at home, these things will not help her much, so security is important for the mother to see how to stimulate the child's brain. As my friend said, when the mother is not safe, the womb stops because when the child is in the womb, he hears what makes the mother happy or unhappy, that's why the pregnant mother must be safe it also affects the child’*

*‘ regarding breastfeeding the child, he should be breastfed eight (8) times a day but it can be more than that’*

*‘ the kind Complementary feeding, you can give a child is like a porridge or fruit’*

*‘ actually a child should be breastfed since birth because that yellowish growth delay. The child must be breastfed eight (8) times a day or seven (7), the child must be breastfed for six months (6) straight without giving him/her anything else, after those six months you can start giving him/her things like fruits, porridge that is filtered due to the fact that their stomach can’t handle solid things yet, as the child grows, you change his diet and give him breast milk until he turns two years old, but otherwise the child will have to breastfeed often during the day and night so that they grow well, because the breast milk contain substances that protects them against many diseases’*

*‘another thing I would like add to what my colleagues said is that, the baby must be breastfed as often as possible let say up to eight times a day, but when he needs to take complementary feeding, you should give him a diet that is full of nutrients, energy provider, and disease prevention. A parent must remember to smash the food until it soft to start feeding him and as he grows you add more food and you can give him solid food as he grow also give him fruit’.*

*‘ In the past parents use to give their children alcohol and when it time for complementary foods they continue giving them alcohol instead of porridge. When a child is six months old breastmilk only is not enough but giving them alcohol lead to stunting, it should not be included among complementary feeding or giving them boiled cassava only or maybe grilled potatoes, actually it not prohibited to eat that cassava potatoes but it good to add other kind of food to them because children should take balanced diet other than that it not really advised because it does not contain nutrients on its own, it must have something added but better add fruits and vegetables to their food for their well growth’.*

*‘ the forbidden things to give a child is the alcohol, and to give them the sweet potatoes because they also cause diarrhoea. It better to get them some good foodstuffs mixed with vegetables, dry fishlet’.*

*‘ In the first year, the food a child should eat is simple food, which includes: vegetables, small fishes, and the porridge that we mentioned have been filtered well because his stomach is not yet able to process hard food, thing they can eat easily for example, a smashed green banana which is sweet and soft so that it reaches the stomach and goes well not strong enough for them to have any other side effects, but green vegetables especially amaranths or spinach and small fishes are fine’*

*‘ firstly you the baby should be given among fruits bananas, other food like biscuits, and some plums’.*

*‘ a child should eat three times a day but mix it with porridge so that he can be fully satisfied’.*

*‘, usually for the drinks that we give to child around here, for example, if someone has money, you can buy a half-litter of milk with a hundred (100 Frw) and mix it with a porridge and give it to your child’*

*‘even if you don't have milk, you add some more soy, you know they often contain milk of their own, thank you.*

*‘ from animals product generally we have to feed the children milk, fish, meat but we don't get all of that here often, when we get some money we look for small fishes, they are often the ones we like to get because they are cheap, but like eggs and meat they are not available very much otherwise it is necessary for the child to take them but we don't have money for them’.*

*‘ Yes Gira Inka did get to some people, but in terms of milk it not available well, even if this mother right here said a half-litter at 100 Frw there are some places where they can’t accept that money and in time like this it difficult it 150 Frw because a littler is 300 rwa that is also why milk isn’t that available around here, and if one can by it usually they mix it in porridge for the children to drink. I can say that 40% of people here are the only ones that can get porridge mixed milk for the rest they just take it that way if the get it but we believe the actual porridge we give to the children contain enough nutrient; maize, soy, millet, sorghum and some kind of beans called corta, all of that mixed and grinded form a good source of food and nutrients which is a perfect home-cooked meal for the whole family’.*

*‘ Some had been given them, but usually it given to people in the first cast (Ubudehe category 1)’.*

*‘ in all the foods that we all take as food, there are no foods that are forbidden to be given to the child, but it needs to be properly prepared and cooked because there are foods that build the body like beans, there are energy-boosting sweet potatoes, cassava, and vegetables that prevent diseases, they are all foods. But there is nothing wrong with giving them a child, the worst thing to do is to give it to him when it is not well prepared, that's my opinion’.*

*‘ you shouldn’t feed a child who is less than a year old with a hard cassava only because it will harden in their stomach and cause some complications, you should give them simple and soft foods: green bananas, cassava paste and soup or rice’.*

*‘ some foods that are forbidden to give to a one-year-old child because they don’t have full teeth like a human, if you give them meat they can't chew it and in return it can cause trouble’.*

*‘ At our house, you first prepare it make sure it ready to be eaten and feed it to your family members, that's how I think it done’.*

*‘ the quality of that food would have and that we should pay attention to is that you can’t just take a plate you ate on yesterday and use it again today, first you take and soap and wash it and put it in clean water maybe twice and then for us who are poor and don’t have dish-wipes you look for somewhere so the plates and spoons (dish) can dry up, and then from there you can actually put food on the table’.*

*‘ we wash our hands and wash the dishes’.*

*‘, how I understand about quality is that we have to have a cleanliness like if you are going to cook a banana, somehow it gets dirty If you are going to cook it, you should clean it. The vegetables you are going to cook do it with cleanliness carrots you have to peel it then clean it. Wash the dishes you will use and hang them on the bed to dry and then the sun will kill the microbes that are there when the dishes are dry. Boil the water and clean its container, every plate, bowl has to be clean in such a way that the person who goes to use it feels like eating because of the cleanliness you have done. We must prepare well the food we eat but the first and important is hygiene’.*

*‘ Another thing I would like to add not further from the truth of others. You see that our work is often farming due to how someone comes home tired and how someone says let me eat quickly because we are hungry but that is not acceptable because what we are eating comes from soil contaminated so are our hands. We have to prepare the food with cleanliness and then cook them carefully since in the water we have used sometimes there are microbes that will die and the food is of good quality’*

*‘ often when we are at home, the pregnant woman is at home, the husband is at home with the children, I stand my ground and my husband comes last. I put aside his food and he eats it later’*

*‘ Yes, he mostly arrived while I was done and the meal I prepared for him is on the table’.*

*‘ yes, when a woman is pregnant, she is hungry! But if the children are there, you can't eat twice when the child is looking at you, you immediately put some food on the plate, you don't rush him, you give him water first and wash his hands. Then he eats’*

*‘, when I prepare a meal when the boss is not there, I would not say that I wait for him. You cannot know the husband’s business; you can’t know at which hour he will show up so when the food is ready we put aside his food then we eat but when he comes you have to keep him company and eat a little’*

*‘, you can’t say that you wait for the husband and you put aside put aside his food when he come you keep him company, even if you eat one spoon it will be enough. What I mean is that I put aside the portion of food for my husband so that I serve the kids and feed them without fear of spoiling the husband’s food that is my way of serving food’*

*‘now there are beans, potatoes, bananas, sweet potatoes, vegetables and these days there is hunger even now beans are hard to find but we are trying to get them to support our households’*

*‘ The food that is available these days is only a few beans, potatoes are also few, considering everything is in small quantity but it is available and we can have meals. So when I prepare my meal on the table, I would first distribute the children's food and then I would feed others and eat it lastly. We are used to have meals at the same time. If the husband was not there, we would put them aside for him’*

*‘ Quality first is to clean it first, as my colleagues said, the dishes are washed early and dry and before serving the meal, first wash with water and soap with the children even if they use spoons, then we can go to eat, thank you’*

*‘ (laughing) the biggest piece of meat is for the husband.Because he is the head of the house’*

*‘ The bigger part of the meat is taken by the man because the man is the one who bought the meat and if he buys it then comes and find you put aside little there can be some conflicts. So we tread carefully yes we eat the meat but the husband gets the big part without lying we have that in common then again each one of us have the way we manage our home’*

*‘ I would not go far from what the others said and the husband get the bigger part then me. So yes the husband takes the bigger part’*

*‘ what my others are saying is the same because the man is the one who is the one who provide. He comes first then I follow’*

*‘ Well we share but you have to put the feeling to make him pride himself that he provided. Yes, while you are the one who cooking you nibble, so you have to prepare a big meal for him, then the others will follow’.*

*‘ I am number 5 and at home, when the man has bought the meat, after preparing I serve each one of us even the kid.my husband sometimes cut and gives the child on what I served him so that the child be happy that father fed him. So I serve him the bigger part then he adds to what I have the kid’*

*‘ I am number 7 that the biggest meat should be eaten by a man and that is it’*

*‘ Yes, how I understand it when you are serving the bigger part goes to him because he is the provider if you will eat two pieces he will eat three that’s it’.*

*‘, you gave us an example of chicken. They say that there is a piece of meat if you eat it and the husband come you serve him other pieces and he doesn’t see it no lying pack your things and go back at your family no discussion if that piece he didn’t see and eat it, conflicts are there’.*

*‘ If you don't give that to him (laughs) you will be divorced’*

*‘, I am number 5. For me that issue of that meat piece it’s like myth because when you buy he sees it that piece isn’t even half of a chicken thigh or wings, don’t see the problem there what if you put meat in a bowl and the kid serve himself on that piece? You would refuse your kid the meat? I don’t see the issue there (laughs) Thank you!*

*‘ As far as I know, everyone has their mind, even how you manage your household, it also depends on what your husband is used to, we cannot say that all husbands act that way because some don’t but for me when I still had him he is dead now but when he brought meat he didn’t care that he eats this piece or that, he ate what I served him even on chicken any piece he ate it with pleasure either chicken thigh or breast, he didn’t care on which piece I served the kids. There are some habits you get used to like some women who cannot cut the banana when the husband is around it depends on the habits of the person we cannot apply it to all men’.*

*‘ I am number 3, for the most part there are some homes where a husband and wife have kids they live together but the husband abuse the wife in time of eating especially these days which are bad there is little food the wife and the kids eat little in order to avoid angering the husband if he claims the food is little, those household exist. The children who are stunted because of spouses conflicts but these days there was some sensibilization some teachings and discussions among community health workers and a better understanding, the level of household’s conflicts reduced so yes because some husbands can be greedy, kids tend to eat little in order to obtain some level of peace’.*

*‘ in these days to find sufficient food for eating is a problem I don’t want to embarrass them but when it’s hard they free, if he has something he fix his problem then the mother with kids are left alone so the mother take charge and she provides little she can, soap, salt etc… and sometimes she can’t get it all so the lids get stunted because their father doesn’t support them and one support is never enough to raise kids’*

*‘. We focused on men as if they are the only ones with hunger, some mothers see that the husband is not supporting the house then when they cook they eat first when they are full they feed little to the kids, those are some cases where the child has not had enough then he start going to other homes neighbors in his mind he knows where they will give him food and the kids eat in three households which wouldn’t be necessary if the kid was satisfied at home’*

*‘, in general you take care of the child from the moment he or she wakes up by bathing him or her, if they go to school you send then and you talk to the kids ask what they want, if you can afford it you get it for them that’s what I do at home’*

*‘, The way to take care of the kid, their life are not like ours especially these days that there is hunger, since there is a small quantity of food you have to tread carefully in order to avoid the kid going without food so in the morning you give the child porridge if there is food give it to the child then after coming from the cultivating you give her or him porridge while you’re preparing the lunch then you feed him even in the evening, you try to feed your kid as much as you can it’s not like us that can be patient. Community health workers teach us and we do what we can about the full meal the kid must take’.*

*‘: Especially when my child is sick, when I see that he is sick, the first thing I do is to take the child to the doctor as for the food to feed him I try my best and I provide dried fishlet, vegetables amaranths since there are available then I try porridge, bananas and tree tomatoes so that the kid get energy’*

*‘ otherwise the little children to take care of them is now too expensive to get him dried fishlet, you put it in every pot, you won't find it every time, but you take a small potion for the child, if there is two bananas or three pieces you add that, If you include vegetables, the child will be well taken care of so that he/she can't have a problem since you avoided giving him food as everyone else which probably won’t have all the full nutrients. So you feed the child while nibbling to encourage him or her’*

*‘, I am number 5, when you see that a child is very sick, we have community health workers(CHW), you immediately go to them for a consultation, they examine him and they treat the child when they cannot treat him they transfer him at the health center to see if he is suffering from something else’*

*‘ When a child is sick, they take him to the health center for treatment’*

*‘ children often get sick and catch a fever. When I see that he has a fever, I take a clean cloth and soak it in water then put it on him to make sure that the fever reduces’.*

*‘ When I see that he has a high fever, I go to the community health workers and they give him pills if it doesn’t help then I take him to the doctor and that’s the help give my child’.*

*‘ I am number 5, as we live with our parents who raised us in the past, they used to do it even when I was older and you see him take water and a cloth and put it on his head when it hurts or there is a fever. That is how I learned it so when my child is sick that is what I do before going to the CHW’*

*‘ These things parents do of taking the kids to the to traditional healers (in traditional medicines) while we have community health workers everywhere is not right, they always tell us to go to the health facilities if children are affected, sometimes parents take their children to traditional healers for things like Eczema or for baby tooth( local language: Uburo & Ibyinyo ) regardless of the fact that they treat that at the health center, doctor usually tell them not to remove the baby tooth because may not grow back but people out there don’t understand. Me on the other hand have never done such thing with this child of mine, as people don’t understand all we can do is continue telling them the first to bring a sick child is to a health professional and not traditional healers’*

*‘ what I can add, actually we know that doctors can cure every diseases, so when a child is sick and you should immediately bring him to the doctor, the important thing is to take the child to the hospital early without waiting for him their condition to get worse and he will be treated and will be cured. I like taking care of the grandchildren at home they are really young kids who are but they don’t suffer from ibyinyo or uburo. It a few people that still go to traditional healers actually, most of them are now aware of the danger that it represents and they now also understand the importance of going to the doctors or community health workers first instead of traditional healer. I have never seen those illness they take to traditional healers actually, I have children they get sick and I take them to see health professional and they get better, nowadays I think authorities deals with those who pretend to be healers but still some still do it where they can’t be seen’.*

*‘ Thank you. First of all, if the child has a high fever, bath he / she then you send him to a CHW because they are the ones who understand what to do with the sick child. If they see that he has many symptoms, the CHW will look at it and immediately knows what to do after consultation if the CHW can’t help the child, they give you a transfer there is no more we do other than that you can’t keep a sick child even a fever it can result in death’.*

*‘ Our child had fever once and teeth were hurting we didn’t go to the CHW or Health center, we went to see a traditional healer and they treated our child, thank you’*

*‘when the child is taken by toothaches, he is not calm even when he is asleep, even if he is sleeping, he is not calm and he does not eat because he has pain in the teeth’*

*‘ we get water, we fetch water to water tank or tap water, and we boil it and put it in a clean pot, so that they can have clean drinking water and have a healthy life’*

*‘ we get clean water, even if you go to fetch it from the tap and call it good, but because of the jerry can we put in it, we don't trust it to be clean. We boil the water and sometimes not we just make sure that the jerry can that we are going to use is clean and dry, so that when children wants water you give him in a clean cup and he drinks, children want water so much I don't know why but we also drink it without any problem’.*

*‘ we thank God the government brought water near us, before we fetched water from water source but some of us who say they boil water before drinking it is like one percent or two percent of the people here in our community. We fetch water from the tap, but we have run out of food and we have run out of wood, so there is no place to save things’*

*‘ we get water, without lying, it's not always boiled, sometimes we drink it right away, sometimes we boil it, but we get water for drinking’*

*‘ where we get drinking water, we have water in the villages, we drink it from the taps not boiled, to boil it is a problem of little understanding but we all know that it the best thing to do. We know that that water even if it comes from taps the pipes water pass trough are often not clean and sometimes have rust so not boiling it is a bad decision; there are times when the tap water isn’t available so we have to fetch from swamps which contains microbes and not boiling it is when you find kids suffering from diarrhoea. It should be a common knowledge to boil the water till clean for consumption’*

*‘ the government gave us clean water in the village, but about boiling water I can’t lie, when I am lucky and get money, I go and collect it but can prioritize boiling water over food you also know that they days are tough but sometimes with the little money we buy water and put it in the house to drink, and when I want water to use for other activities we go and fetch it from the swamps and that is what we use’*

*‘ we buy water for use, now the jerry can costs thirty francs (30 Frw) but it used to be twenty-five (25 Frw) then the reason why is that when a person runs out of money like this, they go to fetch it in places like rivers or stamps, and they fetch and say that it is not for drinking and they say let me by some water from the tap maybe a pour fifteen litters (15L) and he goes and puts it in the house and that’s one to drinks, thank you’*

*‘I have ta toilet, it is built in wood and mud.: When a small child wants to go to the toilet, he goes like in front of the toilet he/she finishes I remove it and throw it in the toilet’*

*‘ I clean it myself and throw it down the toilet’*

*‘I also have a toilet at home, built of wood and mud as usual, and uses tins roofing sheets when they you can afford because as they say we should abolish houses build with grasses “twirukane nyakatsi, but the floor is mud’*

*‘ I have a toilet, built on the floor with wood on the sides and bricks. If you are lucky enough, you will find a cupboard that you put on as a door to protect it so that if someone passes they won’t see you, so when you get out of the toilet, you take water and wash your hands with soap. You can’t go and get things to cook without washing them first’.*

*‘ actually when a person comes out of the toilet, if you don't have step and wash you have a jar that you have prepared there, take a small soap and put it close to where the person who is out of the toilet will pass so that they can clean their hands. I have a child who is 3 years old when he leaves the toilet he says "mummy wash my hands, mummy wash my hands" so now I'm doing it for him but when I'm not there he pours it for himself but the toilet is there. So for me it is a jar I don’t have a step and wash’.*

*‘I prepared a jar myself there, because sometimes you do it and you find the children in the village threw it so I put a jar with water and a little soap’.*

*Interviewer 2: Thanks my friend for giving me time, I want to ask you that when it rains, you told us that children can't go to your toilet, when it rains and you haven’t removed the dirt, what happens? Doesn’t it happen to you that it rains and you haven’t removed the dirt that the child left on the side of the toilet?*

*‘ it usually doesn't happen unless you're not there, otherwise when you're there, the child finishes pooping you immediately remove it and throw it in the toilet, so when the rain falls when you're not there and you didn't clean it, like now we have vegetables and kitchen fields at home, then you have to take care of it so that it doesn't get in the vegetables, because if the dirt goes into the vegetables and you eat them, it is a problem, it takes a lot of washing but we take care of the dirt and remove it, we also have to make sure that the toilet is clean. we have to wake up in the morning and clean it, we don't use water and soap but you wake up in the morning and take the ashes from the kitchen and go and pour it in the toilet and then sweep it, so that there are no flies There are a lot of flies when they come out of the toilet and they fall on the vegetables or fall on the kitchen equipment you use leaving behind a lot of dirt. When it rains and the mother is not there to remove the dirt it when you find that people at home are sick with intestinal worms, as sometimes small children can go and touch the dirt and when you are not there to wash their hands well with soap and water and they go and eat without washing their hands later on they become infected and get diarrhoea, vomiting and other diseases caused by dirt, but otherwise parents have to take care of it and clean the place where the child pooped so that it doesn’t bring consequences’.*

*‘ The problem is that sometimes water is unavailable, sometimes in the morning water is available and then after a short time it is gone and it is difficult to get water in that situation what we do is fetch from swamps or people who can afford buying it go and buy it from other cell where they have water, and it is hard to clean in that situation. Thank you’*

*‘ Another problem that a person faces is that water is often needed at home, as you sometimes have little water at home, as they said when you go to fetch water at the tap and it is gone and you know there are dishes to be cleaned, kids needs to shower and you need to prepare things to cook and water it is unavailable that is when we go to fetch from swamps, and we don't trust the water from the swamps but we use it anyways. There are often obstacles, when there is no water. At home, we do not clean well, because when you fetch water from swamps you clean dishes it like doing nothing because the water is not clean, so obstacles are there. Thank you’*

*‘. I wouldn't go too far with what my friends said. When there is no water, life seems to have stopped. Because water is often important at home, so when we go get water in the village taps, first not all of us have the money to buy it. We buy drinking water, we said we buy it thirty francs (30 Frw), we don't even get those thirty francs sometimes. Instead, we go to water in the swamps and you will find that’s what we cooked with, that’s what we washed the dishes with, So that where we get disease’.*

*‘ challenges can’t miss including miles. When you go that far with a jerry can and by the way you will have to go back like three times so you can have enough water when you home and you get to go there yourself it tiring’.*

*‘ As she said, there are times when we don’t have water at village taps, like there is no water we fetch water very far in mountains and you find a mother taking children of four and five years old when water is running out to go and bring water, and the kids will go with those little jerry can of five litter first they come late and most of the water is spilled and at the time the parents don’t have any other choice than going there alone and sometimes the mother don’t have any energy to go. Thank you’*

*‘ Yes, because the child is still young. But sometimes the man of the house, when he sees that I have no time, he also helps me and brings the water. Because sometimes I’m busy doing house chores’*

*‘ When it comes to cleaning and sanitizing, you see that water is needed and soap is needed. Water can be available but now the thing called soap has become difficult to get. Often there are times when you can find water but soap is missing. If you don't have soap, you're not doing enough cleaning. Among the most difficult things to get is soap.’*

*‘ Soap is expensive because now you can buy a one hundred soap (100 Frw) for one wash It disappears immediately It problem. Finding soap is a very difficult for problem is us.’*

*‘ At this time the situation is so bad, then you see that there is also no the rain and there is no water in the taps. We send the children to the swamps to fetch and sometimes we go there ourselves, there are times when the child goes and you are left with fear. He may fall into it, he may meet criminals, the neighbour may be worried because is the near person. And somehow you got the water to use and the soap is missing. Which means one thing is available and the other is unavailable. Things are tough these days’ cleanliness and hygiene are getting tougher to reach these days’*

*‘ one thing that needs to be taken care of regarding pregnant women and small children is that we fill the need to ask that at least every household to have a small livestock. Because there is a need for meat, eggs, and milk, often those who do not have cows may a rabbit, have chickens, the chicken lays eggs, and those eggs are fed to children and pregnant women. Because it's not easy to find big livestock. But if at least every household have small animals, pregnant women and children can eat enough food, eat a balanced diet, and get more from animals, it would help us a lot to be able to have a healthy life’.*

*‘ if what she said can be done and the addition of more water tankers or taps for when ones are running out of water and the others have water, I think the lives of pregnant women and children will be better if all these are found. But more power into water supply.*

*‘ as for the pregnant women we talked about those full meals that they should eat and porridge, but that porridge, there are people who grow maize and we have problems selling what could add to what we have, that's how we won’t get that porridge’.*

*‘: Also, you see that the times we are in are bad, there is something they do at health facilities, it is a government program. Parents in the first Ubudehe category are given food by food I mean porridge flour to feed children and pregnant mothers. We also asked, those of other categories who do not have economy sufficiency to get these things, at least to have help as most of us in the second and third classes are poor. We requested that at least anyone from any category who is pregnant or has a child under five years of age, we request that kind of help so we could feed our children well. Because even though we are in those categories, we are not able to get adequate food and sometimes you find that someone from the first category is more able than you. Therefore, we requested that at least every pregnant woman from each class and a small child from each class, can access a flour of porridge, it can really help us and the children can live well because there are times when you need money to buy even sugar and you don’t have any’*

*‘like they said getting a full diet is difficult for us, like a pregnant woman with children under the age of five, a child can want porridge, and you have been looking for money for about a month, and when you get it, it is not even enough. And I say, if I buy him porridge how is he going to eat at lunch time and you prioritize? It is difficult for us, sincerely speaking to find the flour of porridge is hard for me, and I have a small child who needs to drink, we ask you to speak on our behalf. Because it is difficult’*

*‘ The pregnant woman is allowed to eat the vegetables, animal product, eating meat and fish, that’s it’*

*‘ a pregnant woman should eat foods that contain antioxidants, stimulants and body builders but a pregnant woman is not allowed to drink alcohol or smoke, yes, because smoking is harmful to the pregnant child’.*

*‘ How does it help the child? You see a pregnant mother is feeding the child in her womb because what the woman eats also affects her child’*

*‘ A pregnant woman who eats body building and stimulating foods is for the child to have immunity due to the condition of the child in the stomach, then when she eats animal products that build the body very much so that the child will not be born with a problem’*

*‘ it helps her to have a healthy life as if the child she gives birth to is a complete child’*

*‘ Another thing I would like to say is that for all that they said, add fruits, because we can find such fruits in the prevention of diseases ,we can mention common fruits like bananas, almonds, apples, nuts, etc. so for a pregnant woman, it is very beneficial, especially for the child, because if you use the seeds, the child will be born with a healthy body, it is very important, it is important for the skin of the child, and in the mind, the brain will be open, ehhh, the fruits are also useful for the pregnant woman’.*

*‘ I think they were mainly concerned about the most prohibited drugs, such as alcohol, tobacco and food that do not meet the categories mentioned above, which are not strong, prevent diseases and body builders, so it is good to eat foods rich in these nutrients, but it is also better to avoid those foods or drinks that are not of good quality because they affect the child’*

*‘ :Beliefs are not there, maybe I don't know the beliefs you want to talk about, if it is related to religious beliefs, related to other religious beliefs, it is a condition or command you are not a church believer who has spiritual food to mix food that does not match with spiritual food’*

*‘it is taboo for a pregnant woman and a child to take alcohol. If a pregnant woman consumes alcohol more frequently during pregnancy, she may give birth to a mentally abnormal baby”*

*“A pregnant woman is not allowed to take any type of medicine without the doctors’ recommendation, not even traditional herbs because that might cause her to miscarriage or even risk her life to death.”*

*“No child is allowed to take alcohol. We always warn parents not to give their children alcohol because they say that they serve children alcohol for preventing intestinal worms.”*

*‘ The first reason for what they eat is related to their beliefs maybe for example like the Adventists maybe the reason I say it is because maybe when you feel like the Adventists they are not allowed to eat meat because they are not allowed The Bible says that there is a meat called turbano or even holy, they did not leave it, so these turbans were the food of the devil, that is why they were cursed, so no one is allowed to eat these meats, At that time, if you ate meat, you would be considered a foreigner. At that time, you were eliminated of their believers, Then it would be done for those who were priests who were allowed but also for the sacrifice that was made holy and I say the sacrifice that is one of the things that you hear okay, so that's why their beliefs prevented them from eating meat and also made them help them not to eat meat because there were those Bibles that forbade the meat of their animals that they said were starving., starving, bathing in water, birds, there was a list so that they don't make them hungry and feel that they are hungry to eat those meats and they are forbidden, so they make it worse that the best thing is that they will be stopped and allowed’*

*“From birth to the age of six months, the baby should be given nothing else except breast milk only after that complimentary food can be started.”*

*‘ Even today it seems that it is like a general rule, but that's what it's called. Today, it's mostly enforced by some Adventist you will see that they eat beef and they don't eat pork, and you find that those who eat chicken don't eat slaves because they are starving’*

*‘ It seems that what I was about to say, he was still talking about it. You see, Muslims do not eat pork ,I don't know how they do it, but that's exactly what you asked us about , How about that question, you asked us about beliefs and found that a Muslim does not eat pork and a Adventist does not eat pork and those who are not hungry like mice are not allowed to eat it due to their beliefs.*

*‘ Regarding the belief that you are not a Christian if you end up smoking and drinking besides the fact that it is harmful to you and your child, If you don't smoke, you're not a Christian, and you're not a Christian if you go and drink alcohol, and if you look at it, that means you're giving it up’.*

*“The child should be given milk and then the SOSOMA porridge, this is because a child’s stomach and his digestive system are not able to process solid food like that of an adult.”*

*Adventists, I heard, do not eat meat, do not drink milk, I have heard that their children are not given meat and milk.”*

*“A pregnant woman can face the issue of anaemia because of eating an unbalanced diet.”*

*“As the child grows, they are introduced to other foods as the mother continues to give breast milk until he turns two years old.”*

*‘Because of what your heart has chosen in your beliefs, you must follow the rules of what you follow because if you cut in two and go to your own, then you are different from what you agreed, so the best thing is to follow what you choose’ .*

*‘ you start feeding your baby when he is six months old and breastfeed him for at least eight hours’.*

*When the mother has died or is suffering from a certain disease with which she cannot breastfeed her baby in that case you can give your baby other foods such as milk earlier than even 6 months.”*

*‘ He should be weaned from birth to at least two years’*

*‘She said that the child is given support for six months, otherwise the child is breastfed for 12 hours, so the child is given support like that for six months, sometimes the mother is not satisfied , you must give him something to drink every six months to eight months and give him fruit or food depending on whether the child is hungry or if he wants it when the child is breastfed for at least two years’.*

*‘ When a baby is born, you breastfeed him without anything else and breastfeed him/her between 8 and 12 hours a day. How do you breastfeed a small baby ,you breastfeeding him or her like you give him time. For example, if you are going to breastfeed the baby, you are not going to cook potatoes or go to clean you take the baby and give him the breast and suckle him and comfort him, then after he comforts you give him the other breast and suckle him If the child is hungry then at six months you give him a complementary like porridge but food and give him food but not only food or fruit. but then in giving him food you know the amount of food you should give to the child if he is 6 or 7 or 8 or 9 months old you give him food depending on the months he has and prepare it according to how much it is and what you control food and I would like to add that there is no way that a six-month-old child is a child. There is no way to give him oil or give him salt, but you should give it to him’*

*‘The child is prohibited from receiving alcohol because this alcohol is damaging to the brain and also to the stomach. that means, the child who is given a helping hand is given a snack and is given fruit juice when found ,he is given clean food, but he is not allowed to drink alcohol because alcohol is harmful to his body, stomach and brain and causes him to have poor growth’*

*‘ a child who receives aid does not need to eat pepper. You all know that pepper has nothing to do with a child's body, and a child who is under one year old is not allowed to drink milk. I don't know how to get pregnant, I don't know how to say it, but a small child is not allowed to drink milk when he is less than one year old’.*

*‘ It causes allergies and you can see it even if you give him a lot. He is not yet a year old and somewhere you will find that the child likes to get sick from food and has allergic reactions on the body and you take him to the doctor. There are times when you take him to the doctor and they tell you to cut down on the milk that is given to the baby’.*

*‘ she mention what I was going to say on a child under one year old to give him milk, the nutrients in milk are often found in the intestines, so when you give him that milk and also while he is still at that stage he has not received enough support for his body to be able to fight the related effects in the body it is very difficult ,That's why the child has allergies or let's call it, maybe, to be clear, there are some skin diseases because of the nutrients that are combined and have many other things in his body, that's the reason to give milk to the baby when he's less than a year old is not good’*

*‘ when the child reaches the age of one year, you should give him simple food and mix it with vegetables, and after take it and crashed it and feed him three times a day’*

*“If parents have money, they will buy milk for their children. But if you don't have money! I wouldn't say that they drink milk, where would they get it from? Milk is for rich people; they are the ones who drink milk.”*

*‘I give my child milk and sometimes when God has been good which is rare, I afford the meat and I give them the soup.”*

*“Some people have a milking cow and deliver all the milk for sale; their children do not drink milk. And who has chicken can’t eat an egg, because all are taken to the market for sale.”*

*‘we should feed a child who is six to eight months three times a day but in reality, when we are on the field, cultivating, a child can only eat once a day. Because the work hour is so long. When we start to feed a child once a day, he starts to stunt. Therefore, we ignore it, yet we know it.”*

*‘ the child must be fed at least three times a day because otherwise we are not able to compete , sometimes you can say that my child is at least in the hours from noon to come down I give him three or two times where he wants, but otherwise because of his limited ability, at least three times a day the food you have prepared for him is the one that builds the body, protects against diseases and strengthens the body, but let's not forget that he must eat animal products’.*

*‘First you give him nuts as he gets used to them and cook him easy meals that include vegetables , small fish and then you feed him and give him porridges three times’*

*‘ a child who is not yet a year old or who is starting to receive complementary food you should first feed him such as fruit and give him a snack and cook him with simple food such as potatoes and vegetables, but avoid frying them because this oil is not good for the child’*

*‘ A child like that does not have immunity in his body like that, so give him oil, maybe give it to him like cow's fat, don't fry it, but put it in food without frying it’*

*‘, what I would assist is to a child who takes complementary food is not to give him oil, it is a mistake, but avoid frying as we fry, first we add oil, then we add onions, then we add tomatoes, then we add food no rather, it is better that you prepare these baby foods when they are fresh and take as much oil as possible. We don't pour the onion into it so that it tastes better, but you put it in, I call it putting it in. Put it in or throw it out as he said, throw it out and then after mixing and remove it, what you avoid to do is to fry them first’.*

*‘ Why is it not good when you put it on the fire that you put it on and sometimes it breaks down, which means that you have already burned the nutrients in oil ,It happens when you have killed all the fat and then you feel that when you kill the nutrients or the oil that can create fat that in him it is not found then, it is better to put it in without fry it’*

*‘ This means that when the baby reaches the time of his meal, it is better that the oil is cooked in his food Is there any oil you should use when cooking the diet of a premature baby because you see that you are using castor oil, it is necessary to apply palm oil , when you pour it and cover it with vegetables and boiled potatoes or even with a potato you have put in the green vegetables and small fish and palm oil and cover it with palm oil especially because it makes the child bleed to be strong, then the child will have strong blood, his blood will not break water because of the palm oil that he cooked in his food in his meal, otherwise the child will be as if found so people almost all have the ability , a child who is learning to eat should eat about four times a day due to the fact that he is training and that's how they told us that the size of a child's stomach and his fists are also the size of his stomach’.*

*‘ here in our village where we live, the people have cows but not all cows taken by the people of the first class, the first class and the second class of the family then even those who don't really have , we buy it and you try to see if it's even half as much now, what's left is to sell it, it used to sell for 150rwf, now it's 400rwf, Therefore, cows are not owned by all the people who are owned by the first and second class of people’.*

*‘ Also, even if it is the first and second class, not all of these classes will have it, so those who are in the first and second class will have one percent’*

*‘ This child is not yet ready to drink, : but only those who have them try the possible way to give milk to their children or the mother who doesn't try what possible way to get that milk for her child’*

*‘ there is a level one is in but try to at least search at least three times a week but if you find yourself feeding your child without having it, you will not always give it to him because you don't have it, but the time is about three times a week and you give your child half milk three days a week’*

*‘ As for me, because I know what is needed for a child, I don't have to go and buy it for him because I know that it is necessary when it is time to move, so I go to buy it for him’.*

*‘Due to the limited ability of a person to sacrifice at least twice a week, he cooks that egg for him and eat’s.*

*‘ I have chickens but my baby is not ready to eat that is, even if it is not time to eat the egg, you can also put it on the breast’*

*‘ I heard they were telling me that giving a child who is not yet a year old potatoes is bad because it causes worm’.*

*‘ that's how you heard the same way you heard the news that came to me when they told me that giving meat to the child who did not raise teeth. It's not good, his teeth will not come , so maybe that's why I didn't give it to him’*

*‘ I heard that they say that if a child does not know how to say that they do not give him stomach meat, I am not capable of cooking it but I heard it’.*

*‘ I used to understand that any meat given to a child that can cause child not speak quickly’*

*‘ I understand that the first person to give food is a pregnant woman followed by children under five years old and others later ‘*

*‘ I give the baby first and after I give the baby I follow the others and give them later’*

*‘ that means a pregnant woman are get hungry very fast ,is the one who should eat first followed by the children and others prepare lunch as usual but there everyone eats’*

*‘ the first thing I used to do was to see a small child who was also difficult to him/her so that I would be there and I would eat’*

*‘ once the meal is available, someone first prepares the little child because he is sleepy. you expect that he will not sleep without eating, then when you prepare and feed the baby, it will comfort him and then give others to eat’*

*‘ The chief is an adult who must wait for me first and then I will prepare the children and then I will prepare him and approach him and make him appetize’*

*‘ I used to take care of myself, ehhh, I took care of myself once I felt better and then I got more energy to take care of others, but I took care of myself first’.*

*‘ I used to eat first because a pregnant person is very hungry. I used to eat myself first and take care of myself, after fully myself, take care of others’*

*‘ the first thing that causes it all is poverty. This is what makes things go wrong because I will give you an example of a man who would not go to buy a piece of meat and you are also pregnant with a small child and you will say that I should go first, If he buys a piece of meat, I will cut it first and eat the meat. I will eat the meat then give it to my husband. I don't give meat to the child ,but it what caused by poverty and low ability because if it is the right one, it is the right one but if there are three kilos that we all eat in general, we would be enough, then regarding the quality of the food, the child should be given according to its quality ,I feel that a child should not be given food that comes from factories that are made in factories, we can give a child vegetables, that is how I understand’ .*

*‘ Regarding the quality of the food to be given to the child, it is better that you give him things that you can plant because that is what you can plant ,maybe you should take it if you have found a matoke and look for it and look for those small fish there because then you would not say that there is big fish or the little one, you crashed and then take green vegetables and you will take those eggs and give them to him, then this is also the egg because there is no prohibition in believe we were talking about, mix it and then feed it to your child’*

*‘ The best quality food you can feed your baby is that it has been prepared and cooked properly with clean hands and the dishes cleaned and well-prepared clean vegetables, small fishes all with hygiene’*

*‘ For me I am able to get green vegetables, sweet potatoes and cassava for my child’.*

*‘ I am able to get cassava paste, banana and sometimes Irish potatoes’.*

*‘ I am also able to get cassava paste, banana and sometimes Irish potatoes. Thank you so much’*

*‘ I can get Irish potatoes, carrots and silver fish’.*

*‘ I can have banana, green vegetables, carrots are also available since we have vegetable plantations and I of course have to clean all of them, then mix accordingly and cook to soften them for easy consumption by the child’.*

*‘ When am serving meat at home, the big part is for my husband, then I and my child share the remaining’.*

*‘ I also do it like that. A man has to get the big portion because he is the one who bought them obviously’.*

*‘ others just take the bloth(soup)’*

*‘ Is that soup at least available of course as the boss in the family before serving I have to first put aside his portion according to how he eats and others share the remaining food’.*

*‘ It is a mandate; can you refuse to give the one who gave you? Impossible.’*

*‘ I think if you refuse to give him accordingly, next time he cannot bring anything at home so that you can at least have that soup. Therefore, you should be considerate as he also considered you. Thank you’.*

*‘when you are living with your husband at home alone or with others, you should all know one another, know your preferences and behaviors. Therefore, you have to consider your husband according to how you know him’.*

*‘ when I am preparing meat, before frying them, I have to first get some soup and unfried meat for the child because they told us that it is not advisable to give fried food to a young child often’.*

*‘ Obviously the man is the one to be given meat first. There is even where you as a mother pretend not to be eating them so that you can sort it out may be according to less available of those meat and you surrender them to the husband and children’.*

*‘ when the child is sick, since the energy is less, you try to find some food to help him or her get energy and go the hospital’.*

*‘ In general, a child should be given energy giving food so that he or she can grow strong and well’.*

*‘ a child should be bathed and taken to sleep and rest his or her brain’.*

*‘ there are those children whose doesn’t like eating but you should find what he likes most and buy it, prepare some good food aside so that that sick child can at least get appetite’.*

*‘ Let’s say sometimes a child might be liking rice normally, and you try to buy it for him or her but because of less ability you can’t afford much for the whole family, instead you buy a little for the sick child’.*

*‘ a child should be given medical care, bathed, wash all the clothes, shave , at least maintain the hygiene of the child's body and everything. You also told us to talk about how a sick child can be taken care of, I think she or he should be taken to the hospital and give all the medicines as prescribed by the doctors’.*

*‘ Any time you see a child showing signs of illness, maybe no longer wanting to play , loss of appetite, fever, cough, and flu; all those are some signs that may signal you to take a child at the hospital and take care as it should be by giving all the medicines as prescribed. This should make you know what to do for that sick child in order to get better’.*

*‘ when the child is very ill, you first go to* *the community health workers and have some medication as prescribed but sometimes it becomes worse and you are told to go to the hospital for further treatment’.*

*‘ there is how a child may get sick then when you have paracetamol, you can reduce fever and wait for some time before taking him or her at the hospital. So, some of the people does not take their children at the hospital because of that’.*

*‘ sometimes one can use local treatments like using herbal medicine in case of cough and after they can go to the hospital’.*

*‘ No , but sometimes the child may get very ill when I am broke and by then, I can be using herbal medicine’.*

*‘ there are some children who are not allowed to use those medications, for example, a child under 1 year but that one above, since he or she is able to eat something, you can give those herbal medicines like (agahuhu) before going to the hospital’*

*‘ since some of us didn’t know each other and they got to know, is also what happens. You may visit your fellow mother; you can know when you meet at the market or elsewhere. So when you talk to your friends, it becomes easier and they can tell you what to do. But it is advisable to immediately go to the hospital so that they can do further treatment’.*

*‘ I think the problem is of lacking the ability or even time. For example there is when you find a mother having little kids, working for a wage and at coming home maybe she find a child coughing, by then when won’t think of first going to the hospital because sometimes those 300frw might not be available. That is when she can decide to be using these herbal medicines or else go to the community health worker whom I can also say need money. So, it is not easy’.*

*‘for us we have the public source, when you fetch water, you boil it then keep it safely for drinking’.*

*‘ that water source is there but you find like five villages sharing it. So may be for her, she may have firewood for boiling that water but for us in our village it is hard to get even some for cooking. So, I also don’t drink boiled water’.*

*‘ No we don’t. These in homes are for rich. So, for us we meet at the public one when we are like five villages .*

*‘ I means that there when you go to fetch water and find many people there to the extent you can’t even have some for drinking. For me when I luckily get some I just drink without thinking of boiling it as even my colleague said, firewood is also problem.so for the canoes, water just come from the ground’.*

*‘ Since the woman is the one to fetch water, because a husband can’t do that and go for wages at the same time. But there is even when you send a child and drinks it along the way then brings a little just like for one person’.*

*‘at my home, water is fetched by women and children.’*

*‘ at home even a husband fetches’.*

*‘ maybe this one might be near but majority are far, you can’t be near and find a long line but when you are from far, you face the consequences. Sometimes you don’t even get that water and decide to get stagnant water aside so that you will not go bear handed’.*

*‘ the consequences of course, if you are pregnant and go to fetch water from far you will get tired and you can’t take care of your self by then, Since there is no one to help either husband or children’.*

*‘ of course he can do that in case he is at home because there is sometimes he can go for wages for example from here to (place) and if course he can’t come and go to fetch water’*

*‘ water is near but it doesn’t mean that if the man is available can’t help you. So since water is near, one can wake up early and bring water’.*

*‘ I can say that the problem is that you go for water, the you come when you are very tired, and you need to wash clothes for the child and do other things. So, it is a problem to get water from far’.*

*‘ Toilets are different. But at home I just have the normal one’.*

*‘ for me at home toilet is there, but the little kids have none, but when one is in need of it, I notice it and remove the clothes then show him where he can defecate. After I remove the waste’*

*‘ when am not around, I show my child where he can use as toilet, and when I come , I clean the place.’*

*‘of course the waste is washed to the valley by the rain water’.*

*‘ I Can say that toilet is available but a young child can’t use it, so by then you find somewhere you can put that child to defecate. But the problem comes when it rains of course water takes the waste to the water source in the valley, when it rains before you clean the place there is nothing you can do.’*

*‘ For me I have a toilet and the child I have can use it no matter ‘.*

*‘ I can use the pot, after I take the waste to the toilet then clean it ‘*

*‘ yes I have it the one which the older one used’*

*‘ I can say that I do the same to my child as my colleague has just said I showed my child where he can use as toilet and in case am available, I clean the place thank you’ .*

*‘ the habit of washing hands hm… Which means at home we have sinks everywhere with clean water and soap’.*

*‘ For me I don’t even have that sink, so luckily when I get water I use a cup and wash my hands’.*

*‘ toilet is available, we don’t have young kids, the young one has a pot, so we make sure after his toilet, we maintain the hygiene and it is the same with us too. We have a sink a round that everyone uses as soon as toilet is used’.*

*‘ For me I am near the water source, so I wake up early and fetch it myself’.*

*‘ we do wash our hands. We put water on the basin and wash hands with soap after using the toilet but there is no sink’*

*‘ maybe starting on myself, I don’t know that chance of poty and I can’t even afford those sinks, so what I just take care of is washing my hands after using the toilet because I know anytime I will breastfeed. So for me I can’t deceive you’.*

*‘ the first challenge is lacking all the means of treating water for example, the jerycan you take to the source to bring water is the same that will be used still to decant water for drinking . The second thing is the way how we water is far because you may send a child to bring water, ant 3 hours lapses still waiting because of many people there or long journey. You may also think of making that vegetable garden in order to save money that would be taken at the market, then they dry up due to lack of water. Therefore, water is a very big challenge’.*

*‘ the first challenge is that water is from far, it was near, I can keep that hygiene with all my ability’.*

*‘ We all don’t get that chance of having vegetable garden because you can’t be renting house with one room and living room, then expect to have the garden for vegetables, so we mostly get them from the market’*

*‘ the challenge as you see is that water comes from far, and climbing the hill with a child at the back with a jerycan, is really tiresome. It is hard to get 2 jerycans o water’*

*‘Pregnant woman is supposed to take energetic food including sweet potatoes, cassava and rice, the body building food include beans, animal products, and protective food include generally fruits and vegetables”*

*“A pregnant woman is recommended to take green vegetables, meat, rice, fruits mostly those that increase blood in the body so that she can keep being better “All those foods can help the mother to be healthy and the baby in the womb to grow well and receive the required nutrients’*

*‘these foods are better for a pregnant woman since they contain all nutrients needed by the pregnant woman and the baby in the womb.”*

*‘ of course after fetching for once, you feel tired since you need to also take care of your child’.*

*‘ The best thing in general is the way of…because sometimes water is always needed and by everyone. so the only challenge is of getting drinking water. The problem is , I just get firewood for 2000frw and I will need to cook food and boil drinking water as well , how can I always get money to buy firewood? Therefore by then, if one gets a way, they boil water for drinking and if not, they leave it. It is also difficult to give un clean water to a child or even the mother since it can result to other infections. So, all these are challenges since we can’t find firewood easily or other means’.*

*‘ Something to be done , we cannot say that everyone will have a tap because some might be renting but at least there can be other alternatives that can be individualized to help in getting clean water. ‘*

*‘ for me the thing that I see to be done is advocacy for water, for example if you can go around many places, you can find electricity but it is not easy to get water. I would like to ask for advocacy so that deep villages can also have some sources of water like taps or any other way’.*

*‘ The first thing to be done is maintain the hygiene of the child and even to that pregnant woman. Even on us who are breastfeeding. therefore I think if we can have the way of having clean water for drinking only like disinfectants, it can be helpful. Other things like washing, we can go and fetch water even if it can be tiresome but at least having some water for drinking’.*

*‘ A pregnant mother should eat body-building, immune-boosting, and energy-boosting food’.*

*‘ the priests are the ones who teach us that we must eat vegetables, drink cooked water. They also teach us that we must drink porridge and what should porridge be made of. That's what they tell us.’*

*‘ pregnant woman should drink water mixed with green clay until she is five months pregnant. You should also drink enough clean water. Moreover a pregnant mother should drink Rosemary, maybe you should take it from the time you got pregnant until you are five months old, because after five months drinking romary can induce labor’.*

*‘ Rosemary has the same indications as green clay but also when you have insomnia, taking rosemary can help you to easily fall asleep. In addition to that, rosemary can help women who tend to have a problem of always feeling tired and exhausted during pregnancy expecially in the first 12 weeks. So drinking water mixed with rosemary after meals can help to overcome tiredness during pregnancy’.*

*‘ a pregnant woman should eat fruits, vegetables that are all I know’.*

*‘ A pregnant mother should not take medicines for threadworms’.*

*‘ I heard that if a pregnant mother takes a pill for threadworms, she may miscarry’.*

*‘ A pregnant mother should not smoke or drink alcohol because it harms the baby’.*

*‘ the baby should be breastfed at least eight times a day’.*

*‘ A baby should be breastfed from birth to two years of age’.*

*‘ A child should be breastfed until s/he is two years old’*

*‘ When a child reaches six months, you should introduce complementary feeding for the baby’*

*‘: at six months the infant’s stomach is expanding and usually want to eat solide foods’*

*‘: a baby should be breastfed until six months old. Then At six months old a baby should be given solid food. Because at this level, breastfeeding only is not enough as it makes the baby bites your nipple while breastfeeding’.*

*‘ A baby who has reached the age of six months is not satisfied with the breastmilk anymore. So getting solid food helps the baby keep up with her/his growing needs’.*

*‘ when your baby is 6 months old her first foods need to be soft so they’re very easy to swallow such as porridge or well mashed fruits, vegetables’.*

*‘ what not to feed a baby is for instance sweet potatoes because it is hard to swallow as she is just learning to chew’.*

*‘ As far as I know, when it is time to provide solid food to a child, you start with porridge made of corns for a week so that her intestines would get used to solid food. Then After a week, you give her porridge made of a mixture of corns, sorghum and soybeans. An infant should not to be feed wheat-made porridge until she is one year old because wheat work on the brain which can make them work in an abonormal way. What I do as a mother, when my baby is not yet one year old, I only feed her fruits like mangoes, papayas and bananas. Moreover, for fruits that contain seeds, I remove those seeds before feeding them to my baby until she is one year old’.*

*‘ In the first year of a child's life, would be given three meals a day, including fruits and vegetables’.*

*‘ when you have means, you start complementary feeding by giving soft food to prevent your baby to suffer from constipation. Among the foods you should feed the baby, are pumpkins and peas’.*

*‘ the kind of food you would feed you baby during his first year should include a variety of soft foods such as beans, fish. You should make sure that the food is so soft and no added oil or fat’.*

*‘ the child eats five times a day. She eats in the morning at 8h: 00 AM, 11H: 00 AM, 1h: 00, and at 16h: 00. Your baby should also eat at 18h: 00 so that she can sleep well during the night’.*

*‘ children in their first year are forbidden to be given cold food’.*

*‘ a one or two year old baby should not be fed corns because babies in their first year are unable to chew corns’.*

*‘ under one year-old babies should not be given nuts because it can lead to nosebleeding’*

*‘: when it is mealtime, we first wash our hands and pray then eat’*

*‘ when food preparation is finished, we put the food on the table and pray then we eat’*

*‘ when I finished cooking, I prepare the table and bring the good for us and children’.*

*‘ a pregnant woman and a child are the ones who are allowed to eat more times than others because a pregnant mother needs the food of two. Children need to eat often to grow well’*

*‘: children and pregnant women many times they don’t have eating schedule’*

*‘ when you are going to prepare food or feed you baby, you must be clean. Let say if it is potatoes, you have to peel them first and wash them propery before you cook adding to that, before you cook vegetable, you have to wash them two to three times. Pan for cooking and plate should also be well cleaned’.*

*‘ when it comes to quality of food for children, it is better to avoid feeding spoiled fruits to your baby. It is advised to feed them flesh fruits because they are the ones which are healthy and have nutrients their growing bodies needs’*

*‘ food available in this area is like vegetables, rice, potatoes, maize flour’*

*‘ other food we cultivate and feed to our children includes corn; sorghum and the porridge we prepare for them is most of the time made of corn and sorghum’*

*‘: husband is the one who is served the big portion of the meat because he is the one who bought it’.*

*‘ wife is the one who eats the bigger portion because she is the one responsible for cooking’.*

*‘ after cooking meat, I give the big size of the meat to my child who is able to chew meat’*

*‘ when we have meat at home, children are served first then me who cooked because sometimes my husband comeback home drunk and might not be able to eat. So we keep some for him to eat in the morning but still he is the one who is served the big portion of the meat’.*

*‘ you should include vegetables in every meal you cook’*

*‘: children often need adequate rest and hygiene. They should also nap during the day because they are young’*

*‘ you should wash your hands before you prepare the food and then wash your baby’s hands before they eat’*

*‘: when a child is sick, s/he should be taken to the physician’.*

*‘ when a child is sick, you take him to the physician. However, sometimes we don’t follow up to make sure that the baby takes the medicines as prescribed. So it is impotant to give you baby medicine on a regular basis until she recovers. Besides, you try and make sure that your baby eats well so that she gets better quickly’.*

*‘ when the child is sick, antibodies reduce which affects their immune system. So it is crucial to feed your sick child energy-boosting food and food that boost the immune system’.*

*‘ what you feed your sick child depends on what s/he likes; hence I buy and feed them what they like’.*

*‘ when baby is sick and can speak, once we comeback from hospital for treatment, I ask what my baby wants to eat then give it to them’.*

*‘ when you see that your child is sick, you should take good care of her/him’.*

*‘ you should look for something good to eat for your sick children and take good care of them during the period of sickness’*

*‘ sometimes they are things physician ask you to stop giving to your children when they are sick. When a child is sick all they may ask what to eat what is not good to their health. it is your repsonsibilities as a parent to feed them what is impotant for their healthy’*

*‘ I take my sick child to community health worker. When the CHWs cannot provide treatment to your child, they transfer you to the nearby healthcenter for further support and treatment’.*

*‘: sometimes you take your sick child to community health workers for treatment and medicines, but when CHW doesn’t have medicines you go buy them in a private pharmacy’.*

*‘ before taking your baby with high fever to the physician, first you soak towel in cold water and put it on the baby’s forehead to draw the fever out.Besides, there is another way you can use to help reduce high fever which is to take a mixture of one spoon of orange juice and spoon of olive oil for your baby to drink. Once you are getting ready to see the doctor, you baby can drink the mixtureOnce your baby drinks the mixture, the temperature drops which helps in cooling the fever tomporarily when you are getting ready to go see the doctor’*

*“ you would know if you baby is not feeling well because their behaviour change once they fall sick .if your put you baby in bed and the baby woke up with different mood, you will realize that there is a presence of illness’.*

*‘ if you raise well your children and spend a lot of time with them, you would know when they get sick. Your child might like certain things in daily life but if they refuse to do one of the things they usually like it may be one of the first signs of illness even if there are no other signs present. A distinct change in their daily activities may signal illness’.*

*‘ yeah, we have access to safe drinking water from faucet since many years ago’.*

*‘: we have clean water we get from tap’.*

*‘ the best way to drink water is to first know your weight. This is to say you may drink enough water in the morning and not need to drink water again the whole day’.*

*‘ in my village we have been provided with WASH services. As we have access to it (water, sanitation and hygiene), we get from there water to drink and water for domestic use. Though we don’t drink water from tank, when it is rainy season, we get water for domestic use from Tank we have at home and when water in the tank is not clean, we use sureau as treatment method of water’.*

*‘ we have latrine, constructed with good materials and has the door’.*

*‘we have our own toilet’.*

*‘ we don’t have flashwater toilet, the floor of the latrine at home is made of logs and pit’.*

*‘ we have flashwater toilet’.*

*‘ we have the step and wash near the toilet at home’.*

*‘ the washing station has water and soap as well’.*

*‘ often during the dry season, fresh spring water tends to be lacking’.*

*‘ where we reside, as we are in the rainy season, water is available. But in the dry season water tends to be scarce. You would travel two hours just to get water’.*

*‘ when we are in the summer season for instance in July, we tend to be lacking water. You would travel two hours to get water to use’.*

*‘ if they would help and provide fruit trees and avocado seeds’.*

*‘ if a husband has a child and pregnant wife, he must respect and provide all the important things they may ask of him’.*

*‘ if you see that what you are craving for is harmful or not important, you should not take it. You wouldn’t miscarry just because you couldn’t get your cravings’.*

*‘ I also think you would miscarry not because you couldn’t drink alcohol but because you had your thoughts involved in it so much’.*

*‘ pregnant women have conscience and the mind to think, so if you are craving for something usafe, you should put in mind that you should not take it, surely you and your baby won’t have any problem because of that’.*

*‘, otherwise the baby must breastfeed for six months, then after six months he/she should receive complementary food and be given small soft things like fruits like simple things thank you’*

*‘ I understand that a child should first breastfeeding the breast-milk for six months without adding other else and then after six months he should be given complementary food until he knows how to eat in two years and beyond’*

*‘ I expect What determines the initiation of complementary feeding to the child is that after six months the breast-milk alone is not enough and the child needs complementary food consisting of fruits and vegetables how the baby throughout the level of growth, every level they exchange food to give him, after fruits they try soft vegetables or porridge, eggs, mashed food, Irish potato, sweet potato until then he/she will eat a meat, I understand that the baby growth level determines the initiation of complementary feeding, especially after six months’.*

*‘ the child must breastfeed for six months without give anything else, after six months, they will give him complementary food, they must give him, disease-preventives food, energy giving food, body building food those including fruits, vegetables, cereals, must feed him/her depending on his/her growth level when he/she has a year and a half or two years and seeing food to help her/his which doesn't bother him/her’.*

*‘ The Complementary food the child should start when they start give him complementary food. They found for him fruit like maracuja make the juice of fruits, when he grows up at the level he can eat, they give him green vegetables mixed with beans, animal products like egg which is fresh or boiled That's how I feel. Thank you(cooked) until he raised the energy in each growth level, they continue to give him disease-preventives food, energy giving food, body building food because mixed food is valued, thank you’.*

*‘ I hear what others they said but first of all he should be given milk and then the SOSOMA porridge what else I hear what they said all’*

*‘ I think that a child should not receive alcohol or tobacco, those I think should not be included in complementary food’*

*‘ there is way of cooking vegetable with adding pepper, in complementary food during cooking the vegetables it’s not allowed to put pepper into’.*

*‘ there is something prohibited in the complementary food because the child who start given complementary food, his stomach and his digestive system are not able to process solid food like that of an adult, example in complementary food the maize corns are not allowed because his stomach can’t digest it, and those cigarettes are not allowed in his life because even an adult is not allowed, but for a child it is very forbidden as well as alcohol.*

*‘ I think that the reason why a child is not allowed to be given alcohol is that alcohol does not contain vitamins and I think that if we are looking for a healthy diet for the child, we are there for the child's health to go well and that is why we should not give him alcohol, because there are no nutrients in that. then he should not be given tobacco because there is no nutrient in tobacco, so that is why I think the child should not be given it while he is still taking complementary food, thank you.*

*‘ this kind of complementary food allowed for the child, we mentioned balanced diet that consists of vegetables, fruits and animal products all help the child grow and grow well in standing and thinking(mentally), it helps him to grow up well, but What they said about Tabaco and alcohol beverages makes him stunted mentally and physically it where differencing from that a healthy diet helps him.*

*‘ I think that the reason a child is given a balanced diet is to make the child grow mentally, physically and emotionally, and learn to live well with other people and be intelligent this why we give balanced diet to children.*

*‘ from six months to a year. The food he should be given is porridge, vegetables mixed well with beans and peas but which is soft and then add milk, eggs and fruits.*

*‘ Several times a day, maybe in the city it's different from rural, we do it three times in the rural area, but in the city, they eat six times*

*‘ and as he said it depends on the financial capacity of one's home if one tries to give it to him in four categories.*

*‘ a porridge mixture with SOSOMA, vegetables, eggs and milk if available.*

*‘I have my own opinion about what we should feed a one-year-old child, I think we must give him vegetables mixed with beans or mixed with soft potatoes, eggs, milks and porridge.*

*‘ then times should he eat a day, I think that mainly nutrients are still in the mother's breast-milk, so she/he should eat twice a day, without eating too much food, in order to takes those nutrients within breast-milk.*

*‘ otherwise a child should eat four times a day, but because of the people's ability, sometimes he eats twice, then even those fruits, vegetables, eggs, porridge, milk, and you find that there is none in his house, and it is difficult to find even porridge. Child should eat a banana, there are no fresh bananas , maybe he can take them to market to buy banana for baby, the reason why maybe the child does not grow well is because some foods are not available, but if available, the child should take all that they said, and the child be well and has a growth, you can see him and say that this is a good child, so in our area there are those who have all these things and there are those who don't, that's why children differ in their growth, thank you.*

*‘ what is allowed in one year is that he must eat vegetables mixed with the beans, then he drinks milk, he gets eggs, he drinks porridge, and then banana, then he should be healthy.*

*‘ Otherwise, I see that the first prohibited foods for a one-year-old child are sweet potatoes, maize corn, and to give him alcohol beverages this is forbidden for baby.*

*‘ The reason why it is forbidden to eat maize corn is that a one-year-old child does not have strong teeth to chew the maize corn, and their stomach can’t digest it, potatoes peel the stomach is not able to digest it properly that can come out of the womb, so it will cause the baby in the womb to change and cause problems for the baby.*

*‘ in our area milk is available and there are people who have chickens and eggs are available.*

*‘ maybe there are some who breeding domestic animals, but if you find a person who breeding they will take milk and deliver it, then you find someone else on the side if he needs to get milk within his capacity, but he is not able to, so in short, these are two things that can make milk unavailable at home because it is taken to the market, then the other person has no ability to buy it.*

*‘ otherwise our abilities are different. There is a person who has a milking cow and delivers all the milk, his children do not drink milk, it available in our area. And who has chicken his can’t eat an egg, because all taken to the market, I understand that we would still have a low opinion, so that attitude should be broken, that's what I understand.*

*‘ about to take all milk to the market I will answer in the time of talking on that makes children stunted thank you.*

*‘ there is a problem, they said that the one has cows, their children are getting milk? There is where you go their caws are milking but children don’t drink it. They sell it all.*

*‘ in this area where we live in the culture that prevents children from eat anything we don't have a culture that says not to give the child anything, we don't have a culture that forbids you to feed the child vegetables, it only what based on religious, maybe say that if I don't drink alcohol, as a parent and my child don't drink alcohol and that's all.*

*‘ what I would like to add is the culture that I feel is similar to that of the church that we talked about before, saying that for example the church of the Adventist, it is the one that often forbids such as the meat of animals. Then parent say that if church not allowed me to eat meat so I can’t give my child animal products.*

*‘ before when we were adult children, the man was the one who was given the food before and huge. But now we have changed it and now we are talking about the child who is given food first, when the child comes home and eats then others follow and the mother, the man eats later or the pregnant woman is the one who eats first before all the children. A man eats what a pregnant woman eats and now we are talking about, but before the man was the one who ate before, there was no child who went to the pot they did not give to the father and that is how we are.*

*‘ Other than that, the child should be given food first, and his mother, perhaps a pregnant mother with little strength, and those who want to reach the level of strength of a child who is being adopted, because they are also raising someone else in their womb, and when that child is conceived, his embryo's conception should begin from where they begin to nurture. So, in the way of raising until the baby is born and continues to be raised, I first say that the pregnant lady must get the food of that also contains nutrients, such as vegetables, or mixed food and animal products and drink it so that the embryo continues to grow and is getting enough nutrients. So when a child is born and starts to eat, not if I went to work at home, maybe I eat lunch, not even a middle-aged child of another year who started eating would wait for lunch. In the morning, he drank and drank for at least two or three of those times, and he saw us and we helped him. He saw others at lunch time and we helped him. When I, the mother, the elder, the husband, if I went to work, I could come and eat at noon or I would eat lunch. I am not hungry, but that child should cry at least when he is the one who should eat before him and his mother because they are the weakest people in the house. Those who are considered to be underprivileged is to help them in the form of nutrition thanks.*

*‘ In terms of quality, otherwise the child should eat food that is well prepared and clean and the food that is fed to him is clean, that's how I understand it.*

*‘ I also understand the quality of food that should be prepared cleanly. Then the child should be given a complete diet, consisting of: disease prevention, energy and bodybuilding. It should be prepared and prepared for cleanliness and they should be kept in a clean place.*

*‘ Let's say it's like going to prepare milk for him and boil it first so that the germs die.*

*‘ Perhaps going back to energy providing food we find potatoes and grains; food preventing diseases, we would find vegetables and fruits; and animal products for body building food.*

*‘ What to do in the case of little the meat? A woman does not eat meat, will she eat it? She takes the big part? It is me, the man, who eats the best meat.*

*‘ Normally, it is the man who takes the big meat and yet the child should take the big meat.*

*‘ Thank you, regarding the food of meat, I differ from men. As a man, meat is a one-time event, we will not eat as usual every time, but then how about us as men sometimes we get to a place where there are meat , It's not the meat you want to eat, but they won't find it at home; That is to say, when meat is found at home, we focus on buying women and children, because we look at it like this, for example, I am here. They are at home. So if I see that meat , I buy and eat? It's like they're the ones who need it .*

*‘ when he said it, it would have to go, but because people did not persuade me, I told you that the people of Kigali eat six meals, while we only eat three meals. You understand where they are different, that is, I am a meat eater, but I only afford once an animal is dead at home; So when I saw it at home, that's why I said that I'm the one who eats it, because I consume the whole of it give them some simple soft pieces.*

*‘ I wanted to talk about chicken meat because chicken meat has the one meat, so don't say that this one is big and this one is small because all the pieces are equal since sometimes I am satisfied with it. The portion that can be eaten is cooked so that in the morning by reheating the remaining sauce the child will have something to eat again.*

*‘ Furthermore, in terms of meat for women, it is far from me because I usually use fish, men are the ones who usually cook meat, so you wouldn't cook meat to feed someone else, the wife and children eat little*

*‘ I'm talking about the meat food at home, otherwise the man is the one who eats the meat because I think if it's the chicken, he's the one who prepares it, yes, it's mostly the man who likes to prepare it, so when he cooks it, you should want to feel that it's cooked and as they said. The best meat is the one who eats it, so it means that if they understand the RESPONDENT of meat, he should eat two meats and he should eat three, then we will see it. Even if I don't eat meat, it seems like it's the same as when we go to the center, the meat is a special meal. That's how I feel about it.*

*‘ how to care for a child, or other children in general. There are things that we don't have at home, so you would go to the market and buy them and take them home or the lady would go to the market and send them to her; However, even if you are going to look for other stuffs don’t ignore that these little things must be brought to us.*

*‘ for example, small fishes, there are vegetables, tomatoes, carrots, and peas.*

*‘ I take care of my house in a different way. And other small things such as eggs to make a mess, but the others went to school and immediately take the potatoes and eat and we will share the potatoes with you. Now you are feeling that I care in a different way, but why? He has limited abilities.*

*‘ I am concerned with child care depending on the ability of the children we care for in different ways, the first thing we are competing with is to make sure that this child who is under five years old does not come to school. So the other adults who are over five years old share the normal things depending on our ability, we have what we have from the fields and what we eat, but the person who looks the most is on that child who can say that he is crying and we are helping and others are living a normal life like for adults.*

*‘: How to take care of a child is always the responsibility of the mother, you must know that at least the child has a meal? If he was able to eat, what meal did he get? Does he have a good dress? Did he wash? Now a day when a child like that little one needs to have time to rest when he is at home and his mother makes him sleep for a year and he has time to rest, I understand that this is the way to take care of a child. If you know that he has eaten, if you know that he is dressed and walking, if you know that he has at least washed, if he has eaten? For others things go on, life goes on.*

*‘ In the way of taking care of children, you see that there are children who are under five years old, where we live, we often take care of them and those who are over six years old because there are foods that they are unable to chew like we mentioned, but there are older children when they are able to chew even the potatoes and potatoes. Now for young children, how do we distinguish between feeding them, you see that a small child does not have a pot like the others, you have to prepare food for him that is better than what the adults prepare for us as well.*

*‘ we prepare basic food for a sick child, among other things that prevent diseases, especially vegetables and fruits, which is what we focus on sending him, but then we give him and take him to the doctor for a check-up. The only intervention is a balanced diet that contains antioxidants and nutrients.*

*‘ Thank you, except for the sick child and everyone else and the elderly person who is sick eats better than what he get when healthy , why? They give him good food to help him recover as well as the medicine they give him.*

*‘ For example, the best food they give him is vegetables, vegetables, fruit, and milk. A healthy diet is what he gets right away to help him recover from his illness*

*‘ If the child is sick, we address the issue in the villages, to the health advisor, where we first take the child who does not like it, and he sends us to the health center or the post office, when the nurses examines him, if it does not work, and he continues this transfer until we go to the hospital, if necessary*

*‘ I also think about when you should take your child to the doctor because when you live with a child it becomes a puzzle when you see that he has some symptoms and there is no one you see to care the child's health in. You as a parent know how to root. Whenever you see a child who has physical changes, you should immediately take him to the clinic to monitor his health.*

*‘ I think the first aid as a parent is to first give him food, then you finish giving him food, maybe immediately take him to a health counselor. That counselor would give you a transfer and go to the hospital.*

*‘ we have life counselors in the village who keep coming to us in the villages and teach us.*

*‘ sometimes before you know when a child is sick, first you see the signs that he was sick then when you see the child's favorite thing if he starts to refuse, you immediately know that he has a problem, you change another one of his favorite things and he still refuses, and you immediately know that this child is sick, so help is added to that; do you come and wash him and finish the wash and feed him again and he refuses or eats as you did before and you say that this child is really sick and then you take the good thing where does it go? At the health Centre , he used to cry and now he refuses to examine him. The doctor would also go to the hospital and examine him and tell you why he is like that because he refuses the other things you used to give him, and you will immediately know that the child is sick.*

*‘ the help you would use for the child before you take him to the doctor, for example, maybe I give maybe if the child shows up, he has a fever, the fever is in his father and the doctor is the one who will check the fever but to find out why he had a fever. But like you, as a parent who helps him before taking him to the doctor, first of all, when you feel the fever, the father says that you can wash it like a towel in water. You first remove the towel and reduce the water in it and put it on him gradually without this surprise, so it helps to see how to get him to the nurse or the counselor. Then again, if he has signs that he might have pneumonia, they say that when a child is on the street, take him to the doctor, it is not good to give him a cold because the cold and the wind will increase the pneumonia. You go to the hospital and take him to the doctor and the doctor will do what he has to do.*

*‘ I heard it from the doctor because I had a child who used to have a fever. If he had a fever, you would have helped him and we would have laid him in the hospital on the patens. The doctor told me that the towel was helpful because he had a fever that was very high, over forty. Here because he has a problem with fever, use a towel on him and don't surprise him with a towel so that you can put it on him immediately. Remove the gas as it is reduced in water and then take it slowly and watch so that you don't surprise him and his body starts to cry or the body suddenly shocks. On seeing that he might have pneumonia, we cover him and protect him from the cold, because fever also brings pneumonia.*

*‘ earlier in the past years we did not have clean water but now our father has been kind to us and there are taps in our house.*

*‘ The problem of water is not going to go away according to the condition of the place where we live and now there are people I know who are drinking water from the ground like that of oxen and now there are people who I know are really low as villages that have reached their contents so I understand that the problem of the water is still not dissolved and we still drink bad water*

*‘ they put water in our house but not everywhere, but there are people that fetch flowing water.*

*‘on my side, there is water, there are two water tap and there is good water, but in other villages they come to us for water water and there are people who live in the mountains there is no water.*

*‘ I could get water myself because I live near a reservoir, but there are people I know who live near me and don't get clean water because they draw water at the pond.*

*‘ I am also talking about the issue of water, as this brother said, they gave us water, but it has not arrived everywhere. Now to my side, I live in a place near the extremities. The centre, where the centersnearst the rivers are growing, that's what the man said RESPONDENT three and we have a village that lives nearby that drinks water from the other side of the border. They don't come to get water where we pump at the market, we only have those to drink, and those to use for cleaning, they buy from someone else, and the market we have, I don't know if it's the one called the tap or maybe they borrowed it, so we don't trust it. Because I was building a community, we are putting clay and all that, and I see how the water and sand are getting dirtier like that. As far as water quality is concerned, we would not have access to clean water*

*‘ Near my house, we have a spring in the rock.*

*‘ water in general, we use a few taps, we get that water from the taps that are in the basins we built, even if there is one, even if we use a few.*

*‘ otherwise, the water we use is water from the market, then the people have collected money and built these taps and we water from them. Now they have brought these small ones and they are making them pushed by machines, but because they are selling them, they are still drinking from their own rivers.*

*‘ have a toilet, but it does not meet all the requirements because I told you that people are different, there are those who have toilets that are dry and sand, they are made of tiles and they are made of cement, but there are also those who have only made of mud, yes, some are covered with tins or trees t is not fancy but we have a bathroom. You asked for how long? All the time.*

*‘ Regarding the toilet, we are not one hundred percent full, we have about sixty-five in terms of toilets because the bathroom is flush but not closed, not closed to the floor. The bathroom isn't hundred percent full yet.*

*‘ In fact, we used to wash in the corona time when a person had to wash hands compulsory, but now it is to wash from a cup when a person is going to eat.*

*‘ that's about washing up as the brother probably mentioned. In that time of the corona it was like a goal, as a culture because there was no place to go or enter without permission, but now where the covid is under control so even entering here you will find there is no barricade, have you seen it now? So what is missing from the office could be found in our village? Not at all*

*‘ I see that it is normal and I take a bassinet and put water in it to wash but then step and wash it is not there,*

*‘ I think the problem lies with the management of this area, because they focus on putting water in the centers, then they put more effort in the centers, making the people in the centers, the people who live in the centers, because they are the leaders, they don't go home to get water. well, but we in the countryside they seem to have forgotten about these challenges that we see if there could be an advocacy like the administration goes to the countryside and sees how people drink dirty water and they go up the mountains and put effort so that the water reaches them so that sanitation is not available. Thank you.*

*‘ It is a problem that we have, there is clean water that we pump from the ground, trying to drink like clean water and doing like a trip of an hour or thirty minutes when a person goes to work and comes home in the evening, it makes him drink bad water and he can't find time to go and get it.*

*‘ water constraints, water here in our countryside is still scarce. I would like to give an example of the village I live in, we have a big village with maybe three hundred houses but we only have one water pipe. It's like a person who lives where our village seems to end can travel an hour and come to water because the tap water is where the village starts, then a person who is at the end and where he is going to come and drink to the other place where he started and makes an hour's journey. So you understand that it is still a problem, the water is still low, that's why you can go and find a place for him to make that trip and he needs to go and find another way of life. they are washing dishes, using the water that is flowing that is dirty because there is no pipe. The problem of water here in this village was a problem and we know that bad water also causes problems for people, you find people with snakes and we know that sometimes snakes bring problems with children.*

*‘ the problem of water is also a problem that breaks us and makes us think instead. Now, there were times when we went to the fields to cultivate, then you would come to take food and feel you need water or you would go eat and feel you need to wash. So I end up drinking water at the bottom of that little rivers instead of traveling to fetch water because I have not planned the meters in between where the river is, which is an obstacle. And the other thing about the rivers, they were in pricey who counted that the jug was three or five, it was 200 rwf, you would look at 200 and feel that you don't buy water and the child needs a notebook and pen for school,so when they come home in the evening, they go to fetch and walk for hours.*

*‘ the first thing that needs to be done is to change our attitude, the reason why I say that people should first change their attitude is that, it will change and other things will change because I say that you may find that this person has a chicken, he does not know that his child or his wife should be eating eggs , and find a person who cares for cows and does not know that drinking milk is important for the health of the people in the household, whether it is a child or an adult. So the main thing is to change our perception because sometimes we say that we don't have something that will help us and we do have it. A person who has a land to live on and has children who doesn't have a vegetable garden should at least provide them with these vegetables at the time, so there is something we have that can help us, maybe we can buy what we don't have if we can afford it. But what we have at home, for example, if I have a farm and I have a way to get the chicken, I should change my attitude that I shouldn't take it to the market for money while at home it is needed, if I have cows, I should change my attitude and feel that even if I need money, I should not make money from the milk because I "The family needs to drink it, and you find someone who sells it in the morning for seven days and in the evening, and we change our attitude and realize that if there is milk usually five liters, you can sell three and save the rest for the family to use." My opinion is that.*

*‘ what I think, as my friend says, our sense of understanding where we live is low, maybe I would have that kitchen garden, vegetables that I don't cook and there, I would have that if I don't drink milk and if I have, I would have those chickens but don't eat eggs and chickens hatch. So, the thing that will change people's perception is to find out how people's perception would change so that they understand these things that are really important for the health of children and pregnant mothers. For the merchant, maybe I will ask him what he will take and sell. These are things that he knows as a man. So we should also think about it if we know how to take care of ourselves as men but we have to take care of pregnant women and children*

*‘: otherwise, a pregnant woman should rest for a period of time if she is working, if she is farming, and do not do heavy work so as not to break and disturb the pregnant woman. As a child, a small child does not have to do work that breaks him because there is something that he can find, perhaps like a child who carry a jerry can, a five-year-old boy who use that to go fetch water. That, to me, is what makes a baby and a pregnant mother feel better.*

*‘: the first thing is the fact that we have to change first because there are many househo;ds conflicts tat affect homes so I think the government, our leaders should approach us every day to encourage us to live well so that our wives and children can have a good life. Thank you*

*‘ I think the only thing we can add to a pregnant woman with a child is to be close and provide the full diet and take care of their health...*

*‘ for the pregnant mother, a healthy diet is another way to take care of her other things that gives her energy, prevent diseases and build her body. So that when the baby is born will be healthy. I feel I have nothing to add*

*‘ I would like to ask you this question, maybe it's like a test in order to understand how we answer, even if you asked us and we answered without correcting what we said, we killed it or it didn't match the plan of what you asked us, you didn't give us a formula in what you asked us to keep it in mind.*

*‘ I believe that a pregnant woman can eat green vegetables, like amaranth,peas. Plus eggs and meat.*

*‘what I know is prohibited is the traditional medecine, because it affects them, I’am not so sure why but that's what the doctors say.*

*‘A pregnant woman is allowed to eat vegetables and other related plant products, animal products are allowed, what I know is not allowed is alcohol, pepper and herbal medicine. She should mainly focus on body building for immunity and energy giving food: these drugs affect the child very much because there is no dose that has been measured by a specialist. Those are just what they mix and drink, yet we know that the medicine is given depending on how much (weight, age) you have taken up to a given point.*

*‘ what a mother is allowed to eat during pregnancy includes stimulants, anti-inflammatory and body-building food. Mostly what she is not allowed to take are not even classified as food*

*‘I don’t believe the same way as my friend, that pregnant women shouldn’t eat pepper,same thing with ginger because it is not taken solo ,they mix it with some other food. Pepper tastes very spicy and therefore I don't think garlic ,ginger or paper are forbidden to eat in food and yet they are for flavoring food*

*‘, in my opinion i think , everyone has their own principle, according to their own principle, they can decide what to eat and what to not eat depending on the effect it has on their body. I believe that there is no religion that forbids you to eat something, but there are religions that forbid people from eating certain things. Because there is a period when a pregnant woman craves for somethings and i guess no religion would stop her from satisfying her cravings.*

*‘ I don't know any religion that prevents a mother from eating certain foods during pregnancy, instead everyone encourages the pregnant mother to eat foods that stimulate the body, build the body, and prevent diseases. So there is no belief that I know of that would prevent that mother from eating certain foods.*

*‘ I think this belief about eating for a pregnant woman, I think there is nothing that religion prevents her from eating. Only medical doctors have the authority to do so. Because they prohibit you from eating certain foods due to valid reasons related to your good health .*

*‘, a child should be breastfed for two years and have six specific months of breastfeeding only without mixing anything else. Instead you should be taking good care of the mother so that the child can get full nutrients. She should be eating enough animal products, eat vegetables, beans, amaranthus,spinach, and Isombe. and other vegetables, not forgetting that she should also eat energy giving food , so that the child can eat healthy breastmilk that is full of nutrients for the body. Once the baby is six months old then you can start to give it some fruit, some soft food and milk until it is two years old. Thank you*

*‘ the mother must breastfeed the baby for six months, before giving it any complementary food.*

*‘Usually there is no specific time of when a mother should stop breastfeeding her baby, but it is recommended to breastfeed it for two years and then get to wean it.*

*‘ I know for sure that every baby, when they get well breastfed up to two years they can definitely continue with their complementary food. The baby can keep up with the complementary food only ,yet she/he is even about to start school.*

*‘It won't be good for a baby to start going to kindergarten when it has been a few days when her mother just weaned him/her. By that time The baby needs to get used to eating regular food, not waiting for the breast milk .*

*‘ talking about the complementary food ,you start with light food, like some fruits. Soft vegetables like amaranths. You can’t give the baby eggs or some Substantial food.*

*‘ So what tells you that it is the right time to give your baby complementary food ,first of all it's when the baby is six month. The baby at six month is very greedy she/he wants food and the breast milk is no longer enough. Therefore I can say that it could be the right time for the baby to take the complementary food because even the doctor recommends so when the baby is six months.*

*‘ It is possible, the child might start to get complimentary food early due to her mother’s sickness or maybe because her mother is very much busy to get time to breastfeed the baby. You might find out that the mother started working before her baby gets six month and in that case there will be no time to breastfeed. Therefore the baby will be given milk earlier as an alternative to breast milk. Talking about the time the baby should eat complementary food ,they need to be six month first cause that is when the baby’s stomach is able to digest. When the baby is six they can give her/him some porridge ,milk but not any random type because for sure there can be different reasons that can make a baby take early complementary food .*

*‘ what the child is allowed to eat are the fruit. The type of fruit she/he is allowed to eat are passion, oranges, and they can also have some green vegetables, like amaranthus and spinach,and get him/her some fish meat.*

*‘When the baby is seven month you can prepare some potatoes and mash them. You can also give the baby the fruits like bananas,phosphatine, all the things that contain healthy nutrients. Then when the child reaches nine or ten months, you can also give him adult food, but in moderation. That’s my opinion on that*

*‘ I think that the child should be given the complementary food in a moderate way depending on how the baby grows up .When he/she reaches the age of six months, he/she can be given that milk, if necessary, he/she should be supplemented with a little bit of clean water, in case the mother is having shortage of breastmilk. That's my opinion about it.*

*‘ , literally you can not start with solid food for the baby’s complementary food ,you need to start with some soft food because the baby’s stomach is not strong enough for that food digestion.*

*‘What I think should not be given to the baby is alcohol. Their brains are still so sensitive that if you give them alcohol you can harm them when they are very young and if you give them bitter things they can harm their stomach, because it is not strong enough that it can handle it. What to note here is that we should not give our babies every random food because the food they eat varies with how they grow.*

*‘ She/he has to eat the food we have been talking about during the day, at lunch and maybe even at night.*

*‘ She/he can be given some bananas, amaranths sauce, green plantains*

*‘, in the baby's first year you should give them fruits that are mixed and in the child's diet you should alternate vegetables but if you alternate you can prepare them and they will benefit him/her. You can prepare amaranth, spinach, and isombe with some soft potatoes. Also, we have to alternate the child's food. Let's say that they got amaranth. We have to know when to give a baby a different type of food like blended potatoes and spinach. Daily I think that would be enough , give him/her three times, depending on how the child is because we know the baby eats little. As the baby is unable to say that they are hungry you keep on checking on them to feed them*

*‘ A child under the age of five eats breakfast before going to school ,eats lunch when they get home before napping and also when they wake up around 4pm they eat some.*

*‘ So here is what you give the babies in their first year. We give them rice, beans, vegetables and carrots, and sometimes fish meat though they are expensive but you can still get some dry fishlets as a substitute.*

*‘ there are things that are prohibited for that child, including pineapples, lemons, fruits that contain acid, alcohol. Other things are prohibited as they monitor the size of the child as she/he grows, as the stomach grows and becomes stronger, you should not take a one-year-old child to give him corn, give him beans, it is not good for the baby it should be selected based on his age. That is to say, from the age of six months, it starts to grow, and it goes up to a year. As he grows, they improve his diet.*

*‘ the way I understand it, a child who started to have complementary food under one year old, they give them some blended food as they have milk teeth, so you can not give them solid food, and even when we have prepared meat, we give them soupe. Unless you have a grinding machine for the meat. Otherwise , it can get stuck in the throat, and would get you in trouble for missing a child. Another thing is that you can't give a baby a mixture of corn and beans because it would also affect her/his stomach. Plus drugs and alcohol are not healthy for children.*

*‘I once saw someone giving his baby alcohol ,the thing I would never do! I asked him and he said that he was treating the baby from diarrhea,he said the child seemed weak. And you can tell when the child has been taking the alcohol ,she/he comes excited to get some. What I did was to remind that person how bad alcohol is to the child.Second, you must clean up the baby and feed them on clean plates,spoons and cups and make sure they are not wet. The child should eat flawless food that has not been warmed or heated ,because the microbes easily develop themselves in cold food. I always see it at my house. They let the dishes dry first and use them . You should also recheck if the baby’s food is slightly hot and cover it. The child is forbidden to eat food that has been frozen, I repeat it haha! It is better to give them cooked food right away,and give it to them when it’s time to give,plus keep it in a warm place so that it's always ready for them to eat. That’s how I understand it .*

*‘: According to what I see ,us as parents ,it depends on how your home is organized. In my home we prepare the dishes and we get together with our kids to share. In my own understanding the child should be having a caregiver who follows up his/her daily moves,that can also be her/his mother or me as a dad if I have time to make sure that the kid got something to eat before and after school because kids in that age are very greedy. You just make sure that you get energy giving food protein in fact a balanced diet.And also don't forget to let the kid have fun with his/her fellow kids.*

*‘ in my house, a child under the age of five usually has his/her own plate. That child got his/her elders, so I need to know if that child has eaten by rechecking at the plate we set for him/her if she/he managed to eat. I can not let him share the rice and vegetables with others. Once the baby shares food with his/her elders you won't be surprised to find out that the baby ate like five spoons only and the rest was eaten by his siblings.*

*But mostly when the kid is under one year we take of him/her and make sure we give the baby some blended food , after a year when he starts to learn to feed himself, he starts eating with us what we can, because we don't eat things that we can say the child can't, I mean like vegetables, bananas, rice, potatoes, You can also put him on his plate and see if he/she would be able to finish his plate alone.*

*‘ the food we provide for children is available in our markets, and in our homes. First of all, they encourage us to make a kitchen garden. Often at home most of us have a kitchen garden, we have green vegetables, spinach, carrots, there are parts of this neighborhood that grow potatoes, these are the foods that meet the needs of the body that a child can receive. Secondly, you can give them food that contains the dry fishlet flaw.You might also make a thick soupe of dry Fish and potatoes. That is considered as a food that fills the body with nutrients. I think that there is no one who can’t buy the strainer to make your own sauce without putting oil in it by just grinding it and giving it to the baby, and then when you go around changing it, you would not always give him/her the same food . you might the next day give the baby some eggs and some bananas. I think that's how it’s done here in our village*

*‘The child must eat clean food because the food that contains dirt affects the child from any disease that may catch him. The food available in this village is the same as the one we can find in markets ; good potatoes ,carrots, vegetables, beans and eggs fishlets are all available. The basics are available, maybe some are not available, but most of them are in the market because you don't plant them. Again it depends on your financial stability, but they are available, thank you*

*‘ What my friend said is true, you might not get daily milk for your kid due to your financial status. But if you had that ability you would definitely get some for them everyday. Depending on the number of kids you have ,you would know how to manage themor how you want to distribute them, but milk is available, and it comes from white cows’.*

*‘ Milk is available but it is expensive, that is to say that if one liter reaches five hundred from a farmer, then from milk sellers it’s six hundred it's understandable that its not everyone who can afford it’*

*‘ A liter is six hundred and five where the price is low, that is to say that the farmer who got his cows is the one who sells a liter five hundred.Other are those milk sellers who got Inyange Dairy,Nyanza ,etc and they sell one liter six hundred. Up to now one sachet of inyange milk costs one thousand.So you can tell it is not easy for every child to get milk to reach a thousand. So it is not easy for any child to get milk until’*

*‘ That cow breeding program here is available and it had a very huge positive impact on our people. For example, like I have it now. On the mountain where we live, 80 percent of the people have cows, and most of them got those cows from that program.That is to say, any resident you may ask will give you a testimony and tell you that there have made a step in life they will tell you that the children were malnourished, the children who had kwashiorkor are healed now and they get to have some for selling. They are very grateful for the program . Because with this program they would never have malnutrition problems as they sell their products, milk and sell the manure from it'*

*‘ Domestic conflicts will not fail to affect children because many times it is found that when a woman and a man do not agree on raising a child, they do not understand each other. Sometimes the husband is an alcoholic, and the woman is an alcoholic, now women are alcoholics haha, you get it ! we have them and don't have time for their children.Because raising and giving a balanced diet to a child reModeratoruires you to have time to make a follow up. and I think you can not get that time to make a follow up to your kids if you are always fighting , whether you are fighting or if you are trying to solve it, then the time to feed the child will pass. At that time, you won’t even bother knowing if the kids are clean’.*

*‘ Very very very very! I told you that when the conflict is at home, it's not only about food and housing, even if you try to balance it, everything will go back. So both that child and that pregnant woman get stunted. Because even if a person gives you that food, without giving you peace of mind that would harm your children and that kid in your womb.Because the life a woman gives it is to herself first first ,and then the child in her womb , I guess there is a connection even though I am not a woman, but you also understand that there is no way that a person can create a fetus to lose the connection with the pregnant woman, who is givin it life’.*

*‘ Domestic strife will not get you anywhere. It is possible that the conflict can be caused by the husband at home due to alcoholism or by the wife who often causes it if it does not result from alcoholism. Most of the time it can be resolved if they are discussed. So, a family with them has no peace, and their children get stunted easily and very Moderatoruickly. That child will not eat, that child will not be happy and it will be difficult to study. Thus, conflict slows down the crisis and destroys the development of the country’.*

*‘ Yes, I would like to add to my colleagues, when we look at who the conflict affects the most, it is the children, because when we do research and look, it is like the children in the street. You know, the way men are created we feel like we have other responsibilities, we don't really take care of our kids in terms of behaviors . Mostly those street kids will tell you that their mother left them and now they live with their stepmother or the other way round.You might find out that it is this couple that never get time to discuss their issues’.*

*‘ Let me tell you my personal experience in my house. When we have bought meat the mother gets the big part because she is the one who prepares it. But because she respects me a lot she gives more too’*

*‘ Like in families, how they receive things or how they prepare them is different. I have a daughter who loves meat and I love it too. So often when I get money, I buy it because I know she will love it. I don't tell her mother to give her more she already knows ,and I also wait to get served’.*

*‘ We have shared the same plate when eating since we got married, I may have seen six pieces of meat we share, the child two, the mother two then I eat the rest’.*

*‘ It depends on the amount of meat we have at home, my family really love meat. My wife loves meat and my children love meat but I don't. As soon as they boil because they first boil them, every child immediately hurries to get me the soupe, hahah, so I left that for them in other words, but in general my wife is the one who eats the most and the children because I don't like it’.*

*‘ Giving an example of myself,I don't like meat but my wife likes it and also the children. So when we cook meat at home, though most of the time I am not at home .They make sure that they eat it and get satisfied . Because I know the amount of meat I buy , I buy like 1.5 kg or 2kg. All I know is that when the meat is ready to be eaten my wife eats first right away, I can't eat more than one or two or three meats. When I see that they have eaten, I feel happy and my family when they are full, I feel happy’.*

*‘ You do not know how many pieces are in one kg of meat. I like to buy one kilogram of beef and pork. So when they are preparing it I can not know that this child is only eating a large portion, the child and his mother eat a large portion and I am there waiting to be served. The only thing I know is that they give me a sauce with meat to eat with my fufu’.*

*‘ Usually us men like to be moving a lot, often you don't even get to eat at home. Sometimes you eat at work. But when you have bought some meat you leave the preparation in your wife’s hands. Sometimes they even finish them all. Most of the times when the mother leaves their families it does not turn out good ,because only them know how to distribute food to her family. I am just responsible for taking care of my family and then get served as she has planned”*

*‘ I will not go far from what others said , especially because men have less time at home, but let's say that maybe I spent the whole day at home and bought some meat. My kids are a bit mature. When they are from school they know we are cooking some , so they do the cleaning, do their homework and then wait for food to be ready. You can't say whether you eat too much or too little. Only when they prepared Isombe did my wife give me the biggest piece of meat in it hahaa.*

*‘ like my child when he is sick, I often feel like preparing soup for him/her. That is in case I can not afford to buy some meat whereby now one kilogram costs around five thousand five hundred Moderator: A pound of meat?*

*‘ The beef bones cost three thousand and the Roti cost Three thousand five hundred. So talking about myself, when on that day I am not afforded to buy them , I buy dry fishlets and boil them with some tomatoes and make a good soupe for my kid.That's how I do it in my capacity. I feel like the kid needs some hot soup to help him/her*

*‘ First of all when the kids get sick, the first thing I do is to take him to the doctor or see if there is something a community health worker can help me with. You see, sometimes one can get sick while he/she has the appetit ,so in that case I will not bother looking for meat. We just make sure that he/she eats a balanced diet.let's say in our family, sometimes you can also prepare potatoes with a dry fishlets sauce and get some milk to drink, then from there you get a complete meal.’*

*‘Yes, I am number eight, a sick child is really special. Back when I was young I always wanted to be sick because the kid was treated differently when sick. They could buy that meat to make a soupe for a sick kid. In my family we try our best to treat that sick kid well by finding his/her favorite dishes that would help him/her.Thank you’*

*‘ When a child is sick, I take him to the health center. Whenever I see that he has a high fever or any other changes in his/her health my wife and I immediately. Another thing I do is follow up on the medicine he/she is taking as prescribed by the doctor until I see that he has regained his strength and is cured’*

*‘ eeh for me it would depend on how i see the sick kid . I was privileged to have my father as a doctor, I always run to him when my kids are sick to seek help and sometimes he gives me some basic medicine. If he cannot help for the moment I immediately go to the hospital and they help me’.*

*‘ I am also number four, I live next door to a Community Health Worker, when a child has a problem, I go first and consult him, and tell him how the child is feeling if he/she got a fever etc. Before I would use a wet cloth to cool down the kid with high fever . But he advised me to not do so, he reminded me that that could down yes the fever but it won't treat him from what he is suffering from. Please bring him to be for consultation or if I am unable to do so I will transfer him to my fellow community health worker, ‘.*

*‘Our mother used to do it for us back when we were young , and waited for the morning to go get the herb medicine where we lived. We had it and it was a herb medicine called IBISABANYAMA , it was used to treat Malaria. For we no longer use the herb medicine we take the kid to the hospital because only the wet cold towel cannot help it just cools down the fever not the actual disease’*

*‘ Number eight, the help we give the child before taking him to the doctor, depends on how he/she is sick . If his symptoms include a high fever, you can washhim, give him/her some water to drink, or put a cold cloth on him to reduce the fever by then take the kid to your Community health worker or take him/her to the nearest heath center but most likely we are recommended to bring them to the community health workers near us. Thank you’*

*‘ It is the government's plan, the way the system is built today, where we live in community assemblies, in communities. When we meet they make sure that they inform us about it.’*

*‘ yes, they always remind pregnant women to not forget to do the HIV test within three month.They update us about when our children will be vaccinated , in short that’s where we get the information we need. We have basic medicine,the community health officer gathers us together.’*

*‘ Yes, here every village has a community health worker , I won’t go far from my friends, But what I can do is ,if your kid get sick immediately go to the community health worker for some help and advice. When one is unable to help the sick they will transfer you to his colleague for help. If all of them cannot help you then go to the hospital to see the doctor. Do not treat your kid with the herb medicine, because you cannot really k now the quality of it. Back in days they use to give us a cup of banana beer and that would treat us malaria or they would give you the herb called KIMBAZI which also treated malaria.haha I don't know if todays malaria is different from what we use to get Moderator: Now, about malaria, could you explain to us the things that malaria was giving to people suffering from malaria.participant: They took it as a stick and twisted it, took it out and the disease came out.’*

*‘ Other than that, people get civilized day to day due to the radio news. Everyone has a radio in their phones .For The leadership nowadays is close to the people from the small units known as ISIBO, the communities. Everything is explained by the community health workers. They go to people's homes and explain what they should do when their children are sick. What was different before the first thing they had to do was to reach out to traditional doctors to get herbal medicine which was called BAMBUWA. I guess some of you know it ,itr was used to treat fever participants: We know’*

*‘ Yes, they had to rub that herb and mix it with a hot banana beer to treat you from malaria. From there you get back to normal’*

*‘ It ends for real. You would go to bed once they gave you that BAMBUWA that was on fire. You would immediately recover’*

*‘ Hot banana beer hits hard and gets you tipsy and then sleep you wake up feeling good’.*

*‘ What he said is true but where I grew up those herbs really traited but for a short period.I had been used to getting sick after a certain period , like some people knew that every summer they had to get malaria . But ever since I started to use the medicine for Malaria it has been twenty years without suffering from it’.*

*‘ I am number seven, we can get good drinking water. In my family. Because we still can not afford industrial water or bottled water, we boil water, we keep it in a clean small jerry can. We do that everytime we finish that jerrican’.*

*‘ Well, whenever I have time I can fetch or maybe my kids or my wife it depends on who is available have time’*

*‘ number eight. When it comes to clean water, people perceive clean water in different ways. Some people consider tap water as a clean water to the extent they drink it ,and others boil it before drinking . That is what my colleague here does. In general people of this village get clean water , which is tap water. So talking about drinking water, not everyone boils it. Even in my family it is not every day that we cook it but sometimes we do’*

*‘ No, it was not there before because most of the people here used to fetch water from swamps cause this sector of Masaka used to always have the Cholera except us who migrated from other villages. We are very thankful for our government and investors who brought clean water to our neighborhoods.In general, we are getting clean water slowly. Though like in my neighborhood we still have that issue of missing clean. We all know that we get tap water on Wednesday and it runs out on thursday. So we make sure that we use that one we kept in the jerry cans well till the next week on wednesday. That’s how it is here’.*

*‘ Yes we all drink tap water: but we no longer go to the swamps’*

*‘ I'm number four, most people have bicycles they use them to curry fetched water but it is not even far off. When we are running out of water in our cell we just go to our neighbors . for those with no bikes they just use other’s’*

*‘ For example in our village it is a must to have,we all got the toilets. Even those with no toilets are followed up and given the help. So for the good toilets I think they should be covered , with the roof ,paved and with a door. Knowing the period we have been having them would not be easy to remember but it is around five years. Before you would find out that some hava them but with no door or paved with only woods but as the country develops everyone has a toilet in his home’.*

*‘ I am number five.we have the toilets and I guess nowadays everyone has it. Though you can not tell the number of people who own them, for sure most of them own them ,and they are well built;with the roof closed’.*

*‘ I am number one, I will not go far from what my colleagues said because I live in this village too. But we have the toilets ,according to people's financial ability though we still got a few who has no toilets ,i think we should be honest and speak for the rest’.*

*‘ What I mean is, yes we have toilets and they are well paved with roofs ,closed and covered and they do not realize the insects but cause diarrhea’ .*

*‘ yes it is paved and coved and closed’*

*‘ I guess they are simple ways to cover them not big deals’*

*‘ You just take flat wood and then leave a space for the window. Then people can use it’*

*‘ the problem with mine it is not covered’*

*‘ yes i am number 8 the reason why they sensibilized the building of the toilet and paving them was to get a way of cleaning them’*

*‘ yes , as i told you since when the community health workers came in they always check on us if we have clean toilets and they teach us how to keep them more clean’*

*‘ there are the effects of course because even that time you take going to fetch could be used to do something elses which is necessary too plus the energy use too’.*

*‘ So you see now , we are developing very quickly, these kids got a thing they poop called potty so they use them and they mothers go to throw that waste in the main toilets . even those who can’t afford them they just poop on the side of the main toilet and their parents just clean it up and put the waste in the main toilet’.*

*‘ Children do use the same toilets they use the potties ,and then parents clean them up’.*

*‘ The challenges are there, remember i started telling you that here in our village we only get tap water once in a week so you have to be conscious about how you are using that one you kept until it is back. That itself is a barrier to cleanliness’.*

*‘ Yes the challenges are there,because people from this village do not get tap water on time ,as they need it yet we know water is key to cleanness. That comes first then soap after. Though we try our best to get it, that limits us to be full clean. Like now one who would wish to do the inhouse toilets or bathroom would be very challenged if they have no tank at home because these flushing toilets require much water to flush every time. So that’s a challenge’*

*‘ The challenges are there of course but you try your best to overcome them. If you have like three jerry cans you have to make sure that you use them properly’.*

*‘ Because of the low rate on which we get water ,as my colleague said. It limits us from doing some things related to cleaning. For example, I had the kitchen garden at home but irrigating it is a problem on sunny days. But we have a shortage of water. Otherwise we would irrigate it maybe twice a day, morning and evening so that we can easily get those vegetables otherwise it won't be easy to get them for yourself. If we had a good flow of tap water we would know how to program yourself and plan how we use it. Make a plan for what we use in the bathing room and the rest for the cleaning. It would really helpful if we get it on time’*

*‘: I think they should do more sensibilisation and tell parents what they should do to improve the wellbeing of their kids’.*

*‘ For me I think they should sensibilise pregnant women and parents on how they should take care of their children and that should be done like twice a month because less than that they would easily forget what they taught them’.*

*‘ what my colleagues said is all, there should be a sensibilisation. Like us we got privileged. We had this discussion, so that should be a thing and they train pregnant women how to take care of them and parents how to take care of their kids. The trained parents would train their fellow parents’.*

*‘ the first thing is to do the mobilization in parent. Literally we have food but we do not know how to manage or prepare it. How can you have eggs and then sell them just to buy sweet potatoes or banana beer? We have community health workers who are in charge of the wellbeing of the society. They were trained how all that is done. Having clean houses, dishes ,toilets, and preparing the kitchen gardens. So for sure the mobilization is really necessary for them to know how to take good care of their children as we have been talking about it. Like now us we have something new to share with our fellow parents’.*

*‘ there is something that crossed my mind , you see most of us have kids that are still below seven years old. If you have like three kids one is Five ,another three and maybe another One. I think we need Early Childhood Development schools that are affordable and near us in our villages. U see in the regular early childhood development schools they pay two thousand per kid and if you have three kids that makes four thousand per term. I think it would be better if we had affordable schools for our kids to attend too’.*

*‘ Yes we have them, but most of the time you find them far from where we stay. So it is not easy for us to take them there every time while we have to go back and go to work. It would be better to have them near so that in the first days you can accompange them but the place where they can take themselves there when you did not get time too’.*

*‘ Protective food includes green vegetables and animal derived food’.*

*‘ Energy giving food includes sweet potatoes, Irish potatoes, and cassava’.*

*‘ A pregnant mother should eat green vegetables, eggs, and she should eat foods which fall in the other three classes (i.e. energy giving food, body building food, and protective food) as participant 3 has mentioned’.*

*‘ Thank you, a pregnant mother should eat tuber crops, green vegetables, eggs, and meats. When a pregnant mother uses these kinds of foods properly, it is beneficial not only for her own health but also for the fetus’s health which is in her womb’.*

*‘ Especially a pregnant mother is supposed to eat fruits such as tree tomatoes and passion fruits, tuber crops, and animal derived foods because they are beneficial for her health and that of her fetus’.*

*‘ A pregnant mother should eat different fruits like ripen bananas. She should also eat tuber crops and meats because they are good at the fetus in her womb in terms of growth’.*

*‘ A pregnant mother should avoid tobacco smoking, alcohol consumption because they harmful to her and the fetus in her womb as the healthcare providers have educated us’.*

*‘ Regarding the type of food that a pregnant mother should avoid, for my side, I would not feed my pregnant wife the sweet potatoes and Irish potatoes more frequent per week. Maybe because of someone’s economic status, I may feed her at least one sweet potato per week, then I give her green vegetables, milk, rice, meats, and fruits. Yeah, I should feed her those kinds of food which will make her and the fetus to stay healthy. If you are unable to afford the cost for meats, you feed her the littles fishes’.*

*‘ Thank you, regarding the type of food that should be avoided during pregnancy, I think a pregnant mother is not prohibited to consume any kind of edible food. Instead, what matters is the hygiene by which that food was prepared with. There is no kind of food that is avoided during pregnancy in case it is edible because the food for the pregnant mother is prepared in respect of the balanced diet which is made up of energy giving food, protective food, and body building food’.*

*‘ My additional point is that a pregnant mother should eat a well-prepared food which is made up of carrots, green vegetables, and tuber crops. Thank you!*

*‘ Especially, a pregnant mother should avoid alcohol consumption and tobacco smoking because they are very harmful to her and to the fetus in general’.*

*‘ Those kind of beliefs are present but I don’t think that are only applicable for pregnant mothers but also they are applicable for everyone depending on which denomination does he/she belong to. For instance, the church members of 7th day Adventist Church, there are kind of meats which they are prohibited to eat depending on their doctrines’.*

*‘ They prohibits the consumption of some kinds of fish, and the meats of pig. That’s what I know’.*

*‘ Here in the village, those beliefs are present because a mother may say that the pig’s meats have no vitamins. Indeed, this is true because people say that pig’s meats have no vitamins compared to cow’s meats’.*

*‘Regarding the type of foods which are prohibited to be consumed due to beliefs, the 7th day Adventist prohibits the consumption of pigs and fishes without scales. They also prohibit the consumption of animals with no divided hoof such as rabbit and ducks. Yeah, those are the foods that are prohibited by those churches but I think it would be necessary if the members of these churches which prohibits certain kinds of foods, take into account of the consumption of acceptable foods such as chicken, goats, eggs, and fishes with scales because they are rich in nutrients that build the body’.*

*‘ what I want to add on is that there are kinds food that are prohibited by beliefs depending on one’s denomination. As my colleagues said, most of the churches prohibit the consumption of those fishes without scales but if I leave them behind and eat little fishes, fishes with scales, eggs, cow’s meats, goat’s meats, and chicken as they are not prohibited, my pregnant woman will also be healthy. Therefore, the way I take care of that pregnant mother, is the same way that I feed those children with food rich in nutrients so that they are protected from getting stunted. To cut in short, the most prohibit food to be consumed according to beliefs are those fishes without scales and ducks but it all depends on one’s denominations otherwise there are other churches which consume them’.*

*‘ As we have seen, there are beliefs that may prohibit a pregnant mother from eating certain types of food, such as pig's meat, fish without scales, and duck's meats, which are still beneficial to the pregnant mother's health, as my colleagues mentioned. Nothing can stop me from preparing that food for my pregnant wife if I am not a member of the denomination that forbids those foods’.*

*‘: An infant should be breastfed for six months exclusively. After six months, that’s the when the complementary feeding is introduced’.*

*‘ Normally, the reason is that it is harmful to the infant’s digestive system once the complementary feeding is introduced before six months of age’.*

*‘ Normally, a newborn should be put on its mother’s breast immediately after birth to maintain its body temperature. Then, the following is the exclusive breastfeeding up to six months of age’.*

*‘ My suggestion to this point, an infant should be breastfed exclusively for six months because he/she is eligible for complementary feeding after that period. Otherwise, from birth up to six months, he/she is dependent on his/her mother’s breasts and he/she should be breastfed whenever he/she needs it’.*

*‘ An infant should be breastfed exclusively for six months, after which complementary feeding should begin with fruits and porridge. That is, when an infant is introduced to those fruits and porridge, he or she will not be easily stunted, but when he or she is first introduced to solid foods, he or she will be stunted at a young age’.*

*‘ Fruits such as passion fruits and ripe bananas are used to begin the introduction of complementary feeding. You can also make a mixture of mashed banana, green vegetables, and grinded flour of small fishes that an infant can easily suck and swallow. At that age, an infant is no longer satisfied solely by the breast, which is why complementary feeding is introduced’.*

*‘ normally an infant is allowed to be given complementary feeding at six months as they have mentioned. When he/she at below that age he/she is not able to take that complementary feeding but when he/she is above that age, the breast alone is no longer enough to satisfy him/her, which is why the complementary feeding is introduced staring by fruits, green vegetables, and porridge’.*

*‘ An infant should be breastfed for six months exclusively. The reason for not starting complementary feeding before that age is that the infant's stomach is not ready to digest food other than breast milk and his/her esophagus is too narrow to allow food to pass through it, which is why semi-solid food, mashed food, and porridge are introduced first’.*

*‘ The complementary feeding should be introduced, starting by fruits like ripen banana because the infant’s stomach and esophagus are still weak to deal with solid food. As the child grows older, you start to introduce solid foods depending on the child’s age.*

*‘ My additional idea is that, an infant at six months of age is fed soft food like biscuit, pawpaw, ripen banana and that egg’*

*‘ An infant introduced to the complementary feeding, I think he/she is allowed to be given every kind of food instead what should be cared of is the preparation. During preparation, the foods should be smashed so that it will be easier to swallow and the responsible person should keep in mind the inclusion of all the other three food categories (i.e. energy giving food, protective food, and body building food) in preparation of the child’s food’.*

*‘ as a compliment to my colleague, normally, at six months of age, the food prepared for the infant should consist of energy-giving food and protective food. Instead, as a responsible person, I must maintain hand hygiene and utensil hygiene during preparation; otherwise, the child's health may be in danger if my hygiene is poor during preparation’.*

*‘ Thank you, what I think that is prohibited to be fed to the infants are cassavas, sweet potatoes and meats. Meats are prohibited because they are difficult to chew for an infant who sucks the food given to him/her. Therefore, I believe that foods such as meats and sweet potatoes are prohibited because the infant cannot swallow them as easily as porridge and breast milk’.*

*‘ Normally, there are different kinds of complementary feed given to the infant but it depends on his/her age. An infant at six months is given milk, green vegetables, and tree tomato. Therefore, as he/she grows older, you start to introduce solid foods but respective a proper preparation’.*

*‘ Cow's milk is generally beneficial to the health of all age groups, not just infants. Milk is available in our village, but infants are not given coagulated milk; instead, they are given fresh milk. Infants in our village are given fresh milk that has been well prepared and stored in a baby bottle to make feeding easier, but coagulated milk is not given to the infant because his/her stomach is not yet ready to digest the lipids in it’.*

*‘, it is not necessary to give cow’s milk to an infant once his/her mother’s breast are providing breast milk enough to satisfy him/her. Instead, the complementary feed that I may provide for the infant are fruits, green vegetables, and other kinds of foods prepared in the way that is easier for the infant to swallow but I think cow’s milk is not good for an infant once his/her mother’s breast milk is enough for him/her’.*

*‘ It is because there are teachings that we hear and they show that it helps the infant but it may be depending on that the infant is not satisfied by his/her mother’s breast milk. Therefore, in case the child is satisfied with his/her mother’s breast milk, it is not necessary to give him/her because that it may be harmful to the infant’.*

*‘ My suggestion differs from this man's in that milk is beneficial to people of all ages. It is not necessary to state that milk is only given to infants who are not satisfied by their mothers' breast milk; all infants, regardless of condition, should be given cow's milk; however, I would like to discuss what I feed my children at the start of complementary feeding. I usually make sure to include porridge flour, sugar, and fruits like passion fruits, oranges, and ripe bananas. I feed them, beginning with soft foods and progressing to solid foods as they get older’.*

*‘ You did ask if we get milk at our homes. To cut in short, cow’s milk is not easily accessible because the cost for one liter is high compared to our economic status. Our children have recently been able to access milk in newly introduced ECDs; otherwise, we can only get porridge because we have a good harvest of maize; if you are lucky enough to get milk that day, you mix it with the porridge and then give it to the child. Thank you’.*

*‘ I also have a child under the age of five, but because of our economic situation, it is difficult to get milk in our homes, so we feed our children porridge, which is easily accessible. Even the other fruits we mentioned, such as tree tomatoes and passion fruits, are difficult to obtain due to their high price. I believe porridge is good for a child's health because it is easily accessible, but alcohol should be avoided because it is harmful to a child's health. Otherwise, a daily liter of milk would be too expensive to afford, but our children could drink milk if Gikuriro provided it’.*

*‘ It is okay to give cow's milk to a six-month-old infant, but it is not okay to give it alone. Giving the child a mixture of milk and porridge, in my opinion, is the best option because you will have provided enough nutrients’.*

*‘ Since the child began eating fruits at six months of age, the following foods to feed him in his first year should be semi-solid foods such as green vegetables and mashed banana because you can't give him cassavas or posho and expect him to benefit from them. The child should be fed according to his age, and he should not be fed frequently; instead, he should be fed at specific times, and the food given to him should be fresh, not cold, because that's what brings stunting or intestinal worms or kwashiorkor. Therefore, when my children are six to seven months old, I feed them green vegetables, grinded small fish, and mashed bananas, so that anyone who sees them says that they eat a balanced diet’*

*‘ Normally, by comparison a child is like a domestic animal. Therefore, I think a child should be fed every hour if you able to provide that food per his need because a child who is being weaned is always willing to eat’.*

*‘ What I think that should be fed to the child in his first year include vegetables, small fishes, carrots, beets, and porridge. Then, the child should be fed five times a day’.*

*‘ You should prepare food made of fruits, ripen banana, fish, small fishes, and banana for a child in his first year. On the other side, it would be awesome if he also makes it to access milk except that it difficult to get it here in our village’.*

*‘ The prohibited foods to be given to the child in his first year are those foods which are difficult to swallow such as cow’s meat, goat’s meat, and pig’s meat. Otherwise, he can be fed on a well-prepared fish and its soup.’*

*‘ Depending on my financial situation, I train my child in his first year to feed on the same food that we do. In his first year, I make sure that a flour of small fishes is present, as well as amaranth vegetables (dodo) because I have a kitchen garden, and I also make ensure that if the available banana is insufficient to feed all of us, the remaining portion is reserved for that infant and prepared in a separate saucepan, as agreed upon with my wife. If it were possible, I would not be counting the number of times my child has to eat per day because I would prefer that he eat whenever he needs to so that I do not see him hungry, but due to limited finances, my child must eat at least four times per day. Not only taking food but also porridge should be there as a compliment but training him to feed on what we are feeding including the other cassavas and sweet potatoes that we were avoiding with the exception of certain types of meat, such as the abdominal ones, which I believe are not okay.’*

*‘ They say that an infant who is fed abdominal meats becomes dumb (loughs). As a result, I feed my child what I and my wife eat because I can't go stealing or borrowing money to feed him unusual food.*

*‘ at my house, a child in his first year is fed the same food as the rest of the family, but it should include body-building, protective, and energy-giving foods. I then prepare food for him that includes vegetables, tuber crops, cereals, and when he is still in his first year, I assume nothing is prohibited to be fed to him, but rather the preparation is to be mindful. Food preparation for children should differ from that of adults. When it comes to tubers, they should be mashed so that they can be easily swallowed, and when it comes to porridge, it should be made of maize, sorghum, and soya bean flour because this mixture is nutritious, and they taught us that porridge with soya is similar to porridge with milk. Therefore, I believe the child will be healthy even if you prepare the type of food we usually eat if you do so with proper hygiene.’*

*‘ The number of times a child must eat per day in his first year depends on his health or appetite, but at my house, I may feed my child four times a day because we know that a child must eat breakfast, which should be fresh cooked food, lunch at noon, and food between lunch and supper. But for a child who has high appetite may go beyond that number of feeds per day. Thank you’.*

*‘ Normally, a child shouldn’t be fed below four times a day. He should be fed in the morning at 7:00 am, at 10:00 am, at noon, and at 16:00.’*

*‘ alcohol should be avoided in children during their first year because they are not yet able to consume it, even though some parents believe that giving alcohol to children at a young age helps them fight intestinal worms. It is also not acceptable to smoke in the presence of children or in a crowd such as this one. Yeah, my point about the food that we typically consume is that, as my colleagues mentioned, those are the types of foods that we typically consume in our village. As previously stated, we eat tubers, which provide energy, as well as protective foods such as vegetables. My view about our children's stunting is not one of scarcity of food, but of how it is prepared; therefore, when I leave here, I will educate my wife that whenever she is preparing food, she should ensure neat hygiene and smash that cassava for our child if that is what we are going feed on’.*

*‘ In drinks, alcohols are avoided to be given to children in their first year as they have mentioned but in food, I think nothing I should avoid depending on what I am feeding on. Maybe cassavas, yams, and sweet potatoes should be prohibited during his first year but because that is what his home is feeding on, he gets used to it slowly by slowly. You train him by mixing with banana, ripen bananas, egg, and fruits until he is able to eat the whole sweet potato. I think the only prohibited consumables are those alcohol and tobacco as they have mentioned, nothing more’.*

*‘ I won’t go far from what my colleagues have said, normally a child is neither allowed to drink alcohol nor his parent is allowed to smoke in his presence. I think that’s it, otherwise, the child is allowed to feed on the rest of food kinds because if we have said that a child should eat a balanced diet, you should provide it to him’.*

*‘ Normally, children should be served first as soon after the food is ready because they easily get hungry. Coming back to a pregnant woman, she is much like that child, therefore she should be served as early as possible so as to maintain her health and that of the fetus. As we farmers, we usually take our lunch around noon or 13:00 pm, therefore, you can’t let a child wait that long because that’s when the problems of stunting come in due to late feeding. Children and pregnant women are prioritized during meal serving so as to safeguard their health’.*

*‘ From my point of view, a child should be the one to be served first because they are fragile and they can’t wait until when the food for adult will be ready. Their food should be prepared earlier and put in a pan that will keep it and warm and there should also be a flask of porridge so that whenever a child is hungry, the food is also available’.*

*‘ At my house, I never eat before my child; if I buy something, he should be the first to feed on it. If my wife prepares the food at my house, she must ensure hygiene during preparation and then serve the child first. Then, when the child has finished eating, we are the next ones to be served, and the other child will be busy playing because he has finished eating. Furthermore, we farmers bring food with us to the field so that the child has food while we are cultivating, and when we get home, my wife first takes care of the child's feeding before thinking about us adults’.*

*‘ Regarding the quality of food we serve to children, at my house, I avoid serving food cooked the day before to my children because there are people in charge of health in our villages and they educated us that the food cooked the day before has lost quality because serving it to the child may cause him to suffer from diarrhea, deteriorating the child's health. Therefore, the food varieties for children that are available in our district include bananas, Irish potatoes, sweet potatoes, tomatoes, and carrots. To maintain the quality of food prepared from all of these different food varieties, we as farmers make sure that when we go to the field, the food is kept in a well-covered pan, and if it is porridge, it is also kept in a flask, so that when it is served to the child, its quality is still preserved. So then, the available food varieties here in Mutenderi are sweet potatoes, bananas, cassava, tomatoes, and carrots. When people are willing to serve their children, all these food varieties are available, thank you!*

*‘ Normally, the point of quality is evident when you handle and prepare something with hygiene. That is, if it is food prepared for a child, the responsible person should maintain hygiene, whether it is on the clothes or on the hands, and whatever you touch should be kept clean. Most food varieties are available here, with the exception of soya beans, which are not grown widely in this region. Thanks!*

*‘ in terms of the quality of food served to children, as my colleague mentioned, the first thing we consider is hygiene during preparation, in which the responsible person first thoroughly washes his hands and, if the food he is preparing requires washing, he/she does so. Not only that, but he must ensure that the utensils that will be used during serving are thoroughly cleaned and dried, because you may have prepared a balanced diet, but the child's health is adversely affected due to poor hygiene of the utensils used during serving. So, I consider hygiene to be the most important consideration for the person preparing the food, for the food, for spoons, cups, and plates, and this is what we do at home. Then, among the food varieties available in this district, there are tubers such as cassava, yams, and sweet potatoes, as well as cereals such as maize, sorghum, and soya beans in small quantities. So, I believe that when all of these are prepared with extra care, with an addition of animal-derived food and vegetables, the child's health prospers’.*

*‘ Domestic conflicts have a significant impact on children and pregnant women because when a child under the age of one is born during a conflict, he is severely traumatized. First, he notices that his parents do not love each other, which causes him to lose hope that his parents love him. Second, we've been taught that whenever a pregnant woman is upset or made angry, the effect is transmitted directly to the fetus in her womb. For example, a pregnant woman may encounter a frightening life situation during her pregnancy, so that when the child is born, he may live a life of depression or rage because of what happened in his family while he was in his mother's womb. As a result, I consider domestic conflicts to be a threat to the health of pregnant women and children’.*

*‘Domestic violence usually has a negative impact on a pregnant woman because it is said that when you harass a pregnant mother, even if she has just conceived yesterday, the baby in her womb suffers because he is automatically connected to the external environment. That is why conflicts, harassments, and exhausting tasks should be avoided by pregnant women, as they say that the baby will grow poorly in his mother's womb. Women should be treated well during pregnancy, not harassed or made angry, because that is when the baby will be born with a great hope of living because his mother was treated with care while he was in the womb. After the child is born, he should not be told harsh words; instead, as his father, you should be there for him, take care of him, and provide for his needs both in the presence and absence of his mother. You must feel free to be near them so that they realize that their father is there for them and if he is playing with his siblings, it is okay for you to join them. Otherwise, when a child is always treated harshly, he loses concentration and does not grow well’.*

*‘ Normally, domestic violence adversely affects the family. We are free to express our opinions because you have a colleague, that gentleman, who has come to brief us before joining this discussion, showing that we are now one. Domestic conflict is terrible because it affects not only family members but also rodents like mice because when you have a disagreement with your wife, she leaves and the other rodents are nowhere to be found (the whole group loughs). So, when a woman is treated harshly during her pregnancy, you will notice that her child is always enraged, and when he grows up and starts school, he is always fighting because nothing good came from his family while he was in his mother's womb. So, when there are conflicts in the home, many things suffer: the family may not develop, the children may not concentrate in school, and it traumatizes the person who is being violated’.*

*‘ I'd like to share a habit that I inherited from my grandfather: when I buy meat, whether it's fish, cow's meat, goat's meat, or pig's meat, the wife cooks it and places it on a plate where everyone in our family, including the other little one who was with me earlier, takes the portion of meat that he is willing to take. Then, after everyone has served himself/herself, even if the quantity has been small, no one can claim that others consumed a lot more than what he/she has eaten. So that's how we do it at my house; there's no such thing as saying this portion is small while the other is big’.*

*‘ Normally, it has been two years since I began eating meats, but during the fifteen years of my marriage, I used to buy meats for my family members, and they ate without any problem. Since then, my wife has continued to prepare them, and when they are ready, she places them on a plate for us all to share, including our three sons, so that no one will complain to one another that he has eaten too much. So, due to my health issues, pig's meat is the only kind of meat that I eat, but when we buy meat at home, we can't say that someone eats a large portion’.*

*‘When we buy meat, we all share a plate, but a child under the age of five is served on a separate plate because he is not yet able to eat like adults. That child requires extra care at home, so if it is meat, his meal is prepared in a separate saucepan and dissected into small portions to make it easier for him to chew and swallow, but we adults share the same plate’.*

*‘ When I buy meat, my wife is in charge of preparing it and taking full responsibility for reserving for a young child if we have one, and if the food was not enough to warrant reserving, she is the one who manages that. Then I can't say that I have to manage food while also having a wife; that's impossible’.*

*‘ Regarding the meat issue, a parent used to bring a 1kg of meat and say that a quarter of it was for children and the rest was for parents, but that no longer exists because, as my colleagues mentioned, food is shared equally on the same plate’.*

*‘ To add to what my colleagues have said, these things usually happen in men because there is a time man may buy meat, he cuts it into small pieces and counts it to his wife when he gets home. When the woman cooks them, they shrink in size so that she may be worried about serving part of it to the children before serving the husband if he is not around, so she keeps much of them in a pan for his husband so that he will be the first one to feed on the meal he has brought. Most men do not eat like women; there are even some men who look at the time when the food will be ready and then leave so that their wives will reserve food for them. If the wife has reserved a small portion or a few meats, this is one of the ways conflicts arise. On the other hand, there is a separate part where men are given a large portion, but in general, when it is meat, all family members share on the same plate with the exception of a child under the age of five, who is served on a separate plate’.*

*‘ normally, a sick child's feeding is determined by his appetite in that situation; even though we are poor, when a child is sick, we ask him what kind of food he would like to eat. There may be times when he may suggest a type of food that you do not usually eat; in that case, we as parents must do our best to find that specific type of food. Most of the time, we don't eat meat in the village, so if a child falls sick and expresses a desire to eat meat, we must find a way to get those meats. He may also suggest a type of food that you are unable to afford at the time; what you do is reassure him and tell him that you will buy it for him once you are financially stable. It’s in that case, we help our child when he is ill, as his mother does. If the mother approaches the child, she must go through the same process to and what type of food the child is willing to eat, and if we can afford it, we do so; if not, we reassure him with a kind heart’.*

*‘ When caring for a sick child, you ask him what kind of food he needs to eat. He might be willing to eat rice, bananas, or Irish potatoes. In that situation, you look for ways to get that food, and if you can't in that very time, you reassure him that you will provide it once you can’.*

*‘ In addition to what the previous participants have said, everything should be done with a kind heart because everything changes not only for a child but also for every single person who is sick. His mood and feeding pattern change, which is why he must be approached with a kind heart because he may be willing to eat food that is beyond your ability, which must then be replaced by another type of food. Therefore, it is very important to approach a sick child with a kind heart in order to maintain his feeding pattern by respecting the type of food that his appetite is driving him to eat. That's all I have to say. Thank you’*

*‘ First of all, if the father was not always at home and the children spent a lot of time with their mother, the father should be present when the child falls sick and be the one to take him to the clinic. In that case the child will feel his father's presence because children desire different foods and drinks when they are sick, such as juice, milk, or other snacks, so that once the father has taken the child to the clinic, he will be able to provide whatever the child desires to consume. As a result, the presence of his father will play a very important role in his recovery’.*

*‘ When a child falls sick in the village, we usually take him to the community health worker, who may prescribe medication depending on the child's illness, but if he doesn't have any, he refers you to the health center for further management’.*

*‘ The first thing you do for a child whom you think that he is sick, is to ask him thoroughly about how he is feeling and do the assessment by asking him how every single part of his body is functioning because that’s when you will suspect the disease that he might be suffering from. For instance, he may state that he is having fever, in that case you palpate and feel whether it is true. By doing all of that, the child feels confident that his parents are there for him and then you take him to the healthcare provider for consultation’.*

*‘ My point is that men are not always at home, but when a child becomes ill, a man should be the first to make time for that sick child. So, once we realize the child is sick, we take him to the community health workers first. Then, because community health workers are trained, they proceed with their assessment and prescribe medicines based on the findings. However, if the prescribed medicines do not work, we return the child to the community health worker, who then transfers us to the health center for further management. Therefore, in general, I would like to emphasize the importance of treating a sick child with kindness and sparing some time for him, as this makes him feel loved. For example, just a few moments ago, I was with my young child here, and perhaps he expected me to buy snacks, and I did, which means that we have formed a strong bond between us, and the next time I mention going out, he will be willing to accompany me. By cutting in short, the community health workers are the first healthcare providers whom we consult first when our children fall sick and then they transfer us to the health center once they are unable to treat the children’.*

*‘ Those things of giving other kinds of treatments to the child before taking him to the health facility used to exist a long time ago, where if a child was suffering from malaria or abdominal pain, they would take him to a witch doctor, who might even receive someone's properties in vain. Our forefathers used to use herbs for treatments when they or their children fell sick, but nowadays the healthcare system is decentralized, and we were educated that we must immediately take our sick children to the community health workers, who conduct tests and, if they are unable to treat the child, transfer us to the health center’.*

*‘ For my side, when a diseased child can express how he feels, I first approach him and ask how he feels and point where he is feeling but there are other children who are not able to express their feelings, most of the times, those are cared by their mothers. To be honest, what I wanted to share that was different is that here in the village, there are some conditions that we don't take seriously, such as cough; in this region, we all know that before taking that child to the community health worker, we try some herbs known as "Umuravumba" in our local language. That coughing child is then tried on those herbs, and when they fail, he is taken to the community health worker because they have started to suspect that the cough is caused by a serious condition such as pneumonia. To be honest, that’s how even my children are treated’.*

*‘ Normally, everyone in our region is aware of that. Because the majority of the herbs used are grown near our homes. So then, I would not say that home treatments do not exist; they do! On the other hand, we go to the community health workers and health centers, but we also use traditional herbs, if I'm being honest’.*

*‘ Such things do exist! Starting by myself, regardless of the disease that my child maybe suffering from, I try medicinal herbs before taking him to a community health worker or health center. When they fail, that's when I take him to the community health workers or the health center. That is something that we all do’.*

*‘ The medicinal herbs that I usually use include what we call “igicunshu, ireke, Umuravumba, umwenya, umuyenji, and umubazi” in our local language. There is also atime when we use a mixture of umuyenji leaves, mango tree leaves and they are cooked together where that mixture is called “Ibisabiko” in our local language.*

*‘ They were used to treat me by my parents, so I inherited them. Since they were used to treat me, I use them to treat my children, but when they fail, I take them to the community health worker’.*

*‘ If you look closely, you can see that these home remedies are derived from Rwandan culture because traditional healers used these medicinal herbs widely a long time ago. So, we grew up being treated by our parents using these herbs, even though some of them have been banned by the government, while others, such as the previously mentioned "isonga and umuravumba," are still in use. So, these herbs are then used in conditions which are taken as simple ones but the serious ones, we take the child to the community health worker first’.*

*‘Furthermore, we have seen two places where sick children or adults are taken for treatment, but people also go to pharmacies because health professionals work there as well. Most people go there because they are ill and do not have health insurance’.*

*‘ True, we have access to safe water in our living places, but the cost of one jelly can is extremely expensive at thirty Rwandan francs. In that case, we continue to use traditional water sources because the cost of providing enough water for the entire family is too expensive. So, you can't take one jelly can in a household of eight people and expect to use it for cooking or washing clothes; some activities are postponed, or we go to the swamps to fetch water that is truly dirty. In general, we are still facing the problem of safe water because we continue to drink swampy water, which may be a source of intestinal parasites’.*

*‘ We still have a problem with safe access because some households are required to walk a one-kilometer distance, which is incredibly challenging. In that case, you'll notice that these individuals are heading to the nearest water sources, which are the swamps and ponds mentioned by my colleagues. So, it would be more helpful if we were supplied with more general taps’.*

*‘ Normally, the head of the household is responsible for fetching water, but in general, we drink unsafe water due to the high cost paid per jelly can. If you have made it to get money to pay, that water from the general tap is reserved for drinking, and the rest of the domestic activities are handled by using water from the other traditional water sources they mentioned’.*

*‘ Most of the time, if my children have gone to school and my wife and I are the only ones at home, I am the one who immediately goes to the well to that problem of water is solved as soon as possible. In general, I am the one who is in charge of fetching water; I can't let my wife go there when we need water as soon as possible because she is not fast as I am. But also, w hen the children get home from school, they may go there in my place if I'm busy doing something else, like feeding the cows’.*

*‘ I may not say that men are always the ones to go to get water because we are not always at home. Women or children are more likely to go there than men, who only go there on rare occasions. Coming back to the sources where we get water, we get it from swamps and ponds whose water has brought green-like plants known as "Imbobi" in our local language. Then, when you have money, you go to the general taps, which may also last more than three days without providing water. In that case, that water from swamps and ponds, is the one we use for cooking, and some people even drink it without boiling it, which leads to the other issues with intestinal worms. As my colleague number one stated, we are requesting that the cost of one jelly can be reduced from thirty Rwandan francs to twenty or fifteen Rwandan francs. Also, in the case of general taps that are only supplied with water on rare occasions, it would be nice if they were consistently supplied with water so that we could stop fetching unsafe water from swamps and ponds’.*

*‘ As a compliment to what my colleagues have said, safe water shortage is a very difficult issue because water plays a very big role on body, for drinking, and washing. So , when water is unavailable at the public tap or you are unable to pay for it, you may even spend two days without washing your body. As you can see, these are the consequences of water scarcity. Furthermore, due to water scarcity, people drink water from swamps and ponds, which is indeed unsafe for health and is frequently where they contract intestinal worms. Moreover, as you mentioned, it will be difficult for household members to wash their clothes and bodies whenever it is needed, which is a challenge to the body. As a request, it would be nice if water is supplied regularly to the public taps and if possible the cost per one jelly can should be reduced because there are other regions where it is paid twenty or fifteen Rwandan francs instead of thirty as it is here’..*

*‘ We are particularly affected by the effects of those public taps that are no longer functioning. For example, in our village, there are about three public taps that have only been operational for one week since their dedication, despite the fact that they were built five years ago. They have all the requirements, such as pipes, but they haven't been functional in over five years. Furthermore, while some taps are operational, the majority of them are built in centers near the road, so due to the long distance that they must travel to reach those taps, more than half of the residents fetch water from swamps because those are the ones closest to their reach. In that case, they usually come to the center to collect drinking water. As you can see, the effects are there because a child who is still young may not be able to distinguish between water collected from a swamp and water fetched from a tap, and as a result, he may drink unsafe water’.*

*‘ As we have been discussing the issue of safe water and its accessibility, let us put more emphasis on it because it is a major threat that we are constantly concerned about. So, this is Mutenderi sector, Mutenderi cell, and Tonero village, which is where we all came from; why do I have to mention all of this? Because I believe this conversation was not prepared in vain, and we wanted to let you know that the problem of safe water scarcity exists in that very village of Tonero. It is true that water is accessible in some places, but due to the high cost, many Tonero residents go to collect water from various wells. We believe that you will advocate for us so that we will have access to safe water because we may eat a balanced diet, but due to unsafe water, you find people's legs, cheeks, or eyes swelling’.*

*‘ Because of good governance, leadership is built from the smallest unit called "Isibo," and every single household must have one, mine is called "Icyerekezo." We were then taught to construct toilet facilities and handwashing stations known as Step and wash’.*

*‘ If we leave here right now, I am confident that we will be able to find out what I am telling you. At my house, we have a toilet with a cover, and when someone finishes using it, there is a step and wash with a piece of soap for proper hand washing. In general, most of the households in my Isibo have toilets and hand washing stations with soap; those who do not have these facilities are likely to be among those with poor understanding’.*

*‘ My house has a toilet that is covered, roofed, and has muddy walls, as well as a step and a handwashing station. The government has taught us a lot so that there is no problem of toilets and hand washing stations. Thank you!*

*‘ The toilet facilities that we use are the same as those that children over the age of five use, but those under the age of five use a small hole that we dig near the toilet so that after defecating, an adult person removes the feces and places it in the toilet’.*

*‘ Usually, a child below five years old remains at home with an older sibling. Therefore, once the child has used the hole, the older sibling takes care of it before it rains’.*

*‘ in our homes, we have one toilet that is used by the entire family and is properly constructed with a small hole so that some of the children can be trained on how to use it. However, for children under the age of five who are still young to be trained about using the toilet, they prepare a place where he will defecate, and his mother ensures that the waste is removed immediately after the use of that specific place, which is usually near the toilet that the family uses. Handwashing stations are present, and children over the age of five are taught to wash their hands after using the toilet; however, children under the age of five are washed by their mother after defecation or micturition’.*

*‘ In terms of water, hygiene, and sanitation, the availability of safe water is not an issue; however, the cost of it is. Another issue arises as a result of the people in charge of water distribution, who have set a specific time for people to come and collect water. As a result, when you are done with your activities and go there to fetch water, you may fail to get it not because there is no water, but because the responsible person has refused to open the tap, telling that you have arrived at an inappropriate time for water dispensing. Coming back to the cost per jelly can, the complaint is dependent on one's ability, as some may complain that thirty francs per jelly can is an issue, while others may say that even if it costs a hundred francs, they can afford it. In general, the main challenges we face in our community are the cost of one jelly can of water and the availability of the person responsible for water distribution. Some of those taps that provide water on occasion are also present; it would be advantageous if they were also supplied with water so that the surrounding community did not have to travel a long distance to get water from swamps. It would also be more beneficial if the responsible individuals were always present to provide water whenever community members needed it; in this case, there would be fewer challenges in terms of hygiene and sanitation’.*

*‘ The thing that should be done to promote the health of pregnant women and children is to include men in the teachings that promote the well-being of children and pregnant women because most of the time, women were only educated about the well-being of the family in general, but for those of us who are here today, we are fortunate that we now understand well the way of promoting the health of pregnant women and children. Life will prosper if all men are aware of this’.*

*‘ Gender-based violence against women, in my opinion, is the first hinderance to pregnant women's and children's health. If this violence is addressed, pregnant women and children's health will undoubtedly improve. The second point is that, as previously mentioned, we may be provided with safe water with fewer challenges, which would be more beneficial in promoting women's and children's health. Third, it would be more beneficial if we men were continuously educated about the balanced diet that must be fed to pregnant women and children because we are fortunate to be here, but our number is small in comparison to those who are not around, so it would be nice if education went beyond this number so that a large number of men are educated. There is no doubt that pregnant women and children will be healthy once all of this is done’.*

*‘ As my colleagues have stated, we are extremely fortunate to be here! Surely, my presence here has not been in vain; I have gained a lot and my understanding has improved to a certain level, and I will not keep this knowledge to myself because whoever I meet among my fellow men, I will make sure to share what I have learned that the responsibility for caring for the health of children is not only limited to women, but we as men must also take charge. Previously, I would have money and consume it all by myself, despite the fact that there was no salt at home, but today there is something that I have learned about promoting the health of pregnant women, even though mine is not pregnant, but it will be useful in the future not only for myself but also for my neighbors. Furthermore, men's education should be considered so that they can be gathered once in a while to be informed about the current situation. Thank you’.*

*‘ Support should also be provided to promote the health of pregnant women and children, because when a pregnant woman can get flour for porridge and milk, her life is complete in my opinion, but when that is not available, her health suffers’.*

*‘ There is a saying which states that "a house without a foundation is easily ruined", and the same holds true for a home with an irresponsible man, which is totally different from that of a responsible one. Men used to impregnate women and that was when they met, but now it is different because the man must take responsibility from the time of conception until the period of birth and later on. I believe that caring for a pregnant woman is one of the solutions that eliminates the risks to the pregnant mother's and fetus's health. Thank you!*

*‘ as the conversation progresses, new ideas emerge. To promote the health of pregnant mothers and children, I'd like to come back to the issue of conflicts. I'd like to return to us as men, a s the other man mentioned, a home with a man is the acceptable one, but we men have different ways of leading our homes, where you will find some families fighting over property rights, where one person is the only one with rights to the home properties while the other's rights are limited to the properties. In most cases, men have full property rights while women have limited rights. As a result, when a woman is pregnant and in need of something, it is out of her reach not because it is not available but because her husband has limited her property rights. The same is true for the child whose needs are not met solely because of the way his father treats the family's property. In that case, we men require extra education’.*

*‘ What should be done is for men to be educated on how to share family property with our married partners so that we have equal rights for them’.*

*Participant number 2: A pregnant mother should eat amaranth vegetables, eggs, and milk if she has access to it. She should also eat porridge and take enough time to rest’.*

*‘ A pregnant woman should rest as much as possible. A pregnant woman should also eat a well-balanced diet that includes vegetables, fruits, milk, and, if she does not have access to milk, she should take other animal-derived foods such as eggs. She should also avoid strenuous activities to avoid exhausting her body, which is also bad for the fetus's health if the mother engages in strenuous activities’.*

*‘ A pregnant mother should eat vegetables and take enough to rest. In order to avoid exhausting her body, the pregnant mother should only engage in activities that are within her capabilities. A pregnant mother should also avoid any kind of disturbance because whatever bothers her also bothers the fetus in her womb’.*

*‘ She needs to eat amaranth vegetables, eggs, porridge, and cow’s milk’.*

*‘ She should avoid eating maize and she should not take cassava more often’.*

*‘ She is allowed to consume any type of beverage except alcoholic drinks. The reason for this is that if a pregnant woman consumes alcohol more frequently during her pregnancy, she may give birth to a baby who is mentally abnormal’.*

*‘ A Pregnant mother should eat ripe bananas, avocado, and vegetables. She should avoid smoking and drinking alcohol. She should also avoid eating sweet potatoes or cassava because they can cause abdominal discomfort, preventing the baby in her womb from breathing properly’.*

*‘ A pregnant mother should avoid eating chilli peppers because it can cause her baby to be born with a congenital anomaly and its brain to be abnormal. A pregnant mother needs enough time to rest and conversation with her partner in order for the baby to be calm in its mother's womb and to be born happy’.*

*‘ A pregnant mother should not drink alcohol or smoke tobacco; instead, she should eat a well-balanced diet that includes vegetables, carrots, amaranth, and eggs’.*

*‘ Thank you, a pregnant mother should drink porridge and eat fruits such as tomarillos and passion fruits’.*

*‘ A man in a household should take responsibility of providing for his family a balanced diet. The beliefs of getting that balanced diet depends on the variety of foods that are available in our region. So, a pregnant woman is not allowed to drink alcoholic beverages; instead, she should consume porridge and other food varieties available in our community’.*

*‘According to my religious beliefs as a Roman Catholic church member, we see many women drinking alcohol, but a pregnant mother should not drink alcohol. As a man, I can drink alcohol, but it is not allowed for a pregnant woman’.*

*A woman is only allowed to drink alcohol (primus) after giving birth because it heals the wounds caused by childbirth. (laughs). She is not totally allowed to take alcohol during pregnancy’.*

*‘ A pregnant woman should eat soft foods and avoid smoking and drinking alcohol. Because drinking alcohol or smoking tobacco may harm the fetus in her womb, she should also avoid strenuous activities because her fetus's health is dependent on her own health. Therefore, what a pregnant woman eats is determined by what she is willing to eat as a result of her pregnancy. Before pregnancy, a woman may eat a certain type of food, but after getting pregnant, there may come pregnancy-induced refusal of that certain type of food; in that case, if that food is not among what you normally sow, you as his husband go to buy it so that she and the fetus stay healthy’.*

*‘ Yes, it exists, but it is dependent on what her pregnancy induces her to consume’.*

*‘ When a woman becomes pregnant, her body's functioning changes, so what she eats is determined by what her pregnancy induces her to eat. If she is unable to eat what the rest of the family is eating, you go to find food that meets her requirements’.*

*‘According to my beliefs, nothing is prohibited from consumption’.*

*‘ There are some religious beliefs that prohibits certain types of food. For example, some churches prohibit their members from eating small dried fish or fish in general, despite the fact that we know that small fishes are not only beneficial for a pregnant mother, but also for a woman who has already given birth, because the baby gets nutrients from his/her mother's breastmilk. Therefore, once a pregnant mother belongs to that kind of church which prohibits its members from consuming certain kind of foods, it does not only harm her body, but also the fetus in her womb. We all know the examples of women who suffered from malnutrition not because of a lack of food, but because they followed their churches' doctrines. However, I would advise the churches' priests to allow their members to take whatever is beneficial to their health during pregnancy because it is a pregnant woman's responsibility to consume a well-balanced diet that includes body-building foods, protective foods, and energy-giving foods’.*

*‘ For example, I am not a member of the Seventh-day Adventist Church, but I have heard that their members are not allowed to consume small dried fish or animals without a divided hoof’.*

*‘ The members of Islam and Seventh Adventist Church do not eat pig’s meats and there are other denominations which do not accept the use of milk in their members. But on my side, I don’t agree with those churches’ doctrines because my conscience tells me that I should provide whatever is necessary for my wife whenever she is willing to consume it during pregnancy’.*

*‘ A pregnant woman is not allowed to consume chilli peppers or hot foods because they are harmful to the baby. When the food is ready, the mother should wait for it to cool down gradually and take it when it is not too hot because that is when it will be more beneficial to the baby and her own health. Actually, the baby's nutrition comes from his or her mother's breastmilk, and his or her mother's breastmilk comes from his or her mother's nutrition status and normal blood circulation. Therefore, a pregnant mother should eat eggs, porridge, and vegetables like amaranth, as well as take enough time to rest. That’s what is necessary for the pregnant mother’s health’.*

*‘ In general, the child should be breastfed for at least two years. That is the age at which the child has absorbed the necessary nutrients from his/her mother's breastmilk and cannot be easily malnourished. So, immediately after birth, the baby should be put on his/her mother's breast so that he or she can consume his/her mother's colostrum, which is important. Thank you’.*

*‘ When a baby is born, he/she is given complementary feeding for the first six months of his/her life. Immediately after birth, the baby should be given breast milk, and within six months, depending on the parents' financial ability, the baby should be given a complementary feeding, but within that period, they may also give him/her a well-filtered porridge’*

*‘ Yes, the baby is given the complementary feeding during the first six months of life’.*

*‘Normally, during the first six months of life, an infant should be breastfed exclusively. For financially stable families, after six months, they begin giving their children fruits, and at one-year-old, the child begins to eat what the rest of the family eats. Therefore, after six months, a child is given complementary feeding that includes fruits and porridge, and as he or she grows older, they begin to train him or her to eat what other adults eat’.*

*‘ For a baby to grow normally, a baby from birth should be given a complementary feeding up to six months. Then after six months, an infant is given a well-balanced diet which includes milk and fruits such as ripe bananas’.*

*‘ The program of one cow per Rwandan household was implemented in this region. Some people have cows, while others do not, but those who do not have cows make sure to buy milk for their children on a daily basis as a complementary feeding’.*

*‘ A baby is given his/her mother’s colostrum immediately after birth. At four months of age, he/she is given a mixture of milk and biscuit to make him/her thrives better. Then, at six months, he/she is given a pound of tree tomatoes and ripe bananas’.*

*‘ Yes, I did it, and that's how I do it to help my baby thrive because his mother can't exclusively breastfeed him until he's six months old’.*

*‘ An infant is exclusively breastfed in my home from birth to six months of age. Even if it is a well-filtered juices from industries such as passion fruit juice or tree tomato juice, it should not be given to an infant under the age of six months. Coagulated milk is also avoided to be given to an infant who has even started taking complementary feeding. When an infant is older than six months, he is given fresh milk’.*

*‘ An infant is exclusively breastfed from birth to six months. For example, even though my child wanted to eat before six months, we breastfed him for six months without giving him anything else. We started him on fruits at six months and then moved on to soft foods. It is not totally acceptable to give complementary feeding to an infant before six months! Thank you’.*

*‘ The complementary feeds that should be given to the children are cow’s milk and goat’s milk. Alcoholic drinks are also prohibited to be given to children. There are also foods that are prohibited during the child's introduction to complementary feeding because solid foods such as cassava cannot be given to an eight-month-old child. The child is then given food appropriate for his or her age; for example, at eight months old, the child should be given soft (semi-solid) food such as a mixture of pounded green vegetables and small fishes’.*

*‘ At six months, a child is given a complementary feeding that includes small fish and a porridge made from sorghum, maize, and soya bean flour’.*

*‘ The child should also be given fruits such as ripe bananas, tree tomatoes, and passion fruits. Thank you’.*

*‘ Cassavas and sweet potatoes should not be given to a child because he can’t be able to chew it’.*

*‘ Regarding the point that the child cannot be fed a certain food plant, yet it is what you had, this is not true; rather, what matters is how you prepared it. You can have that sweet potato, peel it, mix it with beans and amaranth vegetables, and when that child's teeth haven't yet erupted, you have to make a pound mixture of all that food so it doesn't get stuck in the child's esophagus. When you prepare that sweet potato in a separate saucepan for the child, then mix it with carrots, peeled tomatoes, and soya bean flour, a child who has been introduced to complementary feeding can eat that mixture without any problem. The complementary feed that should not be given to the children before one year and half or two years old is maize because sometimes it is even difficult for adults to chew’.*

*‘ The reason for not giving maize to a child, is that the coat of the maize seed is easily digestible by the stomach, which is used for digesting other solids food. Therefore, you should not give maize to a child under the age of two because his or her digestive system is not yet strong enough to digest the coat of the maize seed’.*

*‘ Yes, we have a separate saucepan for a child because I take a small saucepan that corresponds to the child’s age. Then I make a pounded mixture of one or two sweet potatoes, amaranth vegetables, small fishes, and soya bean flour to keep the sauce from becoming too loose. When that mixture has lost its temperature, you serve it to that child, and if you are fortunate enough to have porridge, it is even more beneficial. It is not necessary to give that food to the child by force, if he/she is not willing to eat it, his/her mother can eat it and the child will eventually get the nutrients from his/her mother’s breastmilk’.*

*‘ A six-year-old child is allowed to eat sweet potatoes, yams, cassavas, vegetable, carrots and egg plants. But that mixture should be pounded so that it will be easier for the child to swallow. Then, for a four-year-old child, he is given milk and biscuit only, he/she doesn’t eat food. Thank you’.*

*‘ A child in his/her first year should eat everything but there should be vegetables because they are good for vision. Thank you’.*

*‘ A child during his/her first year, is started on drinks like milk and porridge that is well-filtered so that it will be easier for his/her digestive system to digest because his/her digestive system at that age is not developed enough to deal with solid foods. For the case of food, you give him/her ripe bananas and a fresh juice of tree tomatoes without seeds because his/her digestive system can’t digest them. When the child is one year, one year and a half, or two years old, you are allowed to give him/her whole ripe bananas that are not smashed and tree tomatoes until he/she is also allowed to be given different types of soft biscuits. There are hard biscuits, such as Mary brand, that should not be given to a child under the age of one. That Mary-branded biscuit is given to a child when he or she is one and a half or two years old’.*

*‘ There is a type of biscuit that contains milk; I don't recall the brand name, but it is soft enough for a child’.*

*‘ My child has not yet reached the age for complementary feeding, but I fed his older siblings in that way’.*

*‘ Regarding the type of biscuit that a child should eat, for my side, I give that Mary-branded biscuit to my children. Mary-branded biscuit is the softest biscuit of all types of biscuits that are given to children instead of sugared biscuits because it is not good to give sugared products to a child who is still young. It is not even advised to put sugar in a child's porridge made from a variety of nutritious cereals. He/she should drink it in that way, and possibly be given sugar at a lower level, at least when he/she is at age of one year’.*

*‘ A child should eat four times per day during the first year of life. In the morning, at eleven o'clock, noon, and evening’.*

*‘ Even though I am not always at home, that is how it is done because I discussed it with my wife, who is always caring for those children’.*

*‘ A one-year-old child eats three times per day. He drinks porridge and eats the leftovers from supper in the morning, and he also eats at noon and in the evening’.*

*‘ Normally, the frequency with which the child should eat depends on his appetite because whenever he says he wants to eat, you should immediately give him food; however, even if he does not say so, you must ensure that there is food reserved for him so that it is available in case he asks for it. As I previously stated, when a child is still young, he should have his own saucepan where his food is prepared. In case when you give him food that was prepared at noon and he refuses to eat it, if you had prepared for example sweet potatoes, at least at evening you should change for example find Irish potatoes but don't forget to include vegetables and soya bean flour because we adults also don't like eating unchanging foods. So, if we adults change our meals, it is more recommended that we do so for a child's food, whose health is more critical than ours. If he was given porridge at noon, and you should give him fresh milk in the evening’.*

*‘ Normally, you can’t' tell how frequently a child should be fed because the child eats whenever food is available, but the child should not eat the same foods as adults instead a child should have his own separate saucepan as participant number four said. If adults have planned to eat cassava as their meal, the child will not be able to eat that cassava rather they have to prepare for him bananas with soya beans flour, and small fishes. Otherwise, the number of times a child should eat in a day can only be determined by how you trained him. If it is once a day, it is once a day; if it is five times a day, it is five times a day. For example, I can't tell how frequently I should eat because I eat whenever food is available, but a child should be fed at a specific hour that differs from that of adults’.*

*‘ Depending on my financial ability, at my home children eat at least four times in a day. However, I feel like whenever the food is available, my children should eat because they are the ones I always strive for’.*

*‘ Culturally, I think there is no food that is prohibited to be given to a child in his first year instead what matters is the preparation’.*

*Participant number 8: If it is Irish potatoes you planned to give him, you have to take it and mix with a small fish flour and smash it to make it easier for him to swallow. Thank you’.*

*‘ During the child’s first year, he is not allowed to be given coagulated milk and alcoholic drinks’.*

*‘ For my side, I don’t know that kind of food that prohibited socially or culturally’.*

*‘: Alcohol, tobacco, and other tobacco-derived products, such as "Ubugoro," as it is known in our local language, are not allowed to be given to children. That is how I believe; otherwise, a child should eat any other type of food. You must properly prepare it and avoid including those dangerous products until he is old enough to choose what kind of food he is willing to eat’.*

*‘ Thank you; in our community, there is no culture that disallows certain foods, but there is one that forbids giving alcohol to children. In our community, no child is allowed to drink alcohol unless they are disobedient to their parents and have left home; otherwise, a child should be given everything but the in charge of preparations should be mindful of hygiene during food preparation in order to fight diarrheal diseases. In summary, what I think that is not allowed in children is alcohol consumption and tobacco smoking in their presence because tobacco is not only harmful to adults but also to children’.*

*‘ A child at one-year-old is not allowed to be given maize because his stomach is not developed enough as that of adults. Cassavas as well are not allowed to be given to that child because cassava is one of the tubers that are hard. The child is then fed soft food such as bananas or Irish potatoes until he reaches the time when his stomach is developed enough to deal with hard foods like that of adults do’.*

*‘ Regarding meats, a child can eat meats; rather, the issue may be eating a lot of it because the child may be used to meats and when you feed him other foods other than meats, he refuses and claims that he wants meats but you cannot find it at that time’.*

*‘ Except when members of the denominations imply that they cannot eat this type of food. Otherwise, I and my children share the available food because I do not belong to those churches with such understanding. Thank you’.*

*‘ Yams are not allowed to be given to a child who has not yet started walking. Sweets should not be given to children as well because they pre-dispose the child’s teeth on decaying’.*

*‘ When he eats them during the period of crawling, it causes him to delay walking’.*

*‘ Normally, children in this region we eat every type of food that is available because that's when children become stunted when parents start selecting certain types of food for their children. When that yam or sweet potato was well prepared and pounded, can be given to a child without any problem. Otherwise, what is prohibited to be given to a child is alcoholic drinks’.*

*‘ Thank you; there comes a time when a pregnant woman becomes weaker as the pregnancy progresses; in that case, I take charge of preparing food, and when it is ready, I serve the pregnant woman first, followed by the children. Therefore, when there is a young child, he is the one who is served first, where he is given his food on his own plate, and then his mother takes care of him because he is unable to feed himself like his older siblings’.*

*‘ Normally, the serving of food in a household is determined by the customs of the household. If a child under the age of one is present, his food is prepared in a separate saucepan and served on a separate plate. If there are other children over the age of five, you as a parent know each child's appetite and serve them food on the same plate, and if they are not satisfied, you serve them another food. However, my wife and I share food on the same plate, not because there aren't enough plates, but because of household customs. So, for example, when you begin eating food when children are not present, we reserve everyone's plate with the same quantity of food per each’.*

*‘ A pounded mixture of banana, tomatoes, and small fishes is what I consider to be quality food for children. That food is of high quality, and it is commonly available in this area’.*

*‘ To maintain the quality of the food during preparation, if they are those tomatoes that you want to add to the banana, you must first wash them and make sure that the small fishes that will be added are grinded into a flour. That food, in my opinion, is of high quality’.*

*‘ I have a five-year-old child and a three-and-a-half-month-old child at home. That three-and-a-half-month old child has not yet started eating, but that five-year-old child eats whatever I eat. To maintain the quality of the food we eat, we make sure that the plate we use is clean and well-dried. Cassava flour, sweet potatoes, yams, and cassavas are among the foods available in this region’.*

*‘ Though most people are not so used to it, the quality of food is usually determined by cooking it when it is fresh and without the addition of oil or other ingredients. Perhaps ingredients will be needed another time because they are also beneficial in some cases, but quality food is non-fried food. Therefore, if you take green bananas and mix them with beans and vegetables to make a pounded mixture, even if no cooking oil is used, I consider that food to be of high quality’.*

*‘To ensure food quality, you must first clean the utensils that will be used in food preparation. You must first wash the saucepan, then the food, and then cook it in the previously washed saucepan. When the food is ready, allow it to cool before serving it to the child; this is when the child is served quality food’.*

*‘ Normally, I do not think that quality food is food that we expect to be delicious; rather, I am certain that the people in charge of food preparation are the ones who ensure the quality of the available food varieties in their preparations. Therefore, every available food can be of high quality depending on how it is prepared, and the mother of the child should ensure that the child is served high-quality food’.*

*‘ Thank you, first of all, keep in mind that not all of us can afford to buy chicken, but some of us can afford to buy a half or one kilogram of meat from the butchery. To tell you the truth, there is a way in which children are cared for, but obviously the children will not be given the meats and their father, who bought those meats, will be ignored; instead, those children will be served but also their father be cared for in another way. Otherwise, the size of the meats may be the same, but everyone is served accordingly so that everyone feels included for that activity’.*

*‘ Normally, I don't go into the kitchen, so whatever my wife serves me is what I get. Since the wife is the one who prepares the food, there are times when she can even eat some in the kitchen before coming to serve us all, so whatever she brings after preparation is what we eat. Therefore, I can't really know the person who gets a large portion of meat’.*

*‘ In terms of buying meat, there are those who buy cow's meat, others who buy chicken, and even more who buy pig's meat, but I buy cow's meat. Because I have two children, one kilogram is sufficient for me and my family. But most of the time when I buy meats, they get ready when I am sometimes not around, but I am aware of one of my two children who likes meats a lot, and he is the one who gets a lot of meats in my opinion’.*

*‘ Let us not brush over the truth about meats; normally, the woman and children get meats simply because I bought it for them. So, when the children's mother is cooking those meats, she praises God and remembers the sacrifice I made to bring those meats. Therefore, a big part of meat is then given me who has bought it. Thank you.’*

*‘ Except the selfishness of men, a man cannot normally spend a lot of time somewhere outside of his home and eat nothing; it does not exist; he may not have bought that meat for the rest of the family, but he ate it outside where he spent his day. What I, myself do first of all, there are times when I come back home when the children have already slept; in that case, I cannot say that my wife does not have to feed the children before I come back home; they are the ones who are served food first, including that meat, because there may even have been a time when I ate that meat at the bar. Therefore, I can't disallow my wife and children to eat before I come back home as a matter of making them to keep for me a lot of meat. That's how the situation is at home.’*

*‘ A wife is generally the heart of the household. Because he does not spend his day at home or go into the kitchen to see what his wife has cooked, a husband should be satisfied with what his wife has served him. A man is generally satisfied with what they have served him because his only responsibility in the case of that meat is to provide it for the family, and the rest of the duties are left to the wife. A man's only responsibility is to ensure that his family is happy with what he has provided’.*

*‘ Normally, every parent's pride is their child; therefore, if I am fortunate to find meat, I am pleased to see my wife's and children's satisfaction. Therefore, I am not the one who gets the big part of the meat; rather, the big part of the meat is eaten by the children or their mother. I think that is what I had to share’.*

*‘ my wife does not normally eat meat. If we buy meat, my child and I are the only ones who prepare it and eat all of it.’*

*‘ A big part of the meat is given to a man because he is the one who buys it for the family. For example, in case of chicken, a woman cannot eat a chicken leg because the chicken leg belongs to a man (laughing). Thank you’.*

*‘ Yes of course, the chicken leg is mine (laughing)’.*

*‘ It is undoubtedly known (laughing), it is known that the chicken leg belongs to a man’.*

*‘ No, however, a wife who obeys her husband should be aware that the chicken leg should be served to the man. It is even the same case for chicken gizzard (laughing)’.*

*‘ Normally, I am the one who has said that a man eats a big part of the meat, but that does not imply that I am the one who prepares it. When I buy meat, I give it to my wife to cook it; when they are ready to be eaten, I give each one his or her part’*

*‘ I don't eat a lot of them so that the rest of the family will not be satisfied, but I do choose the largest one because I'm the one who bought it for the family. That is the reason why I said that the man is the one who gets a big part of the meat’.*

*‘ To tell you the truth, I am pleased by my wife's appreciation. So the wife appreciates it when what I provide for the family is satisfactory. I couldn't be pleased by buying that meat, but my family was dissatisfied simply because they had to give me a large amount of it. On my side, if my wife and children are satisfied with what I bought for them, I am happy for that regardless of the quantity they gave me’.*

*‘ first of all when you realize that the child is ill, you take him to the community health worker where he/she goes on with the examinations. When it is the problem that can be managed by the community health worker, he/she gives you medications and tells you how the child will be taking them. When that child is found to be sick then, it is obvious that his feeding also changes, in that situation, the porridge should be available to help him to take his medications. That is the pattern I follow in my household when a child becomes ill. Instead of going to the pharmacy to buy for example paracetamol just because the child has a headache, I first go to the community health worker, and if he/she is unable to manage the condition, he/she immediately transfers us to the health center’.*

*‘ Normally, the parents should watch over the child’s state, if the child becomes ill, his mother washes him and takes him to the community health worker. If the community health worker is not able to manage the condition he/she transfers that child to the health center. I think that’s how we care about our children’.*

*‘ When a child is sick, he is not given hard food. Instead, porridge should be available, and if you can find him an egg, that will be even better because you cannot give a sick child sweet potatoes’.*

*‘ A child suffering from diarrhea is not given sweet potatoes; instead, the child is taken to the community health worker for treatment. But when the child falls sick during the night when you can’t find the way of taking him to the community health worker, you find the medicinal herbs to him in the mean time you are waiting for the morning so that you take him to the community health worker. Therefore, regarding food given to a sick child, I can’t give sweet potatoes for a child suffering from diarrhea instead I find for him rice and wheat flour products because they also help to stop that diarrhea. I have to also find porridge for him’.*

*‘ To give you an example, even though I do suffer from malaria, I have never taken malaria medication. When I have malaria, I take lemon leaves, pawpaw leaves, and five passion fruit leaves and mix them in water until it turns green, then drink it. When it gets to the evening and I'm still feeling ill, I mix another herb with the other leaves mentioned before and boil them together and I drink that water when it's half hot.So, I and my older child never go to the hospital but the young baby, the other one who is three and a half months old is taken to the hospital whenever he becomes sick’.*

*‘ I take the other herbs I mentioned earlier, lemon leaves, three passion leaves, and pawpaw leaf, and mix them in water for the child. When I leave home in the morning and have given him that medicine, when I return and see him outside, I realize that he is getting better and give him another dose until he is completely recovered. When a child is treated in that way, he does not always become ill, but when he is taken to the health center, his condition may worsen, and you find that that child's health become poor because he easily fall sick’.*

*‘ Yes. For example, when a child has fever and chills, that is malaria but when the child is passing watery stool and he is going to the toilet more often, I realize that he is suffering from diarrhea. That’s how I know it’.*

*‘ You have to take a sick child to the health center where they help him so that his life is saved.’*

*‘ You can’t find the treatment to give to a sick child except when you are a community health worker’.*

*‘ Normally, the healthcare system is decentralized, when a child becomes ill, there is no need to seek out herbs; instead, you take him to the community health worker, who transfers you to the health center if he is unable to treat the child's condition. We don’t have to give herbs to children because they are not cows or other animals which eat grasses (laughing)’.*

*‘ Normally, there is an age group of children where it is difficult to tell if they are sick, such as newborns because they are not able to tell you where they feel pain. Most of the times, their mothers are usually the ones who predict whether they are sick or not. If she is breastfeeding the baby, she will feel whether the child's temperature is high or not, and she will also observe how the baby takes the breast to see if it is the same as usual. However, there are other children who can tell you exactly where they are experiencing pain if you ask them. So, if a child of any age becomes ill, instead of wasting time looking for medicinal herbs where you may not even be knowing what specific condition you are going to treat, you must take the child to a community health worker because the longer you wait, the greater the risk of seriousness in the child's condition, which may even lead to death. We are grateful to the government for decentralizing the health-care system and locating it near us. In our villages, there are community health workers who have been trained and are equipped with all of the materials and test kits, so that whenever you come to them with a fever, they do the exams and, if someone's condition is beyond their ability, they immediately send you to the health center. Therefore, I never take a lot of time in looking for herbs or trying to do some home treatments for my children if they become ill because if I do so, I may even be dealing with something that my children do not have. Instead, whenever a child becomes ill, I immediately take him to the community health worker, who may refer me to the health center if he is unable to treat the child's condition, and if the health center is unable to help, they may refer me to the hospital’.*

*‘ my household has access to safe drinking water because we get it from a borehole, and I have heard people saying that borehole water is of higher quality than water distributed by the Energy, Water, and Sanitation Authority (EWASA). I even see some of the people who have these EWASA taps coming to the borehole sources. But, those who want to be certain about the water they drink, on the other hand, boil it first, and others use other filtering mechanisms. One of the mechanisms utilized is the use of cotton and gravels; when dirty water is put in a container containing cotton and gravels, it is filtered; however, if complete cleanliness is desired, the water is boiled. That is the method I am aware of that people use to have clean water. Yes, it is at the borehole’.*

*‘ When I am at home, I even take like to jelly can and go to bring water so that my children may rest a bit but when I am not at home, the wife and children are the ones who go to fetch water’.*

*‘ Water's safety or cleanliness does not depend on where it was collected; rather, water's safety is ensured when it is boiled first, then filtered and put in a well-cleaned container. When you drink that water, you have drunk safe water. Thank you’.*

*‘ As we all gathered here, we all get our water from the same source. There was a project called "Gira Impuhwe" that built those boreholes, and there came other sponsors who took that water in the laboratory for testing and confirmed that it was safe. Therefore, we are confident that our water is safe, but the containers must be thoroughly cleaned. That’s how I understand the safety of our water’.*

*‘ To ensure the safety of water, we clean the containers and then go to fetch water for drinking and other domestic use’.*

*‘I have a toilet facility. It is built with mud bricks and there is a sac on its entrance as a door’.*

*‘ No, a child under five years uses a place nearby the toilet and when he ends up passing stool, his mother takes it in the toilet’.*

*‘No, the place that the child uses a small pit not a flat surface’.*

*‘Our toilet is adequate; it is closed and the diameter of its opening is not greater than twenty centimeters, allowing a child of about five years to use it; however, those young children who are unable to use it are assisted by their mother, as she is the one who is always with them.’*

*‘ I think that is known by women because I never mind about those children are helped in case they need to use toilet’.*

*‘ That toilet used by adults should not be used by babies because there is a risk that they will pass through its opening; instead, a child under the age of five should have a potty or defecates somewhere else so that his mother can remove the feces and put it into the toilet’.*

*‘ In my household, young children who are unable to use toilet, have potty’.*

*‘ the toilets that we have, are those toilets that can be easily used by adults and each toilet’s nature depends on someone ability. Depending on my ability, the toilet that I have is not worth but I have also tried my best to find the way of helping my child who is under five years old though I didn’t know the name of that material but I have heard from participant number eight calling it a potty. Therefore, I have that potty for my child’.*

*‘ The water that we use is got from the boreholes and the soaps are accessible depending on everyone’s financial ability because you can a piece of soap per one hundred Rwanda francs. Therefore, the hygiene is like a habit because you can’t wear a cloth which is not washed or bathe only water, we use both water and soaps’.*

*‘ Yes, there are step and wash which has water and soaps, whenever a person ends using toilet, he/she washes his/her hands’.*

*‘ Yes, but it is not fine, maybe someone can give us another one.*

*‘ Yes, I have a step and wash that someone steps on and wash his or her hands but for the soap, I go to take where we keep in the house because when you leave it outside on the step and wash, they can steal it’.*

*‘ We don’t have any problem with water because we have those taps from EWASA and the others boreholes that my colleagues were talking about’.*

*‘ Water is available but the access to soaps is expensive. There is even a time when you can wash utensils without soap just because you are not able to afford it’.*

*‘ you cannot be able to put all things in the right place! There is a time we clean that water and put it in a container as a water for drinking but there may also come a time when that water is not there and drink what is available whether it is boiled or not. Therefore, in terms of hygiene and sanitation, I can’t say that it is enough because it is not done properly’.*

*‘ I am not sure of what should be done because today I may be at home and do properly all of what should be done or I may be not around and the wife does it but it is also possible that I and my wife may be not around and when we come back, we use what we will find there’.*

*‘ Thank you, normally, it is possible that men do whatever it takes to promote the health of their pregnant women and children but it doesn’t reach to the desired level. If there was another kind of support, it would be of more help for the promotion of pregnant women and children’s health. For example, I can go to work for two thousand Rwandan francs, yet, that baby needs fruits and his older siblings need food, to be honest that money won’t be enough to buy all of that. But there may be a time when God may make a way and there comes a support so that all of those fruits and food are provided’.*

*‘ When we men have money and have what to do, we can improve the health of pregnant women and children. But nowadays, it will be even worse because of drought; there will be no beans, vegetables, rice, or sugar. What we had to do was cultivate, but we don't have enough fields, which is also a challenge. I think there is nothing we can do to improve the health of pregnant women and children’.*

*‘ For my side, there is great need of support because the money we used to work for is no longer enough to afford the family’s needs. We used to work for two thousand Rwandan francs and it was enough but nowadays, that money only buys one kilogram of rice and soap and that is it. What needs to be done is that they can give milk and porridge flour to our children there at schools’.*

*‘ Thank you. In general, all of us are not of the same ability of satisfying pregnant women and children but a pregnant woman is allowed to work so that she promotes her health and that of her child’.*

*‘ Normally, the husband is the one who takes the household's decisions. You must not be that man who is always drunk and unconcerned about the affairs of the household. If the woman is pregnant, you must come back home early to assist her in caring for the children because the woman's body weakens as the pregnancy progresses. There is also another example of a man who has hens and forbids his wife and children from eating eggs because he wants to sell them, despite the fact that they need to eat them to benefit from their nutrients. I think that we, as men, must turn on ourselves and be involved in each of the affairs of our households’.*

*‘ On my side, I am always pleased providing something beneficial to my wife and children and if it is that egg, they can eat it. Usually, there are times when you cannot tell if a hen has laid eggs because you were not there during the day because most of the times the things of the household are known by the wives who are always there. From my point of view, there should not be conflicts as a result of something that has been eaten by the child or wife.’*

*‘ Normally, when there are conflicts in a household, even children are disturbed and do not do their duties effectively including their schools' attendance; however, there are no such conflicts in this community because most of us have been trained about the relationship of the household's members, and prayer is one of the weapons that protects the household from conflicts’.*

*‘ A pregnant woman is recommended to take green vegetables, meat, rice, fruits mostly those that increase blood in the body so that she can keep being better.*

*‘ A pregnant woman is restricted from eating sweet potatoes, corns and taking alcohols’.*

*‘ the reason why a pregnant woman is not recommended to take corns, smoke alcohol and those potatoes, there are no enough nutrients in them’.*

*‘ For me I think that since the woman is pregnant, she is like a sick person so she is supposed to take those corns because they also contain some nutrients but at the controlled level for if they become too much, they will affect her body’.*

*‘ Too much corns, not only to a pregnant woman, even in normal life there is where one gets stomachache in case he or she consumes a lot due to their outer courts(taste) and may cause different problems. Therefore, this shows that even it can be worse to a pregnant woman’.*

*‘ I am number eight, I would like to complement on number seven’s idea. So I think you can see that if a pregnant woman consumes a lot of corns, they will cause alcers to her, therefore, it it can be important if she consumes less’.*

*‘ Since there are health advisors in villages, they go on training people where they show them how they can maintain vegetable farming and show those with poor diet how they can do it’.*

*‘ We would like you to recoup on the question because it is holding a powerful idea’.*

*‘ So according o my opinion or generally, what I can say is that for example there are some people that are not allowed to eat meat, that may be due do different beliefs but for me I emphasise that everything that has an advantage to a pregannt woman and her baby should be taken regardless to beliefs because for example when someone is sick, there are no medicines that will not be prescribed due to his or her beliefs, she is supposed to be treated accordingly’.*

*‘ according to me, to those restricted things to apregnant woman like alcohol or cigarette, I am very sure that I can not alow my wife to take them in case she is pregnant. To the bliefs therefore, we all know that eggs, fruits and vegetables could be better to apregnat woman since they contain all nutrients needed by the pregnant woman and the baby in the womb’.*

*‘ According to my beliefs, I think that in case my wife is pregnant, she has to take green vegetables since they have no harm to her and prevent consuming a lot of corns, alcohol and smoke pregnant in order to make her life better and safe.’*

*‘ I hgot confused on the thing of consuming a lot of corns, we all know that our ancestors used to stock corns and beans as the main food for their daily life and they peacefully. Now may you please explain for us how those corns affect women?*

*‘ so what I can say on the question raised without compromising others' ideas is that even corns are among the carbohydrates, which means they can be also important to apregnant woman. We should consider that when everything is consumed in alarge amounts, it becomes worse. For me I think that she is supposed to get every thing becausese sometimes she may get addicted to these corns. Everything should be consumed in a controlled manner.’*

*‘ usually we are being trained and councelled, there are advices given to maternal mothers either in the village meetings where the health concellors help these mothers from the time of birth to atlest two years with the effective brest feeding. Therefore, when a baby is quitted from breast feeding before two years, you are mistreating that baby.This doesnt mean that in the whole of this time of breastfeeding, the mother should stop taking balanced diet.’*

*‘ it sometimes happens when ababy is breastfeeding and before getting full, the mother removes the baby from the breast. In the few knowledge we were given, they told us that the first breast milk doesn’t contain enough nutrients as the one coming after sometime. So this can retard the growth of the baby’.*

*‘ A maternal mother should breast feed the baby in first thirty minutes after birth so thet the baby can get used to it and increase relationship with the mother,then from the the time of birth to six months the baby has to be breastfed without any other thing included in the diet because the baby’s stomach is not yet able to digest the food.from the sixth month to two years the baby is given other few complements to the breastmilk like poriedge, with the maintainance of hand hygiene.that is all I can say’.*

*‘ a baby has to be breastfed at anytime in need of it. I meant that before the two years the baby can be breast fed at anytime in need of it’.*

*‘ they say that at the age if six months is when abay can start being given complements and these include fruits, yellow banans, vocado, passion fruits. There are also other sources of nutrients that the government has prepared for maternal women like flour for porridge’*

*‘ at the time that a baby should quit breast, I don’t know what they can do when it happens that a woman gets pregnant just after ababy of one-year-old, so by then I don’t know which time is predicted for that young kid’.*

*‘ at that thing, after the six months that a baby breast feed, considering my kids that ate at five months because they used to eat infront of them and cause them greed, and you find that sometimes after breast feeding, they could cry for more food because of others.’*

*‘ when a baby is six months, it’s the time to complement for her or him to increase strength because by then breakmilk is no longer enough for him or her.so that is why they find some complements to complement to the breast milk like porridge, passion fruits and yellow bananas’.*

*‘ Corns as it has been said earlier are restricted to be given to a baby as a ccomplementf or the breast because his or her stomach is not able to digest them. yeah, they contain nutrients but they are too hard for the baby’s stomach to digest that is why she or he should be given porridge flour” shisha kibondo”, green vegetables ans sweet potatoes are also restricted to the baby becaause even though they are important, they can be hard for the baby to swallow. The baby needs soft things that can be easily swallowed and they should be given while measured’.*

*‘ starting from the question you asked before, my idea is that there is no predicted time for the baby to be started being given breast complements exept at sixth month because the research shows that at the sixth month, breast milk is no longer enough for the baby. So the complement is usually made of balanced diet that is mixed with different things and make them easy for the baby to consume.the porridge given to the baby should be of mixed flour. They also say that ababy below one year of age is not recommended to take milk because the stomach is not yet able to digest that milk’.*

*‘ usually in the first yea, this is when the baby is getting used to food which means she or he need that balanced diet, which includes carbohydrates, proteins and vitamins. There we will come back at the green vegetables that helps in prevention of diseases and keep in mind that even those carbohydrates should be given in a controlled way.the baby is supposed to get the balanced diet in sufficient way’.*

*‘ according to my beliefs, that diet given to achild in the first year should include mostly porridge with mixed flour, then to the carbohydrates, since they are too hard for the baby to chew and swallow, they can churn them and give them to the baby easily and also fruits. that was my opinion’.*

*‘ then coming back to what you asked that how often can the baby be given food, usually anytime the baby needs food, she or he should have it, God forbid for the baby not to get food in case of its need’.*

*‘ complementing on what my college said, any time the baby needs to breast feed should have it, but a one-year baby or below should not be given food anyhow because the stomach is not yet used to digestion as an older person. We even kow that even older people cannot sustain the consumption of much food or eating every timetherefore, a one-year kid should be given food thrice a day after brest feeding for few minutes. The hygiene should be maintained to prevent some diseases’.*

*‘ and also breast feeding should have time, one should determine the time to feed the baby be because you are the one in charge since the baby is un conscious of what is allowed or not. So for me I think a mother should feed her baby atleast four times a day’.*

*‘ we can say corns, sorghum that is not flour, all those are not allowed to be given to a one-year baby’.*

*‘ the reason is that the baby at that age is too young to chew, swallow and digest them’.*

*‘ may be complementing on what you said, I think this is realted to our culture, not only culture, even as mature people, you can tell, usually it is a taboo to give a young kid alcohol by then, you will be killing that kid. But it sometimes happens where some kids are raised by alcohol which is a problem’.*

*‘ for me I will need your support because maybe I am not experienced in this. Since I just have onlybone kid, yiu can please share with me your experience and I can be yiur good ambassador’.*

*‘ I am a man with first brns as twins, I will say about my own experience because you can’t know someone’s experience, hhh. In the morng my wife wakes up warms the food and give it to the kidsthen after thirty or fourty minutes, she gives them porridge and I also have some so that my kids can have appetite, at lunch and supper we get food, but for the kids it is special because they normally say that the stomach of the babies under two years are not like those of adults, there is their diet. All of that is accompanied by hygiene and we always emphasize o the balanced diet. we were blessed to have to afford anomal products like milk, eggs, and silver fish. I give them milk because they are already two years’.*

*‘ we usually know that pregnant mothers and their babies have a very strog relationship. Pregnant mother is said to get hungry so much, it is clear since they are now two, even the one in the womb. So before giving me food they have to first feed my kids and even the pregnant woman has to be taken care of as they keep givimg her the balanced diet’.*

*‘ the available diet for the kids are the vegetables’.*

*‘ is possible that we are all at a different level to afford the whole diet. They usually say that ababy should eat and be satisfied but consume food containing balanced diet, but if you just give only potatoes so that she or he can be satisfied, by then you are just giving that baby only one thing out of many you should give. Maybe if I llok at our district, the mostly grown food is potatoes, but not only all of the people have them, but they can be easily afforded. So, we can get those green vegetables and potatoes, find some eggs and sometimes milk and small fish then try to give them to our kid so that they can get a balanced diet’.*

*‘ The major source of balanced diet is usually vegetables, for those who are able can afford fruits in markets. To the issue of porridge, the government has made it easy for us’.*

*‘ It is beans, potatoes and green vegetables only. Thank you’.*

*‘ I kept giving examples on my family. the most available diet is patatoes with green vegetables and beans but not everyday. I sometimes find some silver fish and mix them with the soup for the kids and make them easy to consume, it has been easy today the government has brought for us shisha kibondo, so porridge is available. There is also some money that is there to complement and make it easy to afford fruits and other needs’.*

*‘ So, there are things that have changed now, right? I used to hear that women could not eat meat back in the days, especially those of a goat. But the research has shown that it was because of greed. But it is also understandable that in the household according to how the chicken has been gotten, there is always somebody who provided, isn’t it true? And in order for that person to be able to afford the chicken, of course he must have worked for it. After all he didn’t eat out but choose to share it with his family. It should be understood that the man should get the biggest piece as he is the who worked hard to get the chicken, but also children should get their share. In addition to that, people are different, not every man does that, because according to different cultures and people’s behaviors, I know one man that used to bring meat at home and could prepare it by himself which so that he could serve each and everyone according to what he wants. There are people who say “no”, I am working for my children so that they can grow well, before you serve me first serve my children, I’ll each after I see that they are satisfied, right? So, we should consider both situation because it is the reality in the society, and it’s there, it is how it is’.*

*‘ I cannot bring my chicken and let it to my child as he/she has no idea where I got it. If I bring a chicken, it’s not only the chicken but I also bring with it a kilogram of rice, two kilograms of potatoes, and some bunches of bananas. You get that all of those things are complementary. If I bring the chicken, I have to get some piece of it, and I think that if I eat like two legs, it is not much. Then the child can get his/her share to the remaining parts of the chicken. If I have for example three children, I think each can get two pieces and their mother also could get two pieces if she wants, but the two legs should be my share as I am the one that who has provided the chicken for them all. But in brief, it’s not only the chicken I bring, but also with it I bring something else, but in my understanding the two legs should be mine’.*

*‘ For me I think that my opnion some people have mentioned it, concerning the chicken, children and their mother. You could bring it and go do something like shepherding while she is preparing it, then maybe they should not restrain themselves from eating it because you’re not around. For me, I think that the woman and the children should be the ones to eat more than me as the man of the household’.*

*‘ When the chicken is there the one who gets the biggest piece is the man because he is the one who has bought it’.*

*‘ We are in discussions here, and it is true that most of the time the man cannot buy meat and the child gets to eat first. Instead, his wife might eat first because she is the one that get to know if the food is ready by tasting it, but the biggest part should go to the husband. But it is possible also that your wife cannot leave some meat for you, that’s my idea. So, for her to solve the problem she might keep some because if you provided for your family, you should get the biggest share’.*

*‘ At my home when I bought meat, it’s not a big deal. My wife prepares it, and when it’s ready she puts it on the table and we all eat without saying that she is not the one who provided’.*

*‘ For me when I got the chance to afford the meat, my wife prepares it and when it’s ready, it’s understandable if it’s the culture or respect, she always gives me a big portion of it and I take it as it is just respecting me. And then depending on the amount of meat I brought home, after I finish eating, I give the rest to my children and their mother. Of course, I don’t eat alone I eat with my wife, but we give the leftovers to the kids’.*

*‘ Telling the truth, however things may go, when you bought the meat, present or not present you cannot get the same portion of it as the children. My wife must keep a big one for me present or not present. If it’s five pieces, each child should eat one and I get the remaining three.’*

*‘ Still on that, I cannot go against the opinion of others, but the way I see it when you provided the meat you cannot sit beside your wife and monitor the preparation process in order to be sure that she doesn’t eat some while preparing, nor can you weigh it when it’s ready to eat to make sure that the preapared amount equates to the amount you brought home, you cannot do that. If you’re a reasonable man, you cannot make an investigation trying to find out if your wife has kept three pieces out of five. It should not cause any problem. Instead the man should eat fewer meat than those who have prepared it. Everything must happen in the kitchen and you stay put waiting for whatever they’ll bring to you, you don’t have to eat a bigger portion than them.’*

*‘ Of course there the biggest part goes to the man but sometimes there might be a child that eat too much. A child that is strong that he/she can eat his/her share and then yours.’*

*‘ When it’s a child who is ready to start school, the first thing you do is take him/her to school. Then you teach them hygiene and good manners. That is what shows that you care for the child’.*

*‘ Concerning child care in our households, for example I have a three-year old child. The first thing I make sure he gets is food. Food is essential in a child’s daily life. If a child did not eat, I don’t know if you can wash him, even when you dree him it can be visible that he has a problem. First of all, child care starts by giving them enough food. The second is making sure that they are not lazy by giving them tasks at home like doing chores, but those that fit them which may not be considered as child abuse because even the Rwanda Law forbids it, for example taking a seven-year-old child to carry bricks, even that’s the cause for childhood stunting, it does not only come from malnutrition but also from heavy works given to children. So, we have to prevent that’.*

*‘ Of course, it happens! If you are doing some kind of research, you once come on the site and we will help you. You might find children that are being abused and you get confused. Child care should include avoiding giving them heavy works that may cause childhood stunting both physically and mentally. In addition to that, when you have a child at home as a parent you should reasonably make sure that he ate because hygiene is a must, and as it was mentioned if the child is at the age of going to school, you must provide him with everthing necessary for him to go to school’.*

*‘ First of all, you must pay health care for him, and then take him to a health center for tests to know what he is suffering from and get medications’.*

*‘ Still on that, in our local communities, we have community health works, when your child gets sick, you don’t immediately take him to the health center but to the community health work so that he can give him first aid’.*

*‘ When a child becomes sick you should give him a sweet potato and roast a corn for him but because he is sick, he also needs soft meals like legumes, bananas and oranges so that he can get appetite. You should try to give him food that protect the body by boosting the immune system, and then you take him to get medical care’.*

*‘ For me when my kids get sick because they lose appetite but like porridge, I try to get them porridge of different flours, then I give them soft meals with dried tiny fishes’.*

*‘ It is understandable when you are rich, I’ll come back to what my colleague said, you should have health care. That’s essential because when you have it the child cannot get too much sick at home which should be understood that you should take him to the hospital because they ae able to help him. Every time a child or anyone is sick, he/she hould be taken to the hospital. You should not wait for him to get too much sick in order to take him to the hospital’.*

*‘ When a child under five years old gets sick you have to take him to the community health work immediately, there are pills that they give him to help him get to the hospital without any problem’.*

*‘ It is taking him to the community health work, that’s the first aid we are trained about’.*

*‘ When a child is sick you immetiately notice the change compared to how he normally was when he was okey. And when you notice that you should be quick providing him with the aid of taking him to the community health work and gives him pills that would help him get to the hospital before getting too much sick’.*

*‘Those pills that the community health work give are the ones that help the child before taking him to the hospital’.*

*‘ Even though you cannot get potable water everywhere but it is available. Where I live it doesn’t take you a long journey, it’s just three meters or four to get the water. I grew up drinking source water but maybe our mothers or our old brothers can tell us about it, we are not all at the same age. They might have lived a primitive era where they could not get potable water near where they lived, but for me according to what I know from the time I started noticing everything, we were able to get potable water without any difficulties’.*

*‘ Now my family has access to potable water because there is a tap and community health works that always encourage us to drink water especially boiled one because it’s when it becomes safe for drinking. But it’s only for four years we are able to have access to potable water in our livelihood’.*

*‘ My family had been given water filter even though it’s getting old, we could put tap water in the filter and it becomes potable drinking water safe for drinking’.*

*‘ For me where I live, we can now have access to clean water. We used to fetch water from Kagoyi river, some was flowing and other from taps. Sometimes later they brought water pump, they installed some taps as my colleague mentioned about it. Before they could let us use the tap water, they provided us with water filters to make sure we are able to get drinking water. That from the taps they encouraged us to boil it before drinking it’.*

*‘ About the clean water issue, I can say that I find it when it is not raining’.*

*‘ No! We don’t even go to the river to fetch water because water tanks are full or we could trap water for ourselves’.*

*; Yes I do have a toilet’.*

*‘ As I got married in 2020, I built a house along with the latrine with a door’*

*‘ For me the latrine I use at home has a door and it is contructed in bricks and cement. We put water in a jerrycan so that when you finish to defecate you pour the water inside to flush down the feces’.*

*‘ All of us in the society we are not on the same level financially. For me I cannot say that my latrine is good, it is hundred percent clean but it’s a normal latrine. There is nothing special about it’.*

*‘ For me my latrine has a door and it is plastered and it has a roof. There is always water inside though not all of the people here use water in their latrines. There are people that say that they cannot share food with muslims because they use water to clean themselves, but we found out that it is better to use water than using papers because when you use water even your latrine takes long to get full’.*

*‘ For me my latrine is plastered with cement and I use water’.*

*‘ We use basins or cups to wash our hands. There are not many homes in which you can find the step and wash, I only see them at the church, at restaurants but in my livelihood, I’ve never seen a step and wash’.*

*‘ At home we use the step and wash’.*

*‘The main challenge we meet so many times is that even though we have taps near us, we could spend a long period of time without water in the taps. Then you hear people saying that the pumbs are not working and so. Then when that happens, we go back to using the water from the tanks’.*

*‘ Because we are used to those challenges, where I live many homes have water tanks. When you can afford one you buy it because you cannot always rely on tap water. You can even use a sheeting and fill it with water’.*

*‘ First of all, teaching requires courage. You have to sensitize people so that bad mindsets are eradicated. There are people that don’t care about anything. People that seem not to think properly. Let’s say in order to help a pregnant woman and her child to get proper meals, you should provide them with foods that contain vitamins, but some people seem not to give value to that. It should be understandable that sensitization is crucial to make sure that people understand that it’s necessary to take care of pregnant women and their children as well as themselves. If they get good mindsets even their children will be healthy’.*

*‘ We have to start by hygiene because the food and hygiene go along. When you gave both, then the mother and her child become healthy’.*

*‘ The government has done a good thing by bringing about the policy that encourages us to take care of our pregnant wives and the children, even though it’s not hundred percent. They trained community health works and then they trained people on nutrition but because they have a lot on their plates, they don’t reach to everyone or do their job as it is supposed to be done. My opinion is that they should give them some kind of a salary so that they can focus onn their work. Another thing is that not every family has access to clean water in my neighborhood. There are only two water taps in our village and most of the time there is no water, it sometimes comes and then goes. For example, for me to get water I have to walk one kilometer to get clean. It means that if my wife took such a journey to fetch water or I take a 20 litre jerrycan that should be used to wash clothes, dishes and for bathing, it immediately becomes less. If we had water near us it would save us the journey and the pregnant woman would get enough time to rest’.*

*‘ For the woman and the child to be taken care of properly the government should distribute porridge flours and some stipends to the pregnant women without considering the financial statuses’.*

*‘ My colleague there reminded me of some money that is given to pregnant and breastfeeding women that they promised us that it should be given after every one month and half or in two months, but the challenge is that we never get the money so that those it helps those mothers. Now I hear that the money has been given after eight months, which means that it will not help the mothers and their children as it was supposed to help them because it’s late, that’s a challenge. Another thing is that the pregnant mothers that are almost due are not always available. You cannot get information a regarding the distribution of the money. It would be better if that policy is professionally monitored so that each pregnant woman benefits from it’.*

*‘ About what my colleague just said, we know that a child is a child and a mother id is mother. So, sometimes you find yourself in a social status that does not match your financial status and I don’t know if it’s by mistake or something else and that has an impact on your concerning the stipends you should have been given. Finally, it causes problems to the mother and childhood stunting to the children. In the research you do you make sure that tha is considered’.*

*‘ Adding to what my colleagues just said, there are things that are done with too errors and if you try to ask for help about it to those in charge of it you don’t get the help you need. That’s one and it was talked about enough I won’t say much about it again. I would like t emphasize on something that should be understood in this way; it is not always necessary that we base our survival on the money that the government gives us, we should be able to manage some problems on our own. We don’t need to wait for the support but try to provide for ourselves. We only need to change and improve our mindsets. Let’s say for example as it is said that legumes are necessary on every meal for pregnant women and the child. Do we really have to wait for the government to give us those legumes? We should find solutions before involving the government. Is it really necessary to wait for eight months? You cannot wait for that long cause you normally have the means to live your own life. This implies that sensitization is so very necessary in order to keep reminding the people that they should learn to take care of themselves. If the legumes are necessary, why don’t we culivate them in order to use them on our meals? It does not require too much land, even on a small scale you can cultivate. The sensitization should be about reminding people that they are able to do something for themselves on their own. Some people have the legumes but tthey don’t even know how to prepare them or don’t put them on every meal. So, we should change our mindsets and I believe that it will even help the government to help us. Thank you!’*
