# Supplementary material for: “When we have served meat, my husband comes first”: A qualitative analysis of child nutrition among urban and rural communities of Rwanda
Source: PLoS One. 2024 Jul 16;19(7):e0306444. doi: 10.1371/journal.pone.0306444 (PMC11251605; doi:10.1371/journal.pone.0306444)
Supplement: Attachment — Submitted filename: PLOS Manuscript REVIEW.docx [file pone.0306444.s003.docx]

Title: “When we have served meat, my husband comes first”: A qualitative analysis of child nutrition among urban and rural communities of Rwanda.

Authors: Albin Maria, Igihozo Gloria, Musemangezhi Shuko, Namukanga Edith, Uwizeyimana Theogene, Gebremariam Alemayehu, Abebe Bekele, Wong Rex & Kalinda Chester.

Dear Authors,

Thank you for giving me the opportunity to review your manuscript entitled “When we have served meat, my husband comes first”: A qualitative analysis of child nutrition among urban and rural communities of Rwanda”. The author has executed and reported an interesting research assessing the strategies and methods used to investigate the prevalence of stunting/undernutrition among children in Rwanda. Overall, the information presented in the manuscript represents valuable information. However, the manuscript would benefit from a well-detailed description of the methodology and findings to improve understanding and flow. I feel this unique dataset has not been utilized to its full extent. Below I have provided numerous remarks on the text. Given these shortcomings the manuscript requires major revision.

**Abstract:**

- Do you refer to childhood or stunting or undernutrition as an issue of public concern
- Spell out the acronym FGD: Focus Group Discussions to allow the reader to understand
- You mean data were analyzed thematically not collected. Can you specify please the methodology used to analyse the data
- What does CHW stands for?
- Not sure if the title on husband fits as we are talking about children in the manuscript

**Introduction:**

- Thank you for your succinct introduction. No comments. I like the idea of stating why you wanted to go to investigate the issue qualitatively as opposed to quantitatively.
- Just one comment. Have you ever thought of including social cognitive theory into your introduction? This is a very interesting theory that shows there are a lot of factors that could impact person health.

**Methods:**

- Very nice to distinguish between urban and rural areas.
- Explain why you chose to do FGR with one group and in-depth interviews with the others.
- What are your stakeholders? Having a clear description is better? As I am currently confused. Have you ever thought of having a table?
- Have you used the same guide for both the in-depth interviews and the FGD?
- Where is closed room? Research facility or in the farm?
- Who conducted the interviews/FGD? The main researcher or assistant? Also, do you mean by observation notes of the farm or the interview notes in addition to recording?
- Who have done the translation? A company service or one of the researchers?
- Very beautiful Methods. Just make it clearer. You have all the information, but the flow is not there. Thank you very much
- Very beautiful using Braun and Clark. I have used it myself. Very well done. Did you perform your analysis both manually and using the software.? More information on the methodology used for both would be appreciated.

**Results:**

- Having a table would facilitate the understanding who are your key stakeholders. Pregnant women, farmers, etc.
- Very nice integration of quotes into the main text. Well-done.
- Have you thought of including if this quote is related to urban or rural area? Their views might be different. Just a thought here.
- You can also reduce some of the wording in the actual quote by putting only three dots, not four or five.

**Discussion:**

- You can say reported or stated instead of understood
- You can start your discussion with a paragraph that state/summarize the main findings of your study and then you can go to back your main findings with the literature
- Liked the ideas of using respondents, interviewees, etc.
- Very nice discussion. Just make it flow more coherently. You can also put two sections in there. Implications for research and implications for practice. I can see that you have incorporated something there about “the results from our study suggest that need for government and cooperating ……” You can put this into a new subtitle into your discussion part of the paper just to have your part easily laid-out “friendly” to flow logically
- Make sure you state more strengths. You have lots there. Such as using qualitative methodology to better understand the issue

**Conclusions:**

- Very beautiful section. No comments at all.

**References:**

- Very good. No comments at all.
